# Highly efficient and robust noble-metal free bifunctional water electrolysis catalyst achieved via complementary charge transfer

**DOI:** 10.1038/s41467-021-24829-8

**Published:** 2021-07-29

**Authors:** Nam Khen Oh, Jihyung Seo, Sangjin Lee, Hyung-Jin Kim, Ungsoo Kim, Junghyun Lee, Young-Kyu Han, Hyesung Park

**Affiliations:** 1 Department of Materials Science and Engineering, Perovtronics Research Center, Low Dimensional Carbon Materials Center, Ulsan National Institute of Science and Technology (UNIST), Ulsan, Republic of Korea; 2 Department of Energy and Materials Engineering and Advanced Energy and Electronic Materials Research Center, Dongguk University-Seoul, Seoul, Republic of Korea

**Keywords:** Energy, Hydrogen energy, Electrocatalysis

## Abstract

The operating principle of conventional water electrolysis using heterogenous catalysts has been primarily focused on the unidirectional charge transfer within the heterostructure. Herein, multidirectional charge transfer concept has been adopted within heterostructured catalysts to develop an efficient and robust bifunctional water electrolysis catalyst, which comprises perovskite oxides (La_0.5_Sr_0.5_CoO_3–*δ*_, LSC) and potassium ion-bonded MoSe_2_ (K-MoSe_2_). The complementary charge transfer from LSC and K to MoSe_2_ endows MoSe_2_ with the electron-rich surface and increased electrical conductivity, which improves the hydrogen evolution reaction (HER) kinetics. Excellent oxygen evolution reaction (OER) kinetics of LSC/K-MoSe_2_ is also achieved, surpassing that of the noble metal (IrO_2_), attributed to the enhanced adsorption capability of surface-based oxygen intermediates of the heterostructure. Consequently, the water electrolysis efficiency of LSC/K-MoSe_2_ exceeds the performance of the state-of-the-art Pt/C||IrO_2_ couple. Furthermore, LSC/K-MoSe_2_ exhibits remarkable chronopotentiometric stability over 2,500 h under a high current density of 100 mA cm^−2^.

## Abstract (web summary)

While water electrolysis offers a renewable means to obtain H_2_, it is necessary to understand the roles adopted by catalytic components. Here, authors explore a heterostructured MoSe_2_/perovskite oxide catalyst that shows multidirectional charge transfer to boost electrocatalytic water splitting.

## Introduction

Hydrogen is one of the cleanest and most sustainable energy sources that can provide high-energy-density fuel for electricity generation. Moreover, hydrogen production via electrocatalytic water splitting has been extensively explored recently^1,2^. Noble-metal-based Pt/Pd and RuO_2_/IrO_2_ electrocatalysts have been widely utilized in the cathodic hydrogen evolution reaction (HER) and anodic oxygen evolution reaction (OER), respectively, for efficient hydrogen generation from water electrolysis. Although noble-metal-based electrocatalysts exhibit excellent water electrolysis performance, these catalysts typically suffer from poor stability and scarcity in alkaline water electrolysis systems^3,4^, which is undesirable in practical industrial applications. In addition, most noble-metal-based catalysts function optimally only for the half-cell reaction (either HER or OER) under specific electrolytic conditions^5^. Bifunctional catalysts that can be simultaneously used for both the cathode and anode reactions in the same electrolyte have been developed to improve water electrolysis performance and simplify the electrolysis system^6,7^. For bifunctional catalysts, the reaction kinetics and efficiency of the OER process can determine the reaction rate of overall water electrolysis due to its sluggish kinetics from the rigid double bond in O–O and multi-proton-coupled electron transfer steps^8,9^. Therefore, achieving effective OER catalytic activity in bifunctional catalysts is critical in maximizing water electrolysis performance.

To strategically enhance the water electrolysis efficiency of the bifunctional catalyst, heterostructured catalyst configurations, including transition-metal-based nanocrystals (e.g., Co, Ni, Fe, and Cu) or noble-metal-based composites (e.g., Pt, Ag, Pd, Au, and Rh), have often been investigated for their morphology control, compositional combination of the heterostructure, and foreign elemental doping^10–14^. Charge transfer within the heterostructured catalyst, which can modulate the electronic structure of the heterostructure and directly influence the Faradaic efficiency of the respective electrode^15,16^, is critical in improving the efficiency of overall water electrolysis for bifunctional catalysts. Thus far, most bifunctional heterostructure-based catalysts have primarily utilized unidirectional charge transfer effects between heterostructure components^17,18^, which can potentially limit an optimized electronic structure to achieve ideal HER and OER catalytic activities. Therefore, a different perspective on catalyst design is needed to effectively modulate the electronic structure of the catalyst such that its electrocatalytic activity is maximized in water electrolysis. Furthermore, most bifunctional catalysts demonstrate operational stability under relatively low current densities^19–23^. As the durability of the electrolytic catalyst can be severely degraded at high current densities, e.g., through the Ostwald ripening, aggregation, and detachment during the electrolytic reaction^24,25^, robust electrochemical stability of the bifunctional catalyst should also be achieved for industrial consideration in addition to the high efficiency of water electrolysis.

In the present work, we have developed a heterostructured catalyst comprising perovskite oxide (La_0.5_Sr_0.5_CoO_3-*δ*_, LSC) and potassium ion-bonded molybdenum diselenide (K-MoSe_2_) as the bifunctional catalysts for overall water electrolysis. The semiconducting 2H-phase MoSe_2_ was moderately converted to metallic 1T-MoSe_2_ via charge transfer from potassium atoms during the potassium metal intercalation process^26^. In a previous report, Park et al. reported a heterostructured water electrolysis catalyst structure comprising perovskite oxide and transition-metal dichalcogenides (TMDs) featured with unidirectional charge transfer enabling the in-situ local phase transition in TMDs. Differently, the LSC/K-MoSe_2_ system in this study characterizes the multidirectional charge transfer phenomenon, involving two-way charge transfer from K to MoSe_2_ and from LSC to MoSe_2_, which led to significantly improved water electrolysis performance and operational stability. When K-MoSe_2_ forms a heterostructure with LSC, the metallic-phase purity of MoSe_2_ is significantly increased to over 90% through the complementary charge transfer from LSC and potassium atoms. The optimized LSC/K-MoSe_2_ catalyst exhibits significantly enhanced HER and OER performance compared with those of LSC or K-MoSe_2_, which is attributed to the increased electrical conductivity of MoSe_2_ and improved oxygen intermediate adsorption in LSC. In particular, the OER catalytic activity of LSC/K-MoSe_2_ outperforms that of the noble-metal IrO_2_ catalyst in 1 M KOH. Consequently, the performance (e.g., overpotential and Tafel slope) and energy efficiency of overall water electrolysis of the LSC/K-MoSe_2_||LSC/K-MoSe_2_ couple surpass those of the state-of-the-art Pt/C||IrO_2_ pair. Furthermore, the integrated overall water electrolysis exhibits excellent operational stability (over 2,500 h) without the decomposition of the catalyst under a high current density of 100 mA cm^−2^.

## Results

### Morphology and elemental characteristics of LSC/K-MoSe_2_

**Fig. 1:**
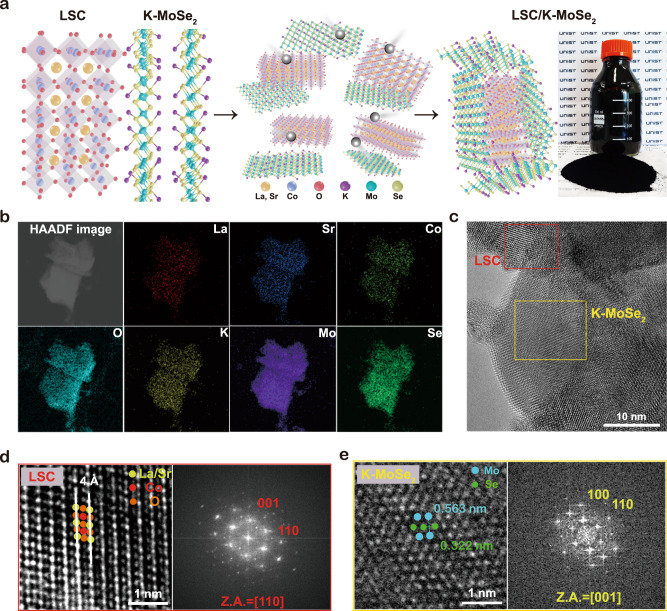
Elemental and morphological characterizations of LSC/K-MoSe_2_ heterostructure. **a** Schematic illustrating the synthesis process of LSC/K-MoSe_2_. Digital image demonstrates the large-scale synthesis capability of the proposed method. **b** HAADF-STEM image and corresponding EDS elemental mapping of La (red), Sr (blue), Co (yellow green), O (cyan), K (yellow), Mo (purple), and Se (green). **c** HR-TEM image of LSC/K-MoSe_2_, showing typical morphological characteristics of the heterostructure comprising the LSC (red square) and K-MoSe_2_ (yellow square) regions. **d** Representative HR-TEM image of LSC and corresponding FFT image, indicating the lattice structure of La/Sr, Co, and O with high crystallinity. **e** Representative HR-TEM image of K-MoSe_2_ and corresponding FFT image, exhibiting the highly crystalline lattice structure of 1T-phase MoSe_2_ comprising Mo and Se.

An LSC/K-MoSe_2_ heterostructure catalyst was synthesized via a simple and mass-producible solution process (Fig. 1a; see “Methods” section for details). In brief, LSC and K-MoSe_2_ were synthesized via the sol-gel method and molten-metal-assisted intercalation^26^, respectively; the as-prepared LSC and K-MoSe_2_ were mixed via ball milling at specified weight percent ratios. The resulting LSC/K-MoSe_2_ compounds prepared in powder and solution forms are shown in Fig. 1a. Morphological and elemental analyses were conducted via scanning electron microscopy (SEM) and transmission electron microscopy (TEM). Supplementary Fig. 1 shows the SEM image of the LSC/K-MoSe_2_ heterostructure, indicating that K-MoSe_2_ was uniformly adsorbed onto the LSC surface without aggregation, which can contribute to the high specific surface area of the heterostructure. Figure 1b shows the high-angle annular dark-field (HAADF) image of LSC/K-MoSe_2_ and the corresponding TEM energy-dispersive spectroscopy (EDS) elemental mapping profile. All relevant components (La, Sr, Co, O, Mo, Se, and K) were uniformly distributed in the heterostructure, and the elemental compositions of each component of LSC/K-MoSe_2_ obtained from the TEM–EDS spectrum are presented in Supplementary Fig. 2 with the prescribed elemental ratio. High-resolution TEM (HR-TEM) analysis was further performed to observe the atomic structure of LSC/K-MoSe_2_ in detail. As shown in Fig. 1c–e, the coexistence of LSC and K-MoSe_2_ can be clearly observed. LSC shows the well-known ABO_3_ layered perovskite oxide structure comprising La and Sr at the A site, Co at the B site, and O at the anion site, and the fast Fourier transform (FFT) pattern indicated a highly crystalline structure corresponding to the (110) and (001) planes of the LSC in the [110] zone axis (Fig. 1d). K-MoSe_2_ exhibits the typical 1T-phase atomic structure, and the FFT pattern on the [001] zone axis shows the desired crystalline structure of K-MoSe_2_ corresponding to the (100) and (110) planes (Fig. 1e). The K-MoSe_2_ flakes formed on the as-synthesized LSC/K-MoSe_2_ are a few layers in thickness, indicating that the as-exfoliated K-MoSe_2_ flakes were successfully integrated with the LSC without additional restacking (Supplementary Fig. 3).

### Electrochemical performance

**Fig. 2:**
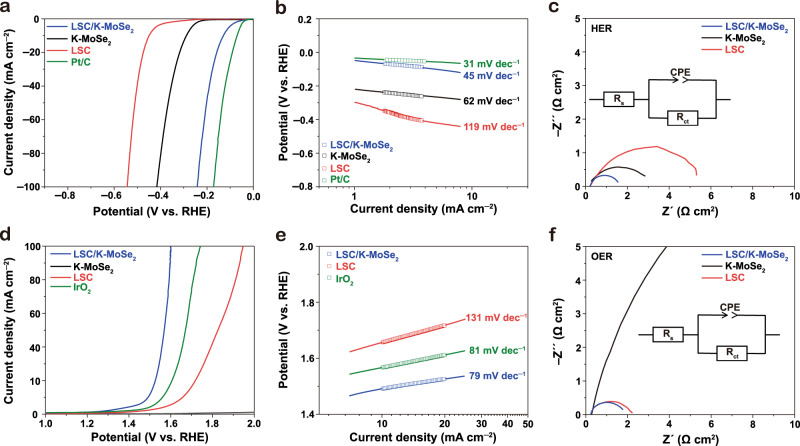
Electrocatalytic HER and OER performances of LSC/K-MoSe_2_, K-MoSe_2_, and LSC. **a**, **d** HER and OER polarization curves recorded in N_2_-saturated 1 M KOH at a scan rate of 5 mV s^−1^. **b**, **e** Corresponding Tafel slopes of the HER and OER profiles derived from the polarization curves. **c**, **f** EIS analysis of HER and OER for LSC/K-MoSe_2_, K-MoSe_2_, and LSC. The Nyquist plots comprise real (*Z*′) and imaginary (*Z*″) parts fitted as *x* and *y* axes, respectively.

The electrochemical activity of LSC/K-MoSe_2_ was investigated by analyzing the linear sweep voltammetry (LSV) curve for HER and OER, cyclic voltammograms, double-layer capacitance values, and electrochemical impedance spectroscopy (EIS) results. As shown in Supplementary Figs. 4–7, Supplementary Tables 1 and 2, various weight percent ratios of LSC and K-MoSe_2_ were first evaluated, and the optimal ratio of LSC/K-MoSe_2_ (5:4) yielding the best electrochemical catalytic performance was determined. This optimized configuration of LSC/K-MoSe_2_ heterostructure was utilized for further analyses. The hydrogen generation activity of LSC/K-MoSe_2_ was evaluated in the half-cell system, and Fig. 2a compares the polarization curves of LSC/K-MoSe_2_, K-MoSe_2_, LSC, and Pt/C in N_2_-saturated 1 M KOH using a three-electrode system. The corresponding Tafel slope of HER in various electrode configurations derived from the obtained LSV profile is shown in Fig. 2b. The Pt/C catalyst exhibited an overpotential of 68 mV at 10 mA cm^−2^ with a Tafel slope of 31 mV dec^−1^, which suggests that the Volmer–Tafel reaction is a rate-determining step^27^. In the case of LSC/K-MoSe_2_, the catalyst exhibited significantly higher HER activity than that of the LSC and K-MoSe_2_. LSC/K-MoSe_2_ showed an overpotential of 128 mV at 10 mA cm^−2^ and a Tafel slope of 45 mV dec^−1^, whereas K-MoSe_2_ and LSC required 288 and 450 mV to reach 10 mA cm^−2^ with Tafel slopes of 62 and 119 mV dec^−1^, respectively. The considerably improved Tafel slope, and thus the HER performance, of LSC/K-MoSe_2_ implies that the primary rate-determining step of the heterostructured catalyst becomes closer to the Volmer–Tafel pathway of noble metals^28,29^. The enhanced HER performance of LSC/K-MoSe_2_ was also confirmed through charge transfer resistance (R_ct_) analysis between the electrode and electrolyte, which can directly affect electrochemical performance. Figure 2c shows the Nyquist plots of LSC/K-MoSe_2_, K-MoSe_2_, and LSC for the HER obtained via EIS analysis, and the derived *R*_ct_ values were 1.30, 2.56, and 5.78 Ω cm^2^, respectively, which corroborates that charge transfer kinetics is most favorable for LSC/K-MoSe_2_ than others. We also investigated the OER activity of various catalyst configurations in N_2_-saturated 1 M KOH using a three-electrode system. Figure 2d shows the LSV curves of LSC/K-MoSe_2_, K-MoSe_2_, LSC, and IrO_2_ for OER. The corresponding Tafel slope is shown in Fig. 2e. IrO_2_, known as the best performing noble-metal-based catalyst in OER^5^, showed an overpotential of 350 mV at 10 mA cm^−2^ with a Tafel slope of 81 mV dec^−1^. Notably, the OER catalytic performance of LSC/K-MoSe_2_ exceeded that of IrO_2_ with an overpotential as low as 230 mV to reach 10 mA cm^−2^ and a Tafel slope of 79 mV dec^−1^. In contrast, LSC required an overpotential of 420 mV to reach 10 mA cm^−2^ with a Tafel slope of 131 mV dec^−1^ and K-MoSe_2_ exhibited negligible OER activity. It has been reported that the OER performance of perovskite oxide can be improved by the doping effect of molybdenum^30^. We thus conducted XPS analysis of Mo and Se in LSC/K-MoSe_2_ under OER condition after water electrolysis to elucidate the potential doping effect of Mo on the OER performance of LSC/K-MoSe_2_ (Supplementary Fig. 8 and Supplementary Table 3). The XPS results indicated partial oxidation of Mo and Se, but not the decomposition, in LSC/K-MoSe_2_ in our proposed heterostructured electrocatalyst system. In general, molybdenum oxide is known to have negligible OER activity in an alkaline environment, which is consistent with the observation of poor OER performance of K-MoSe_2_ in our study^31^. Thus, the origin of OER performance enhancement of LSC in the heterostructure is not attributed to the doping effect of molybdenum or its oxide derivatives. In our system, Mo, rather than serving as the main contributor for the OER active site, is believed to participate in the continuous charge transfer process with LSC during the OER reaction, which enhances the electrophilicity of LSC, leading to the increased adsorption of OER intermediates such as OH* and OOH*, and modulates the electronic structure of LSC/K-MoSe_2_ favorable for OER process. The improvement in OER kinetics of LSC/K-MoSe_2_ compared to that of LSC owing to the presence of Mo in the OER environment was also previously verified via experimental and computational analyses (low Tafel slope value (Fig. 2e), upshift of XPS spectra of Co 2p peak (Supplementary Fig. 14), and reduced free energy barrier and density of states (Fig. 4f–h and Supplementary Figs. 21–23)). The *R*_ct_ values of LSC/K-MoSe_2_, K-MoSe_2_, and LSC for OER obtained from the EIS analysis were 1.52, 74.0, and 1.91 Ω cm^2^, respectively (Fig. 2f). The lower *R*_ct_ in LSC/K-MoSe_2_ than K-MoSe_2_ and LSC indicates that the interfacial resistance between the electrode and electrolyte is reduced through heterostructure formation, contributing to the improved OER kinetics. These results suggest that the formation of the LSC/K-MoSe_2_ heterostructure may induce potential physicochemical interactions between the LSC and K-MoSe_2_, generating synergistic effects to lead to the observed excellent intrinsic catalytic activities for both the HER and OER processes. Below, we present detailed analyses on the heterogeneous composite structure to elucidate the origin of the enhanced catalytic performance.

### Analysis of physical properties of LSC/K-MoSe_2_

**Fig. 3:**
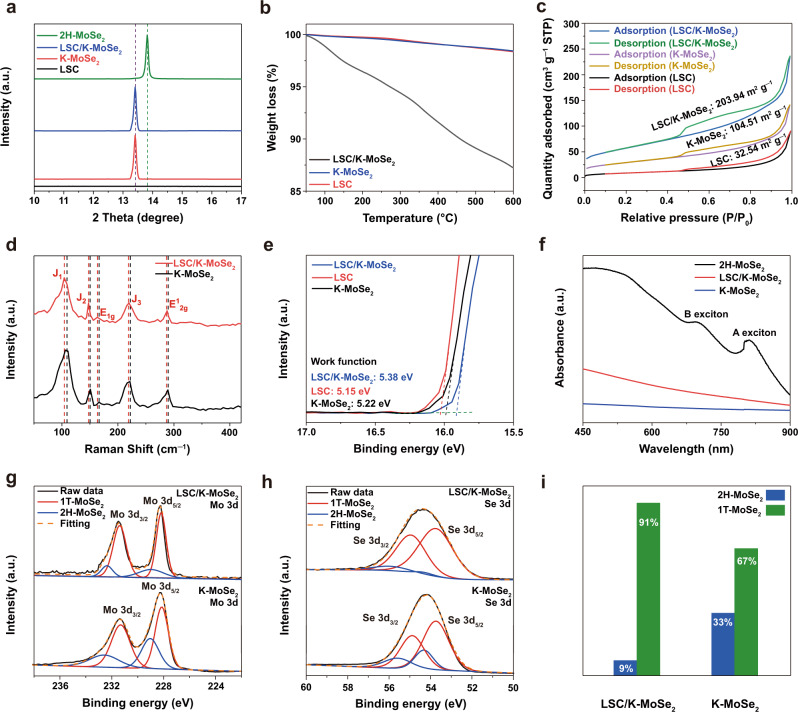
Characterization of the LSC/K-MoSe_2_ heterostructure. **a** XRD patterns for 2H-MoSe_2_, LSC/K-MoSe_2_, K-MoSe_2_, and LSC, demonstrating that each of the K-MoSe_2_ and LSC phases are well preserved in LSC/K-MoSe_2_. **b** TGA analysis of LSC/K-MoSe_2_, K-MoSe_2_, and LSC, indicating the improved surface adsorption capability of LSC/K-MoSe_2_. **c** BET surface area of LSC/K-MoSe_2_, K-MoSe_2_, and LSC obtained from N_2_ adsorption/desorption isotherms. **d** Raman spectra of LSC/K-MoSe_2_ and K-MoSe_2_, illustrating the electronic interaction between LSC and K-MoSe_2_. **e** UPS valence band spectra of LSC/K-MoSe_2_, LSC, and K-MoSe_2_. **f** UV–Vis–NIR spectra of 2H-MoSe_2_, LSC/K-MoSe_2_, and K-MoSe_2_, indicating the metallic features of LSC/K-MoSe_2_ and K-MoSe_2_. **g**, **h** High-resolution XPS spectra of Mo 3d and Se 3d peaks for LSC/K-MoSe_2_ and K-MoSe_2_. **i** Relative fraction of 2H- and 1T-MoSe_2_ in LSC/K-MoSe_2_ and K-MoSe_2_, indicating the substantial increase of the 1T-phase ratio in MoSe_2_ through complementary charge transfer.

The crystal structures of 2H-MoSe_2_, LSC/K-MoSe_2_, K-MoSe_2_, and LSC were investigated via X-ray diffraction (XRD). As shown in Fig. 3a, the preferred crystallographic orientation of 2H-MoSe_2_ at (002) plane is observed at 13.8°^32^, whereas that of K-MoSe_2_ is blue shifted to 13.4° owing to the increased interlayer spacing between the MoSe_2_ layers through the formation of potassium-intercalated MoSe_2_. The preferred orientation of the (002) plane for K-MoSe_2_ was consistently found at 13.4° for the LSC/K-MoSe_2_ heterostructure. In addition, as shown in Supplementary Fig. 9, the crystal structures of K-MoSe_2_ and LSC can be clearly identified from LSC/K-MoSe_2_, indicating that each crystal structure of K-MoSe_2_ and LSC is well preserved when forming the heterostructure through the ball mill process. Thermogravimetric analysis (TGA) was performed to examine the surface adsorption capability of the catalysts (Fig. 3b). Target materials, including LSC/K-MoSe_2_, K-MoSe_2_, and LSC, were preexposed to wet-air conditions to adsorb various environmental species in the atmosphere such as moisture, H, and OH groups. With incremental heat treatments up to 600 °C, LSC/K-MoSe_2_ exhibited a weight loss of ~14%, whereas both K-MoSe_2_ and LSC exhibited only a marginal weight loss of ~2%, demonstrating the improved surface adsorption capability of the heterostructure. Brunauer–Emmett–Teller (BET) analysis was conducted to investigate the specific surface area of LSC/K-MoSe_2_ (Fig. 3c). Compared with K-MoSe_2_ and LSC, the heterostructure exhibited significantly enhanced surface areas, i.e., 203.94, 104.51, and 32.54 m^2^ g^−1^ for LSC/K-MoSe_2_, K-MoSe_2_, and LSC, respectively, indicating that the active sites of the heterostructured catalyst can be increased to improve the efficiency of the electrochemical cell. We further analyzed the BET surface area and water electrolysis performance of various other conditioned samples comprising LSC and K-MoSe_2_ to gain more insights for the surface area effect according to different ball-milling processes on the water electrolysis performance (Supplementary Figs. 10, 11 and Supplementary Table 4). We note that, overall, there was no significant difference in the morphology for each conditioned samples before and after the ball-milling process (Supplementary Fig. 12). For the individual component materials (LSC and MoSe_2_), the ball-milled samples showed slightly increased BET surface areas, but the water electrolysis performance remained almost unaffected. When forming the heterostructured catalyst, the ball-milling process to the specific surface area and the electrolytic performance exhibited the most synergistic effects when applied during the heterostructure formation of individual component materials (i.e., LSC and K-MoSe_2_) rather than to each component of LSC and K-MoSe_2_ followed with the mixing process. These results indicate that ball milling during the formation of the composite structures increases the active sites for the water electrolysis and facilitates the charge transfer between LSC and K-MoSe_2_, thereby enabling the improved water electrolysis performance in the heterostructured catalyst. In addition, the pore size of LSC/K-MoSe_2_ was measured using the Barrett–Joyner–Halenda (BJH) method. LSC/K-MoSe_2_ exhibited a mesoporous pore size distribution of 2–50 nm (Supplementary Fig. 13). The mesoporous nature of the catalyst can effectively improve the surface area of the catalyst as well as the diffusion of the electrolyte and ionic species^33^. Further, the desorption of hydrogen and oxygen generated during the overall water electrolysis reaction becomes favorable owing to the rapid mass transfer from the electrolyte to the catalyst surface, which can help to improve the performance of the water electrolysis^34,35^.

Figure 3d shows the Raman spectra of LSC/K-MoSe_2_ and K-MoSe_2_. Characteristic vibrational Raman modes of K-MoSe_2_ were detected at 106.2, 150.5, 221.6, 165.2, and 289.7 cm^−1^, corresponding to the J_1_, J_2_, J_3_, E_1g_, and E^1^_2g_ peaks of 1T-MoSe_2_^36^. The characteristic Raman peak positions of LSC/K-MoSe_2_ were red shifted by 2 cm^−1^, indicating potential electronic interaction between the LSC and K-MoSe_2_^37^. Ultraviolet photoelectron spectroscopy (UPS) analysis was performed to investigate the charge transfer effect between LSC and K-MoSe_2_. Work function values were derived from the secondary cutoff energies. Figure 3e shows that the work function of LSC/K-MoSe_2_ increased compared to LSC and K-MoSe_2_ alone (5.38 eV vs. 5.15 and 5.22 eV). This verifies that electronic structure modulation in LSC/K-MoSe_2_ occurs via charge transfer between LSC and K-MoSe_2_. Compared with LSC, LSC/K-MoSe_2_ with an increased work function has several merits for improving electrochemical performance. The increased work function enhances the rate constant and preexponential kinetic factor in the electrocatalytic reaction^38,39^. The high rate constant and kinetic factor reduce the bond strength between active sites and adsorbed intermediates of HER on the catalyst surface, increasing the exchange current density and enabling the Gibbs free energy of hydrogen adsorbed on the active site closer to the thermoneutral point (~0 eV). It also enables overpotential reduction for the Volmer reaction owing to the increase in proton concentration of the electronic double layer of the catalyst, thereby improving HER performance^40^. UV–Vis–NIR spectroscopy analysis in Fig. 3f reveals that the A and B excitonic peaks observed for semiconducting 2H-MoSe_2_^41^ disappear in LSC/K-MoSe_2_ and K-MoSe_2_, illustrating the metal-like characteristics of the as-synthesized LSC/K-MoSe_2_ and K-MoSe_2_^42^.

X-ray photoelectron spectroscopy (XPS) analysis was performed to further elucidate the origin of the enhanced HER and OER performance of LSC/K-MoSe_2_. First, we analyzed the chemical states of Co 2*p* and O 1*s* core levels of LSC and LSC/K-MoSe_2_ to infer the cause of catalytic electrolysis performance improvement. The Co 2p peaks of LSC and LSC/K-MoSe_2_ include Co 2*p*^3+^, Co 2*p*^2+^, and two satellite features. As shown in Supplementary Fig. 14, in LSC, Co^3+^ and Co^2+^ peaks are located at 779.6/794.2 and 781.5/797.0 eV, and those of LSC/K-MoSe_2_ are located at 780.6/795.2 and 782.5/798.0 eV, respectively, illustrating an upshift of 1 eV in the peak position for the heterostructure. This upshift can be attributed to the electronic interaction between LSC and K-MoSe_2_. In LSC/K-MoSe_2_, a difference in the electronegativity between Mo and Co induces charge transfer, which can modulate the electronic structure (e_g_ orbital) of Co while maintaining the overall electroneutrality. As the e_g_-orbital filling of perovskite oxide affects the binding of oxygen-related intermediates at the active site (typically at B site), optimizing the e_g_-orbital occupancy close to 1 is critical for achieving optimal OER performance^43^. In Co, which is the B site of LSC, e_g_-orbital fillings of Co^3+^ (*t*^5^_2*g*_*e*^1^_*g*_) and Co^2+^ (*t*^5^_2*g*_*e*^2^_*g*_) are 1 and 2, respectively, which suggests that increasing the Co^3+^ proportion over Co^2+^ is preferable to obtain the optimized e_g_-orbital occupancy^44,45^. The ratio of Co^3+^/Co^2+^, the value obtained from the XPS spectra, is summarized in Supplementary Table 5; this ratio was 1.5 and 2.4 for LSC and LSC/K-MoSe_2_, respectively. Therefore, near-unity e_g_-orbital occupancy can be expected for LSC/K-MoSe_2_, confirming improved OER performance in the heterostructure. The chemical state of O 1*s* in the catalyst can also directly affect OER kinetics^46,47^. As shown in Supplementary Fig. 15, O 1*s* peaks of LSC and LSC/K-MoSe_2_ include four secondary (shoulder) peaks comprising the lattice oxygen at 528.31 eV (O_2_^−^, denoted as LO), highly oxidative oxygen species at 531.1 eV (O_2_^2−^/O^−^, denoted as OO), surface adsorbed oxygen including hydroxyl groups at 532.11 eV (O_2_/OH^−^, denoted as SO), and adsorbed molecular water at 533.2 eV (H_2_O, denoted as AW). The larger amount of surface adsorbed oxygen species compared to the lattice oxygen on the catalyst surface has a favorable effect on the formation of oxygen vacancy and rate-determining step of the OER process^46,47^. Thus, the increased SO/LO ratio of LSC/K-MoSe_2_ over LSC indicates that the formation kinetics of the active sites–O, –OH, and –OO bonds for the heterostructure was improved, which can contribute to the enhanced electrolytic performance in alkaline solutions (Supplementary Table 6).

The chemical state of MoSe_2_ was also investigated to further elucidate the origin of electrochemical performance improvement in the heterostructure. As shown in Fig. 3g, h, 1T- and 2H-phase MoSe_2_ with different relative ratios coexist in LSC/K-MoSe_2_ and K-MoSe_2_. The XPS peaks of the 1T-phase in these configurations are positioned at 228.3 and 231.4 eV for Mo 3*d*_5/2_ and Mo 3*d*_3/2_, and at 53.7 and 54.7 eV for Se 3*d*_5/2_ and Se 3*d*_3/2_, whereas those of the 2H-phase are located at 229.0 and 232.8 eV for Mo 3*d*_5/2_ and Mo 3*d*_3/2_, and at 54.3 and 55.8 eV for Se 3*d*_5/2_ and Se 3*d*_3/2_, respectively. Figure 3i summarizes the relative contents of the 1T- and 2H-phase MoSe_2_ for as-prepared LSC/K-MoSe_2_ and K-MoSe_2_ obtained from the XPS spectra. The high-purity 1T-phase MoSe_2_ (~91%) found in the heterostructure over that of K-MoSe_2_ (~67%) clearly evidence that electronic interaction occurs between LSC and K-MoSe_2_ causing the further metallic-phase transition of MoSe_2_ in K-MoSe_2_.

### Complementary charge transfer in LSC/K-MoSe_2_

**Fig. 4:**
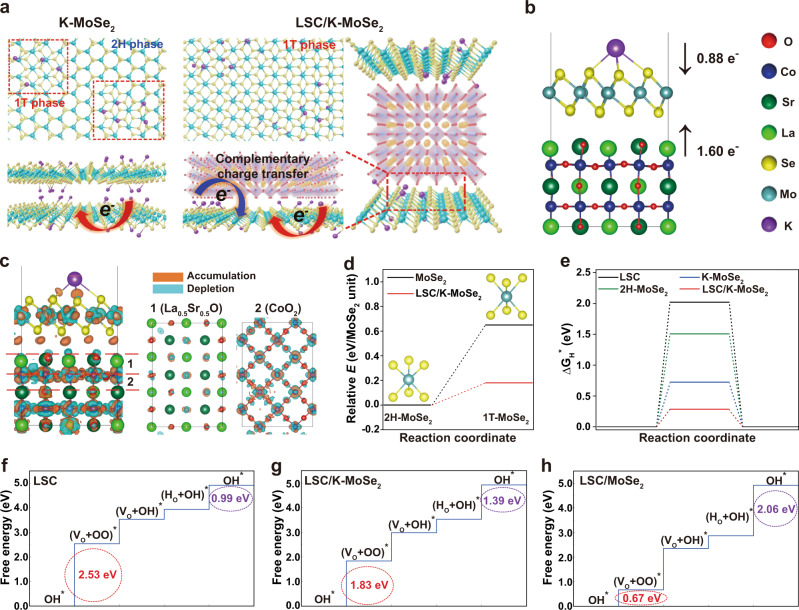
Complementary charge transfer phenomena in bifunctional LSC/K-MoSe_2_ catalysts. **a** Schematic of the atomic structure and charge transfer effect for K-MoSe_2_ and LSC/K-MoSe_2_. Complementary charge transfer in LSC/K-MoSe_2_ can modulate the electronic structure of MoSe_2_, increasing the 1T-MoSe_2_ ratio in the heterostructure. **b** Charge transfer from K and LSC to MoSe_2_ in the optimized LSC/K-MoSe_2_ heterostructure. **c** Charge density difference plot for the interface between LSC and K-MoSe_2_. Side view of the total structure (left) and the cross section of the layers of La_0.5_Sr_0.5_O (middle) and CoO_2_ (right) are demonstrated. **d** Relative energies of 1T-MoSe_2_ in monolayer structure (black line) and LSC/K-MoSe_2_ heterostructure (red line) compared to the energy of the 2H-MoSe_2_ monolayer. **e** Free energy diagrams for HER for LSC, 2H-MoSe_2_, K-MoSe_2_, and LSC/K-MoSe_2_. **f**–**h** Free energy diagrams for OER for LSC, LSC/K-MoSe_2_, and LSC/MoSe_2_.

We hypothesize that the drastic increase in the 1T-phase content of MoSe_2_ in the heterostructured catalyst occurs because of the complementary charge transfer between LSC and K-MoSe_2_, as schematically illustrated in Fig. 4a. K-MoSe_2_ is synthesized by intercalating the potassium metal within the MoSe_2_ interlayers, followed by subsequent exfoliation, where potassium atoms and Se form K–Se ionic bonds. The large electronegativity difference (1.73) between K and Se promotes the charge transfer from K to Se, modulating the Mo 4*d* orbital configuration of MoSe_2_ from the occupied 4*d*_*z*_^2^ level to incompletely filled 4*d*_*xz*_, 4*d*_*yz*_, and 4*d*_*yx*_ orbitals. This electronic structure rearrangement causes local phase transition from 2H-MoSe_2_ to 1T-MoSe_2_. Following the formation of the LSC/K-MoSe_2_ heterostructure, charge transfer from the Co of LSC to the 2H-MoSe_2_ portion of K-MoSe_2_ occurs, causing an additional 1T-phase transition in MoSe_2_. This complementary charge transfer in LSC/K-MoSe_2_ has beneficial effects on both HER and OER performance. The increased 1T-phase MoSe_2_ concentration in LSC/K-MoSe_2_ creates a more electron-rich and electrically conducting surface, enabling fast electron transfer at the catalytically active site, thereby improving HER kinetics^48^. The Co in LSC, after donating the electrons to K-MoSe_2_, becomes more electrophilic; such charge transfer results in the upshift of the *d*-band center and improved adsorption capability of oxygen-generating intermediates (e.g., O*, OH*, OO*) at the catalyst surface^49^, thereby enhancing OER kinetics.

We computationally analyzed charge transfer and catalytic activities in LSC/K-MoSe_2_ using density functional theory (DFT) calculations. The evaluated charge transfer into a MoSe_2_ monolayer from Bader charge analysis^50^ is 2.48 *e* (0.88 *e* and 1.60 *e* from the K atom and LSC perovskite, respectively) in the LSC/K-MoSe_2_ structure model (Fig. 4b). We found that the charge transfer from LSC to MoSe_2_ is unaffected by the K atoms because the LSC to MoSe_2_ charge transfer in the LSC/MoSe_2_ structure is the same value of 1.60 *e*, indicating that the complementary charge transfer from K and LSC into MoSe_2_ is available in the LSC/K-MoSe_2_ system. The additional electron transfer from K into MoSe_2_ layers can lead to a larger portion of 1T-MoSe_2_ in LSC/K-MoSe_2_ than in LSC/MoSe_2_, supporting the experimentally observed results (Fig. 3i). We found that the charge transfer from LSC to MoSe_2_ mainly occurs in the CoO_2_ subsurface layer of LSC as depicted in Fig. 4c, wherein drastic changes in the electron density are observed in the CoO_2_ layer; these changes are expected to boost OER performance with a change in the electronic structure of LSC layers. Charge transfer from the CoO_2_ layer to MoSe_2_ was also confirmed via the density of states (DOS) analysis presented in Supplementary Fig. 16 and Supplementary Note 2. The CoO_2_ layer under the outermost La_0.5_Sr_0.5_O surface plays an important role in charge transfer while electrocatalytic activities occur on the La_0.5_Sr_0.5_O surface, which is essential in explaining the superiority of LSC perovskite. Furthermore, we found that the complementary charge transfer from K and LSC into MoSe_2_ layers markedly reduced the energy barrier when MoSe_2_ underwent 2H- to 1T-phase transition, as shown in Fig. 4d (0.65 eV in MoSe_2_ to 0.18 eV in LSC/K-MoSe_2_); this indicates that a high portion of 1T-MoSe_2_ phase will be present in the LSC/K-MoSe_2_ heterostructure.

To further elucidate the enhanced water-splitting performance in LSC/K-MoSe_2_ heterostructure, we analyzed the reaction-free energy for subreactions in both HER and OER. The values of Δ*G*_H*_ can directly evaluate HER performance^51–53^. Figure 4e shows a comparison of Δ*G*_H*_ in LSC/K-MoSe_2_ with LSC, 2H-MoSe_2_, and K-MoSe_2_. The LSC/K-MoSe_2_ structures are suitable catalysts for providing the most favorable HER environment for H_2_ production with the smallest Δ*G*_H*_ of 0.25 eV, whereas the Δ*G*_H*_ values for LSC, 2H-MoSe_2_, and K-MoSe_2_ are 2.01, 1.44, and 0.69 eV, respectively. The optimal near-zero Δ*G*_H*_ in LSC/K-MoSe_2_ is associated with the increased number of states near the energy of normal hydrogen electrode (NHE) potential (Supplementary Fig. 17), thus increasing the interactions between the hydrogen s orbital and the 1T-MoSe_2_ states.

Excellent OER performance in LSC/K-MoSe_2_ was also explained through atomic-level simulations. Figure 4f–h shows the free energy diagrams for OER in LSC, LSC/K-MoSe_2_, and LSC/MoSe_2_, respectively. Our DFT calculations strongly suggest that the OER reactions proceed with the lattice-oxygen participation mechanism^54,55^ dominantly rather than the conventional adsorbate evolving mechanism in the LSC/K-MoSe_2_ system (Supplementary Fig. 19), and the free energy barriers of reaction-determining steps were evaluated to be 2.53, 2.06, and 1.83 eV for LSC, LSC/MoSe_2_, and LSC/K-MoSe_2_, respectively. Taking previously reported DFT-calculated OER free energy barriers (2.05−2.19 eV)^56–58^ in IrO_2_ catalysts into account, the order of computed free energy barriers (LSC > IrO_2_ > LSC/K-MoSe_2_) agrees well with the experimental results where the OER onset potentials decrease according to same descending order of LSC > IrO_2_ > LSC/K-MoSe_2_, as shown in Fig. 2d. The reduced free energy barrier in the LSC/K-MoSe_2_ originates from the well-balanced free energies between two governing reactions: (1) OH* to (V_O_ + OO)* + H^+^ + *e*^−^ and (2) (H_O_ + OH)* to OH* + H^+^ + *e*^−^, where OH*, (V_O_ + OO)*, and (H_O_ + OH)* indicate OH absorbed on LSC surface, OO adsorbed on LSC surface with a neighboring oxygen vacancy, and OH adsorbed on LSC surface with a H atom adsorbed on a neighboring lattice O atom, respectively (see Supplementary Fig. 20 for the detailed atomic structures). While the free energy changes in reaction (1) (the red dotted circle) decrease on the order of LSC (2.53 eV) > LSC/K-MoSe_2_ (1.83 eV) > LSC/MoSe_2_ (0.67 eV), those in reaction (2) (the violet dotted circle) increase in the opposite order; LSC (0.99 eV) < LSC/K-MoSe_2_ (1.39 eV) < LSC/MoSe_2_ (2.06 eV), effectively producing the lowest free energy barrier in LSC/K-MoSe_2_ with a remarkably ideal balance between the rate-determining steps of reactions (1) and (2). Reaction (1) involves the replacement of the hydrogen in the OH adsorbate with the lattice oxygen that escapes from the lattice, leaving an oxygen vacancy. Therefore, the free energy barrier in reaction (1) is strongly affected by the energy difference between O* and (V_O_ + OO)* structures^59^ as evaluated in Supplementary Fig. 21, wherein (Vo+OO)* structures are energetically more unfavorable in the same order with the free energy barriers as that in reaction (1); see DOS plots in Supplementary Fig. 22 for additional information on the instability of (V_O_ + OO)* in LSC. In the case of reaction (2), the free energy barrier is related to how easily a hydrogen atom can be detached from the lattice-oxygen atom; see Supplementary Fig. 23, wherein the energy difference between (H_O_ + OH)* and OH* increases on the same order of the free energy barriers as that in reaction (2).

### Overall water electrolysis of the LSC/K-MoSe_2_ couple

**Fig. 5:**
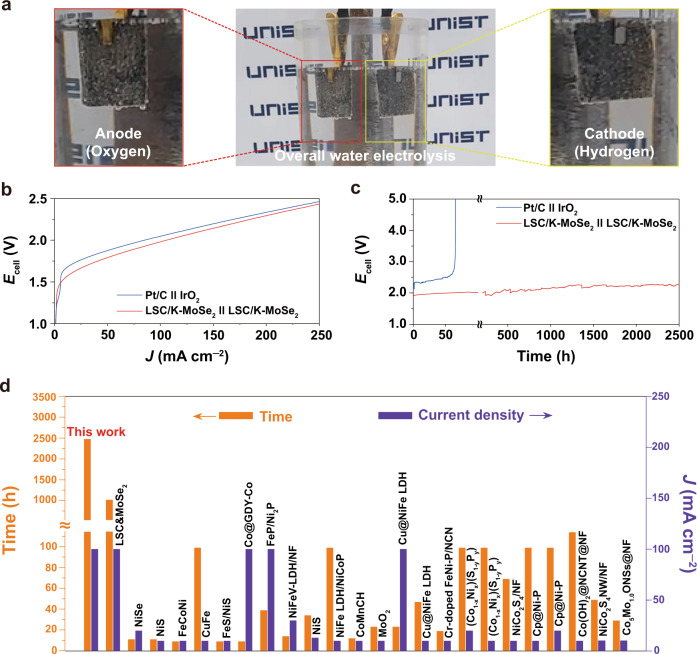
Overall water electrolysis of LSC/K-MoSe_2_. **a** Digital image of full-cell water-splitting system comprising a two-electrode configuration with LSC/K-MoSe_2_||LSC/K-MoSe_2_. **b**, **c** LSV and chronopotentiometric durability curves of Pt/C||IrO_2_ and LSC/K-MoSe_2_||LSC/K-MoSe_2_ measured in 1 M KOH. **d** Summary of the overall water electrolysis stability of LSC/K-MoSe_2_||LSC/K-MoSe_2_ and other reported electrocatalyst couples.

The developed LSC/K-MoSe_2_ demonstrated excellent intrinsic bifunctional catalytic activity in alkaline electrolytes for both HER and OER. Herein, the overall water electrolysis of the LSC/K-MoSe_2_ configuration (*i.e*., LSC/K-MoSe_2_ used for both cathode and anode, denoted as LSC/K-MoSe_2_||LSC/K-MoSe_2_) was examined to further demonstrate the overall water-splitting performance and stability of LSC/K-MoSe_2_ as the bifunctional electrocatalyst in N_2_-saturated 1 M KOH solution. Efficient generation of the hydrogen (cathode) and oxygen (anode) gases using the LSC/K-MoSe_2_||LSC/K-MoSe_2_ couple was confirmed, as illustrated in Fig. 5a and Supplementary Movie 1. Figure 5b shows the cell voltage (*E*_cell_ = *E*_anode_ − *E*_cathode_) measurement results of Pt/C||IrO_2_ (Pt/C for cathode and IrO_2_ for the anode) and LSC/K-MoSe_2_||LSC/K-MoSe_2_ obtained during the water electrolysis reaction. Consistent with the result of the half-cell-configured polarization profiles, the LSC/K-MoSe_2_||LSC/K-MoSe_2_ couple demonstrated better overall water electrolysis performance than the state-of-the-art noble-metal-based Pt/C||IrO_2_ couple. The cell voltages needed to attain 10 and 100 mA cm^−2^ were 1.59 and 1.95 V for LSC/K-MoSe_2_||LSC/K-MoSe_2_ and 1.67 and 2.04 V for Pt/C||IrO_2_. Although the HER performance of LSC/K-MoSe_2_ in the half-cell reaction was slightly lower than that of Pt/C, the overwhelmingly high OER performance resulted in excellent overall water electrolysis activity for LSC/K-MoSe_2_, surpassing that of the noble-metal pair Pt/C||IrO_2_. In addition to performance, electrochemical stability is an equally important criteria to consider when promoting the broad industrial pertinence of water electrolysis catalysts. To examine the electrochemical stability of LSC/K-MoSe_2_, its chronopotentiometric profile was measured at a high current density of 100 mA cm^−2^. As shown in Fig. 5c, for the Pt/C||IrO_2_ reference, a rapid increase in cell voltage, which indicates cell failure, was observed within 60 h under our experimental test conditions. However, the LSC/K-MoSe_2_||LSC/K-MoSe_2_ couple exhibited exceptionally high electrochemical durability even after 2,500 h of continuous operation without noticeable performance degradation. To verify the excellent operational durability of LSC/K-MoSe_2_ as the electrocatalyst, its physical and chemical characteristics were investigated after 2,500 h of stability testing. Supplementary Fig. 24 shows the SEM image of the catalyst electrode before and after the stability test. Even after 2,500 h of the electrocatalytic reaction, the starting electrode structure was well preserved without any significant physical damages or detachment. XPS analysis further reveals the superior chemical stability of LSC/K-MoSe_2_ (Supplementary Fig. 25 and Supplementary Tables 7, 8). After 2,500 h of overall water electrolysis reaction, each ratio of the Co^3+^/Co^2+^ in Co 2*p* and surface-active oxygen/lattice oxygen in O 1s exhibited almost negligible changes compared with those of pristine LSC/K-MoSe_2_. MoSe_2_ may potentially be decomposed into molybdenum oxides and selenate at the potentials of the OER electrode during prolonged electrolysis^60^. To verify the stability of MoSe_2_ in LSC/K-MoSe_2_, we performed XPS analysis for Mo and Se in LSC/K-MoSe_2_ on the OER electrode after 2,500 h of chronopotentiometric stability test. Although some partial oxidization was observed, the integrity of MoSe_2_ was well preserved without decomposition (Supplementary Fig. 26 and Supplementary Table 9). Moreover, the energy efficiency of the overall water electrolysis using LSC/K-MoSe_2_||LSC/K-MoSe_2_ at 100 mA cm^−2^ was calculated to be 75.4% (Supplementary Note 1). Considering the energy efficiency of a typical noble-metal-based water electrolysis catalyst is around 70%, our results demonstrate that the developed bifunctional LSC/K-MoSe_2_ catalyst can be used as a promising electrocatalyst in water electrolysis for efficient hydrogen production. Figure 5d (also summarized in Supplementary Table 10) compares the electrochemical stability of overall water electrolysis for various catalyst configurations reported to date along with the proposed LSC/K-MoSe_2_ in this work. Additionally, we further investigated the operational durability of the LSC/K-MoSe_2_ in various harsh environments. Chronopotentiometric stability test of the LSC/K-MoSe_2_ couple was conducted under high operational temperature and current density (500 and 1,000 mA cm^–2^ in 1 M KOH at 60 °C) and high electrolyte concentration (100 mA cm^–2^ in 10 M KOH at room temperature). In 1 M KOH at 60 °C, the LSC/K-MoSe_2_ couple required cell voltages of 2.25 V at 500 mA cm^–2^ and 2.52 V at 1000 mA cm^–2^, respectively (Supplementary Fig. 27). Under such conditions, it exhibited stable operational stability over 1,200 and 800 h at 500 and 1,000 mA cm^–2^, respectively, without obvious performance degradation (Supplementary Fig. 28). In 10 M KOH at room temperature, the cell voltage needed to achieve 100 mA cm^–2^ was 1.87 V for the LSC/K-MoSe_2_ couple (Supplementary Fig. 29). In the two-electrode cell, the chronopotentiometric stability of 1,600 h was achieved at 100 mA cm^–2^ in 10 M KOH at room temperature without any noticeable performance degradation (Supplementary Fig. 30). Despite the various accelerated test conditions, our heterostructure demonstrates overwhelmingly superior durability for water electrolysis.

## Discussion

In this work, we developed a heterostructure-based electrocatalyst that demonstrates excellent overall water electrolysis performance and stability. The complementary charge transfer induced within the heterogeneous catalyst, comprising alkali-metal-treated transition-metal dichalcogenides and perovskite oxide, generated synergistic effects in both HER and OER processes. The modulated electronic structure of MoSe_2_ with high metallic-phase purity and improved electrical conductivity enhanced HER kinetics. The increased electrophilicity of LSC improved the adsorption capability of oxygen-generating intermediates on the catalyst surface, thereby boosting OER performance over that of IrO_2_. The water electrolysis performance using the LSC/K-MoSe_2_||LSC/K-MoSe_2_ couple outperformed the state-of-the-art noble-metal pair of Pt/C||IrO_2_, exhibiting lower cell voltage for the overpotential at 10 and 100 mA cm^−2^, improved energy efficiency, and excellent operational stability over 2,500 h. This work can provide a promising perspective for the performance maximization of heterostructure-based catalysts in water electrolysis to substitute precious-metal-based electrocatalysts.

## Methods

### Synthesis of K-MoSe_2_

For the preparation of K-MoSe_2_, 500 mg of bulk MoSe_2_ (<2 μm, purity >99%, Alfa Aesar) and 200 mg of potassium metal (stored in oil, purity >99.95%, Kojundo, Korea) were added to a glass tube under inert conditions in a glovebox. The tube was sealed and treated at 400 °C for 1 h. The as-prepared potassium-intercalated MoSe_2_ was rinsed with deionized water and ethanol to remove potassium ion residues. The resulting powder was dried at 80 °C for 24 h.

### Synthesis of LSC

To synthesize the LSC perovskite oxides, an aqueous solution containing dissolved La, Sr, and Co nitrites (La(NO_3_)_3_ ∙ 6H_2_O, Sr(NO_3_)_2_, Co(NO_3_)_2_ ∙ 6H_2_O, Alfa Aesar) in stoichiometric amounts and citric acid (C_6_H_8_O_7_, Sigma-Aldrich) in deionized water was prepared. Post solvent evaporation, the resulting wet-gel was calcined at 900 °C for 2 h and at 950 °C for 10 h to remove the organic fraction. Finally, the resulting reaction product was mortared to homogenize the LSC.

### Synthesis of LSC/K-MoSe_2_

To synthesize the LSC/K-MoSe_2_ heterostructure, as-prepared LSC and K-MoSe_2_ were high-energy milled with 10 wt.% of Ketjen Black (KB) (EC-600JD, Lion Specialty Chemicals Co. Ltd.) via the planetary ball mill system (PM-100, Retsch). To find the optimized weight ratio for LSC and K-MoSe_2_ for electrochemical performance, various mixture weight ratios were examined with LSC:K-MoSe_2_:KB of 8:1:1, 7:2:1, 6:3:1, and 5:4:1. The total weight of the mixture was maintained as 600 mg. LSC, K-MoSe_2_, and KB in ethanol were sealed into the steel jar and ball-milled at 500 rpm for 2 h. After completing the ball-milling process, the resulting product was thoroughly dried and collected.

### Material characterizations

The morphology and EDS elemental mapping of the catalysts were characterized using HR-TEM (JEM-2100F, JEOL) with an accelerating voltage of 200 kV. Crystallographic information of catalysts was analyzed through high-power XRD (D/MAX2500V/PC, Rigaku) at 40 kV and 200 mA at a scanning rate of 1° min^−1^ in the diffraction range of 10°–80°. The chemical state and work function were investigated via XPS (ESCALAB 250XI, Thermo Fisher Scientific) with monochromated Al-Kα radiation. TGA analysis was conducted to investigate the surface adsorption capacity of catalysts at a ramping temperature rate of 10 °C min^−1^ using thermogravimetric analyzer (Q500, TA). The surface area and pore size of the catalysts were evaluated using N_2_ desorption/adsorption isotherms of catalysts using a physisorption analyzer (ASAP 2420, Micromeritics Instruments). The optical properties of the catalysts were collected using a UV–Vis–NIR spectrophotometer (Cary 5000, Agilent). Raman spectra were recorded using confocal Raman spectroscopy (Alpha300R, WITec) equipped with a 532-nm laser. The electrode morphologies before and after overall water electrolysis were obtained via cold FE-SEM (S-4800, HITACHI).

### Electrochemical measurements

Half-cell electrochemical measurements were performed in 1 M KOH, in which saturated Ag/AgCl and a carbon rod were used as the reference and counter electrodes, respectively, in a three-electrode configuration controlled by an electrochemical workstation (CHI 760E, CH Instruments Inc.). Catalyst ink including LSC/K-MoSe_2_, K-MoSe_2_, and LSC was prepared by dispersing 9 mg of the catalyst and 1 mg of KB in a 1 mL binder solution comprising 5 wt.% Nafion solution (Sigma-Aldrich), ethanol, and isopropyl alcohol, followed by bath sonication. A catalyst ink of Pt/C and IrO_2_ was similarly prepared except for the KB. The working electrode was prepared via drop-casting 5 µL of the as-prepared catalyst inks onto the glassy carbon disk electrode with an area of 0.071 cm^2^. The HER and OER LSV polarization curves were obtained at a scan rate of 5 mV s^−1^ in N_2_-saturated electrolyte, which were measured from 0 to −1.0 V (vs. reversible hydrogen electrode (RHE)) and from 1.0 to 2.0 V (vs. RHE), respectively. Tafel slope values were derived from the LSV curves by plotting the overpotential against current density in log-scale from 1 to 10 mA cm^−2^. All potentials in this work were measured with respect to Ag/AgCl reference electrode and converted to RHE scale using the following formula in 1 M KOH (pH 14): *E*(vs.RHE) = *E*(vs. Ag/AgCl) + *E*_Ag/AgCl_ (=0.197 V) + 0.0592 pH = *E*(vs. Ag/AgCl) + 1.0258 V. EIS measurements were conducted at an overpotential of −0.2 and 0.7 V (vs. RHE) for HER and OER, respectively, in a frequency range of 100 kHz to 0.01 Hz with an amplitude of 10 mV in 1 M KOH. All half-cell polarization curves were corrected for ohmic losses by the following equation. *E* = *E*(RHE) – *i*R_s_, where *E* is the potential after the *iR*-correction, *E*(RHE) is the measured potential with respect to RHE (before *iR-*correction), *i* is the measured current, and *R*_s_ is the uncompensated resistance obtained from EIS analysis. To measure the double-layer capacitance (*C*_dl_) value, the potential window of cyclic voltammograms was cycled in the non-Faradaic region from 0.03 to 0.33 V (vs. RHE) with different scan rates from 20 to 160 mV s^−1^. *C*_dl_ values were derived by plotting the charging current density difference (Δ*j* = (*j*_a_ − *j*_c_)/2) at 0.18 V. Overall water electrolysis testing was conducted using a two-electrode configuration comprising electrosprayed catalyst inks on Ni foam (with a catalyst loading of 1 mg cm^−2^) as the current collector. Chronopotentiometry stability test results of the overall water electrolysis were obtained under a current density of 100 mA cm^−2^ using an electrochemical workstation (ZIVE BP2C, Wonatech Co., Ltd.).

### Computational details

We performed spin-polarized ab initio calculations using the Vienna ab initio simulation package (VASP)^61^ within the projector augmented wave (PAW) method^62^ and Perdew–Burke–Ernzerhof (PBE)^63^ exchange and correlation functionals. The DFT+*U* method based on Dudarev’s approach^64^ was adopted with *U* = 4.3 and *J* = 1.0 eV (*U*_eff_ = 3.3 eV) for Co-3*d* and *U*_eff_ = 4.0 eV for Mo-4*d*, as employed in previous studies^65,66^. First, the LSC slab structure was prepared based on 2√2 × 3√2 × 2 supercells with >20 Å vacuum space. A 1 × 1 × 1 Monkhorst-pack *k*-points mesh was adopted with 2 × 10^−2^ eV Å^−1^ for force criterion in the ionic relaxations and a 400 eV energy cutoff for the plane-wave basis set was used with valance electron configurations of 5*s*^2^5*p*^6^5*d*^1^6*s*^2^ (La), 4*s*^2^4*p*^6^5*s*^2^ (Sr_sv), 3*d*^8^4*s*^1^ (Co), 2*s*^2^2*p*^4^ (O), 4*s*^2^4*p*^6^4*d*^5^5*s*^1^ (Mo_sv), 4*s*^2^4*p*^4^ (Se), and 3*s*^2^3*p*^6^4*s*^1^ (K_sv) orbitals for La, Sr, Co, O, Mo, Se, and K, respectively. The LSC/MoSe_2_ and LSC/K-MoSe_2_ structures were modeled using 2 × 5 supercell of 2H- or 1T-MoSe_2_ monolayer placed on the LSC (001) surfaces with <3% lattice mismatch for LSC/K-MoSe_2_ (the length of the cell-vectors: *a* = 11.20 Å, *b* = 16.57 Å, and *c* = 35.00 Å), K atoms were attached on the MoSe_2_ monolayer at the energetically most stable site, where one K atom was adsorbed on the 2 × 5 MoSe_2_ supercell surface (Fig. 4b). The top two atomic layers of LSC slab structure were relaxed to simulate the heterostructures. The charge transfer in LSC/K-MoSe_2_ structure was analyzed using Bader population analysis^50^. Second, the free energy of HER reactions was computed until the residual force components were within 5 × 10^−3^ eV Å^−1^ using the equation *G*_H_ = *E*(H) + 0.24 eV, wherein *E*(H) is the adsorption energy of a H atom, calculated for $1/2$H_2_ at pH = 0 and *p*(H_2_) = 1 bar, and 0.24 eV correction is for the differences in zero-point-energy and entropy^67,68^. The free energy of each subreaction for OER was evaluated using the equation *G* = Δ*E* + ΔZPE − *T*Δ*S* at pH = 0, *T* = 298 K, and zero applied potential (0 V vs. RHE), where Δ*G*, Δ*E*, ΔZPE, *T*, and Δ*S* represent the change in free energy, total energy difference, change in zero-point-energy, temperature, and change in entropy, respectively. The ΔZPE and *T*Δ*S* terms were adopted from previous studies and gas phase H_2_O and O_2_ were considered references for all the reactions, assuming 0.035 bar H_2_O gas pressure at room temperature for equilibrium with liquid water. The free energy O_2_ gas was computed by fixing the free energy changes in the overall reaction (H_2_O → $1/2$O_2_ + H_2_) to the experimentally measured value, 2.46 eV, as adopted in previously reported OER computations^59,69–73^. To model OER on LSC (001) surfaces in LSC/K-MoSe_2_, we used the cropped 2 × 1.5 MoSe_2_ supercells to expose the LSC (001) surface to secure enough space for OER adsorbates on the LSC surface. A 4 × 4 × 1 Monkhorst-pack *k*-points mesh was used to analyze the DOS.

## Supplementary information

Supplementary Information_No highlight

Peer Review File

Supplementary Movie 1

Description of additional supplementary files

## Data Availability

The data measured, simulated, and analyzed in this study are available from the corresponding author on reasonable request.
