# Supplementary material for: Highly efficient and robust noble-metal free bifunctional water electrolysis catalyst achieved via complementary charge transfer
Source: Nat Commun. 2021 Jul 29;12:4606. doi: 10.1038/s41467-021-24829-8 (PMC8322133; doi:10.1038/s41467-021-24829-8)
Supplement: Supplementary file 1 — Supplementary Information_No highlight [file 41467_2021_24829_MOESM1_ESM.pdf]

Supplementary Information

Highly Efficient and Robust Noble-metal Free Bifunctional Water Electrolysis Catalyst Achieved via Complementary Charge Transfer

Nam Khen Oh *et al*.

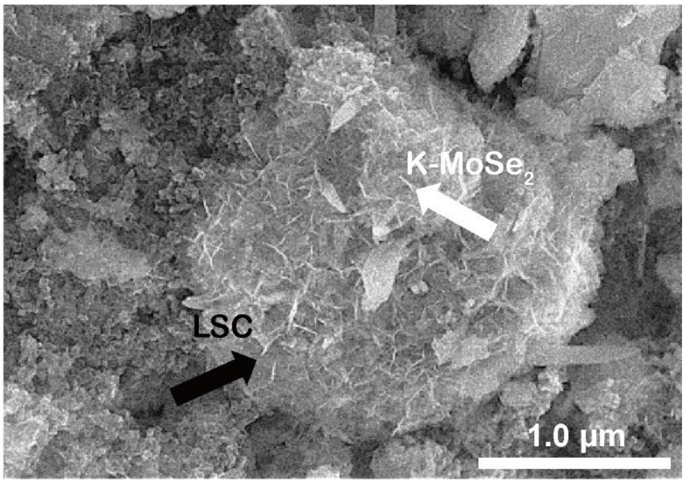

Supplementary Figure 1 SEM image of LSC/K-$MoSe_2$, showing uniform distribution of K-$MoSe_2$ flakes on LSC without aggregation.

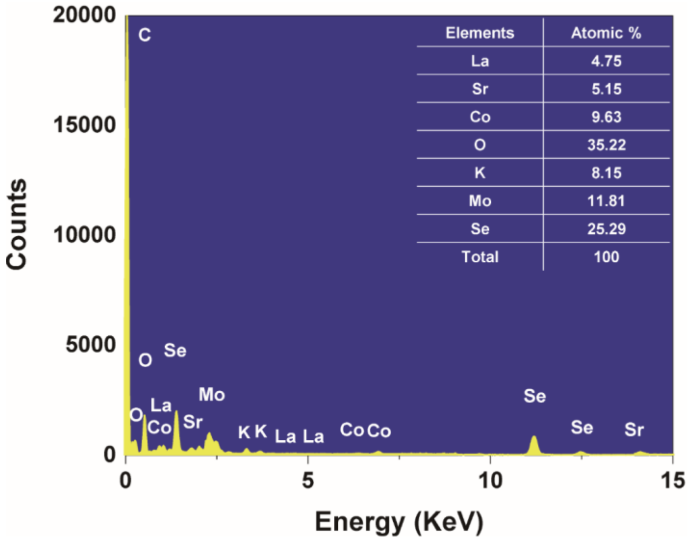

Supplementary Figure 2 TEM-EDS spectrum of as-prepared LSC/K-$MoSe_2$ shown with the atomic percentage of the elemental composition.

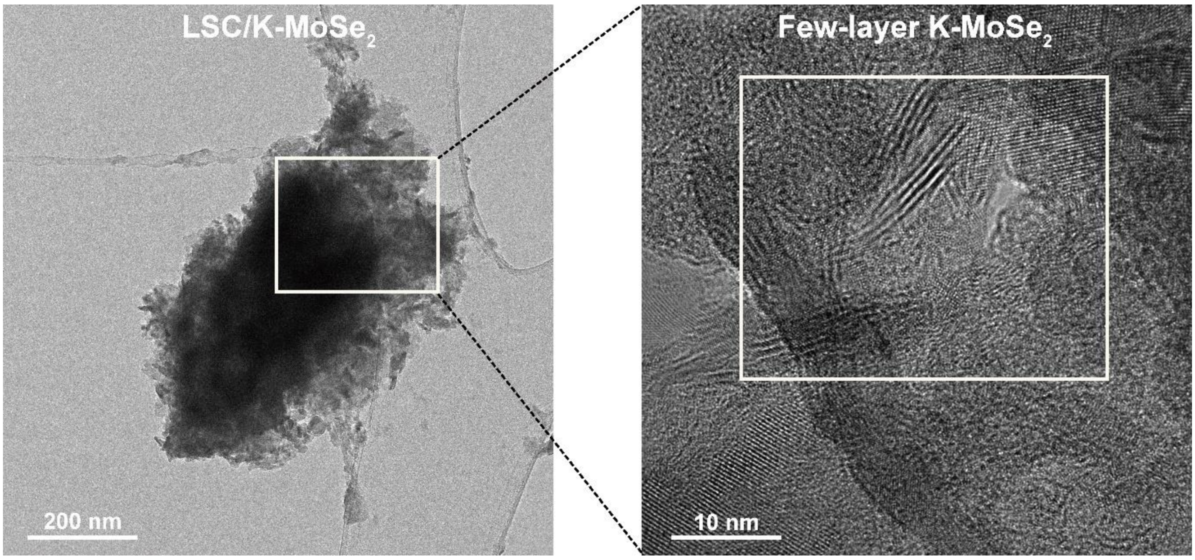

Supplementary Figure 3 TEM images of LSC/K-$MoSe_2$. The zoom-in image (right) of the white square region (left) verifies the presence of few-layer K-$MoSe_2$ in LSC/K-$MoSe_2$.

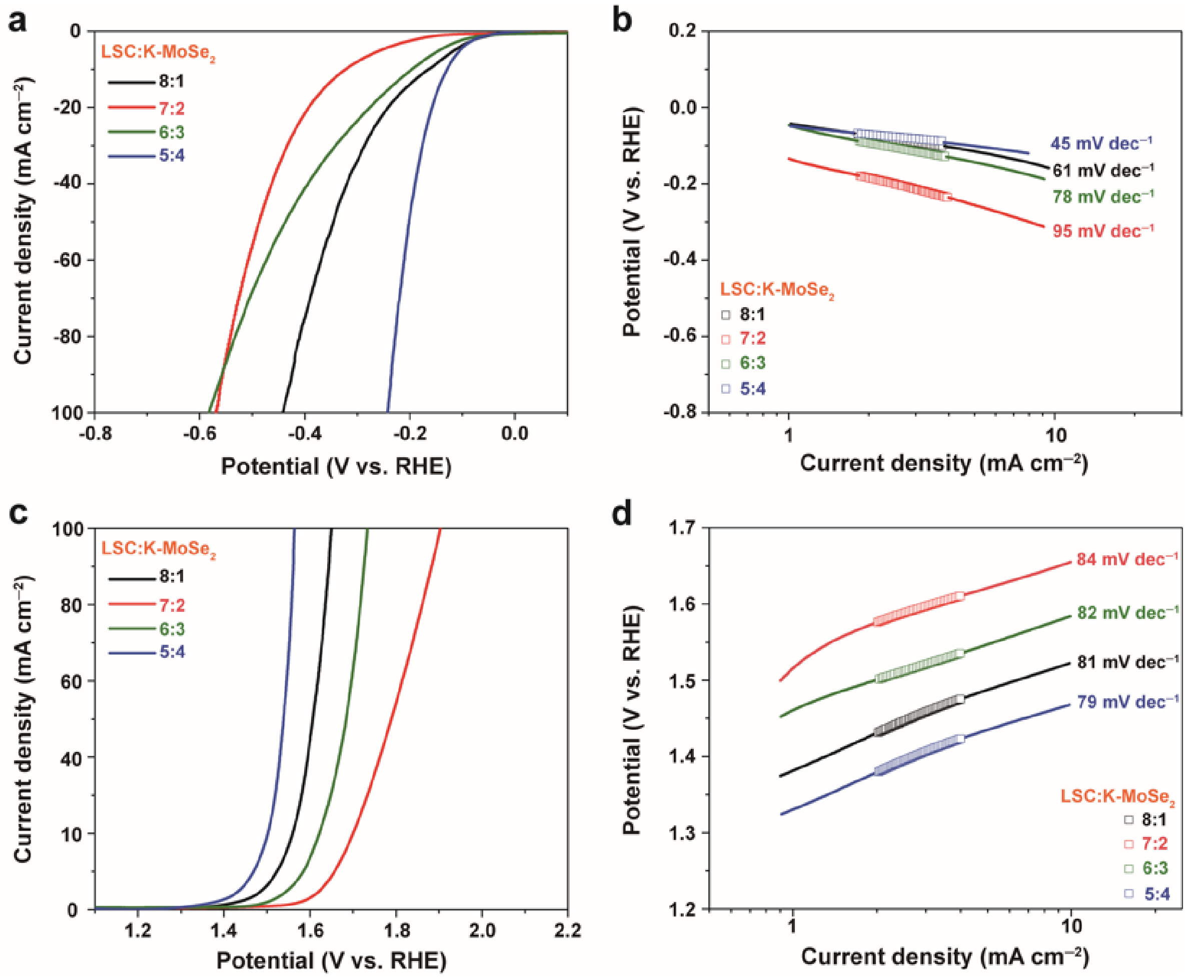

Supplementary Figure 4 a, c HER and OER polarization curves of LSC/K-$MoSe_2$ with various weight ratios and **b, d** corresponding Tafel slopes.

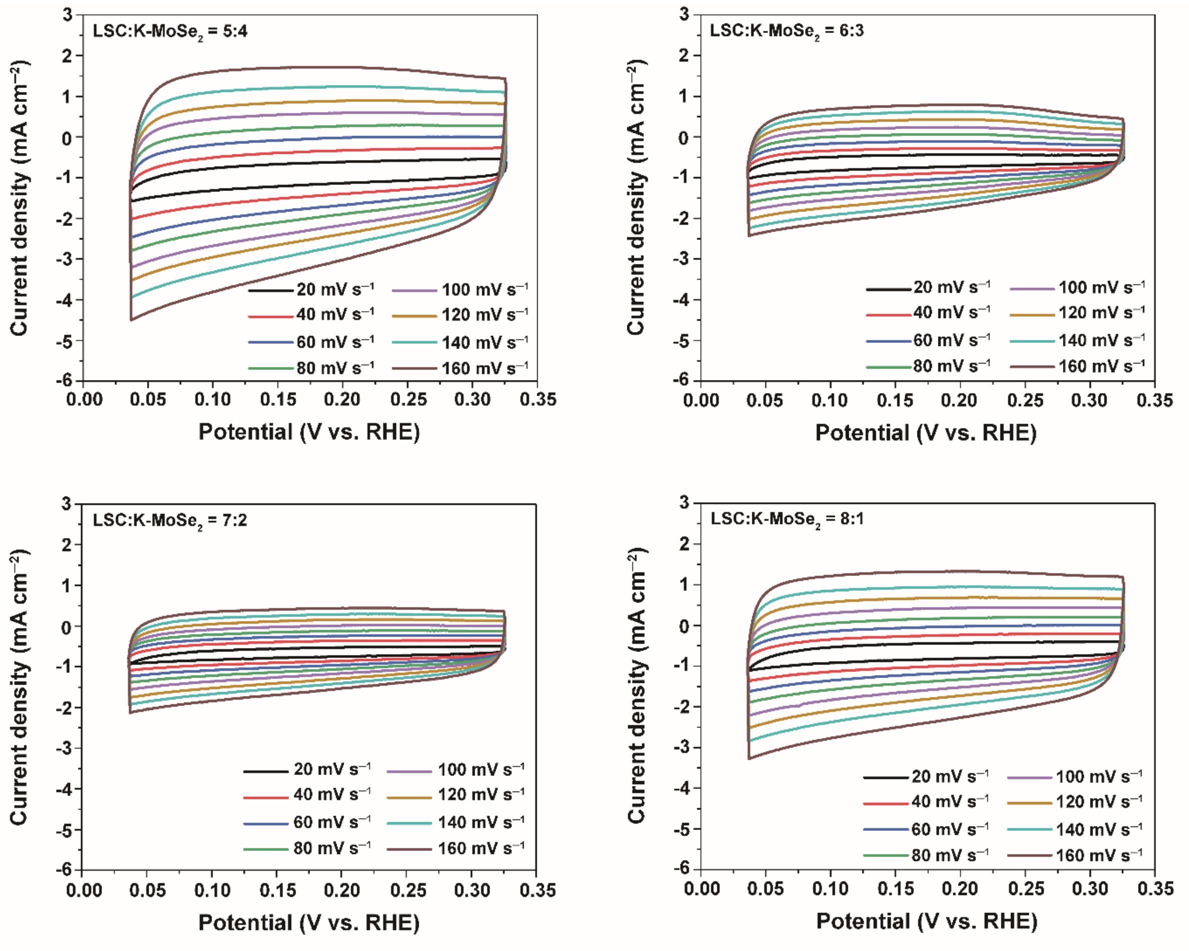

Supplementary Figure 5 Cyclic voltammetry (CV) curve of various weight ratios of LSC/K-$MoSe_2$. Each CV is measured in the double-layer capacitance region at scan rates of 20, 40, 60, 80, 100, 120, 140, and 160 mV s^{-1} in 1 M KOH.

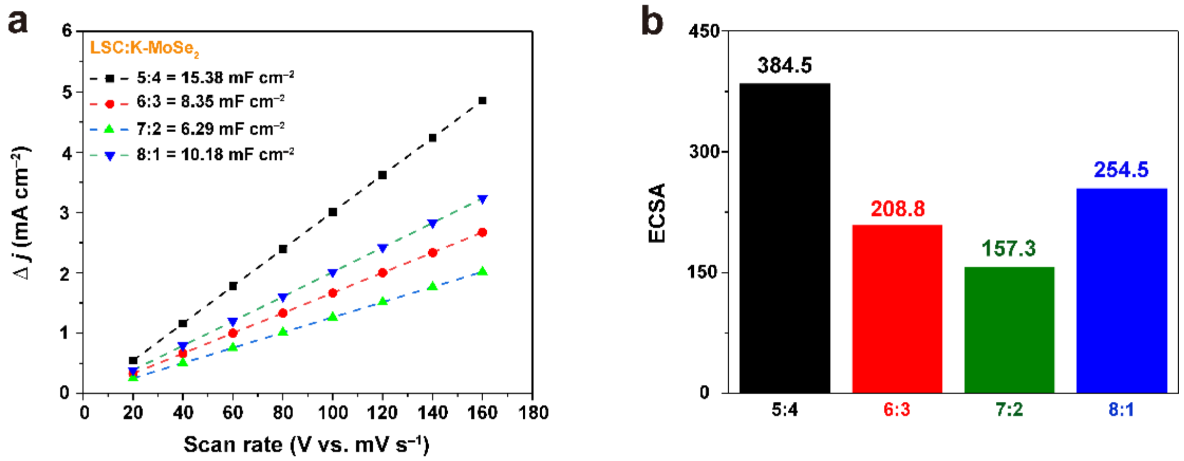

Supplementary Figure 6 Double-layer capacitance values (C_{dl}) of LSC/K-$MoSe_2$ with various weight configurations. Charging current density difference ($\Delta j = (j_a - j_c)/2$) at 0.18 V is plotted against the scan rate for LSC/K-$MoSe_2$. **b** ECSA values of LSC/K-$MoSe_2$ with various weight ratios.

The ECSA values are calculated from C_{dl} according to the following formular.

$$\mathrm{ECSA} = \frac{C_{dl}}{C_s}$$

, where C_s is the specific capacitance of a flat surface with 1 cm^2 of real surface area and C_{dl} is electrochemical double-layer capacitance measured by CV analysis in the non-Faradaic region at different scan rates of 20, 40, 60, 80, 100, 120, 140, and 160 mV s^{-1}. Commonly used C_s value of 0.040 mF cm^{-2} in alkaline solution from literatures was adopted[1,2], and the calculated ECSA values with different wt% of LSC/K-$MoSe_2$ were found to be 384.5 (5:4), 208.8 (6:3), 157.3 (7:2), and 254.5 (8:1), respectively.

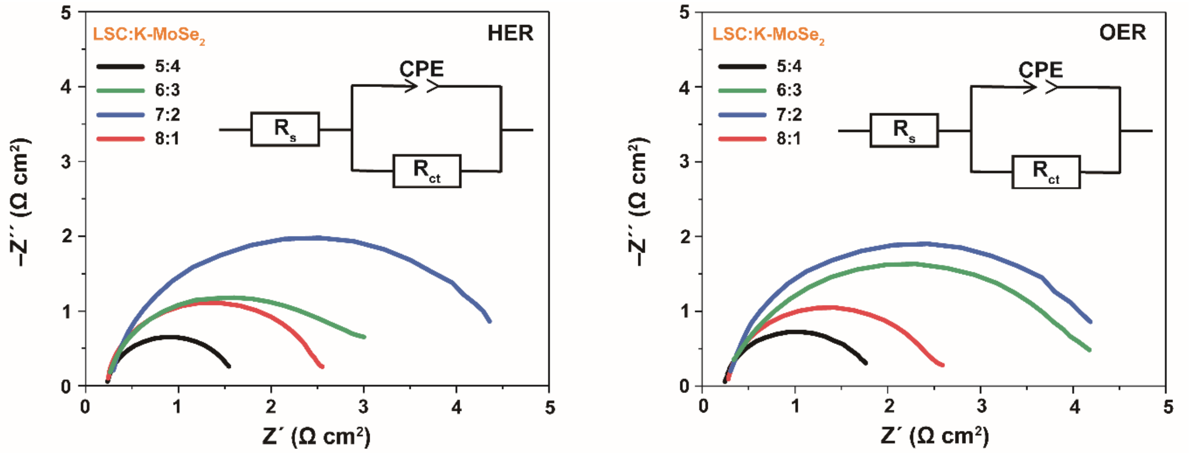

Supplementary Figure 7 Nyquist plots for HER and OER of LSC/K-$MoSe_2$ with various weight configurations.

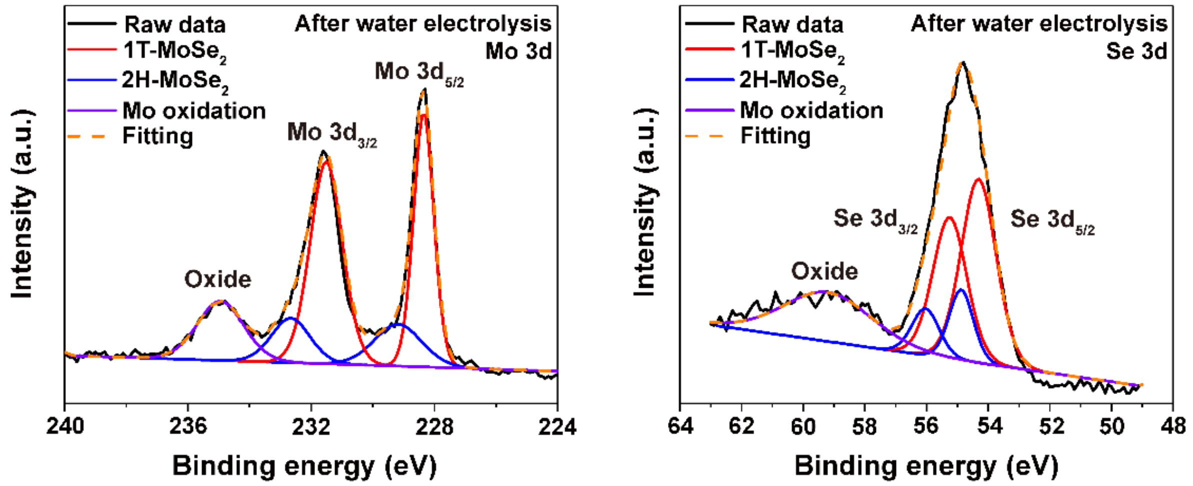

Supplementary Figure 8 High resolution XPS spectra of Mo 3d and Se 3d for LSC/K-$MoSe_2$ in OER condition after water electrolysis.

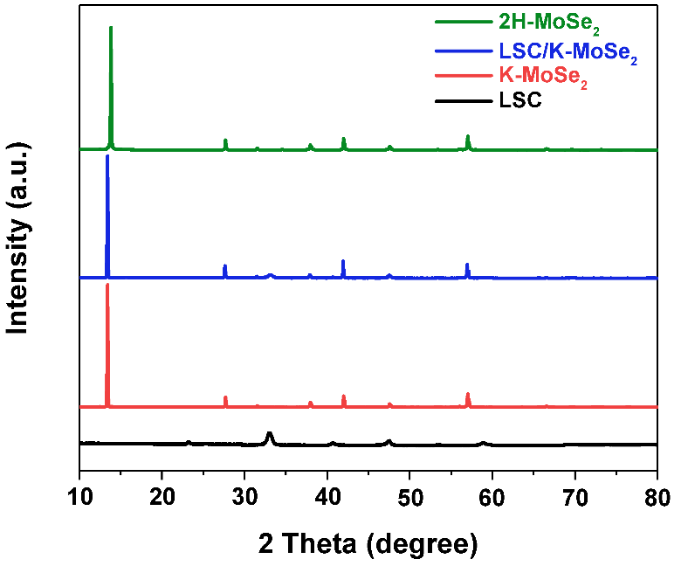

Supplementary Figure 9 XRD patterns for 2H-$MoSe_2$, LSC/K-$MoSe_2$, K-$MoSe_2$, and LSC.

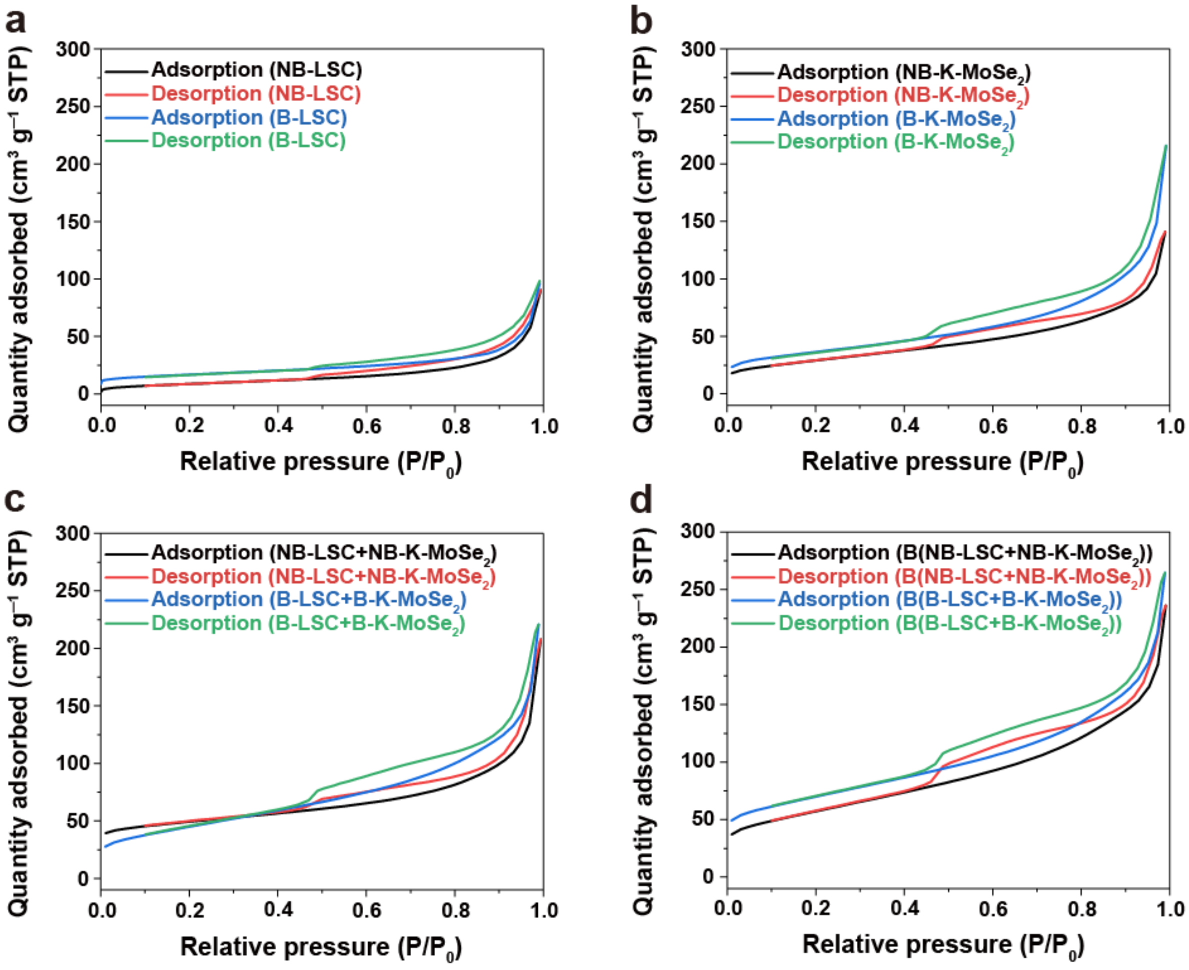

Supplementary Figure 10 BET surface area analysis of various components and combinations of **a** LSC, **b** K-$MoSe_2$, **c** LSC and K-$MoSe_2$ mixed manually, and **d** LSC and K-$MoSe_2$ mixed via ball milling prepared without and with the ball-milling process.

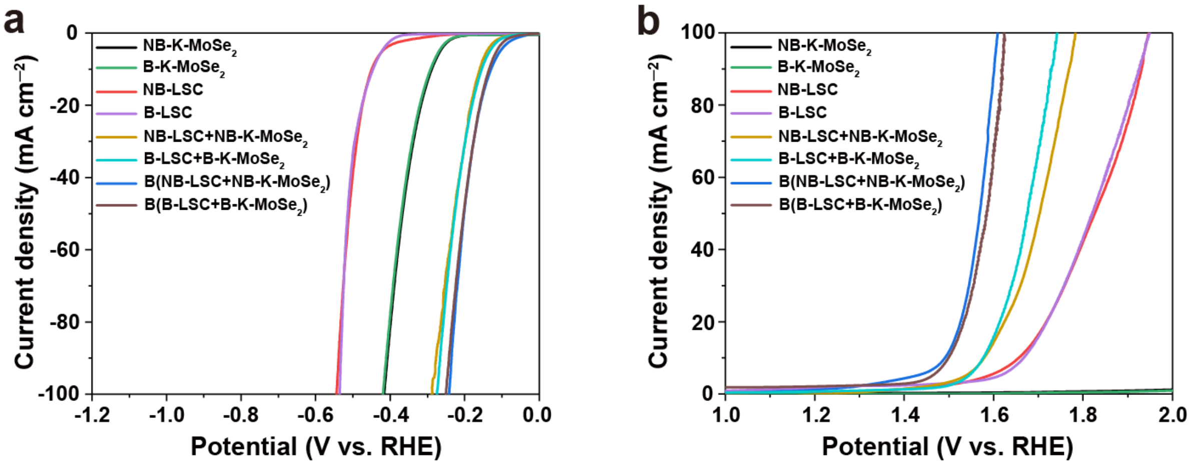

Supplementary Figure 11 a HER and **b** OER polarization profiles of various conditioned LSC and K-$MoSe_2$ combinations.

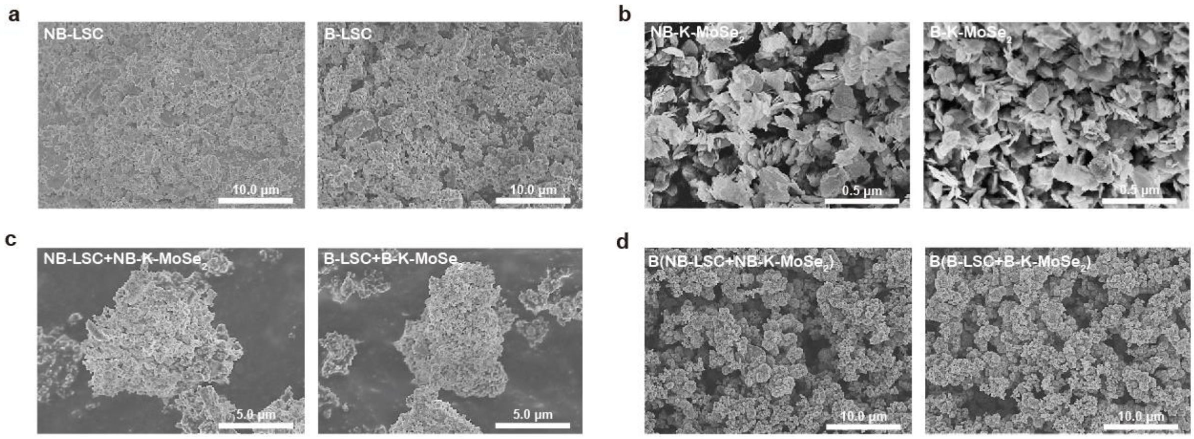

Supplementary Figure 12 SEM images of various components and combinations of **a** LSC, **b** K-$MoSe_2$, **c** LSC and K-$MoSe_2$ mixed manually, and **d** LSC and K-$MoSe_2$ mixed via ball milling prepared without and with the ball-milling process.

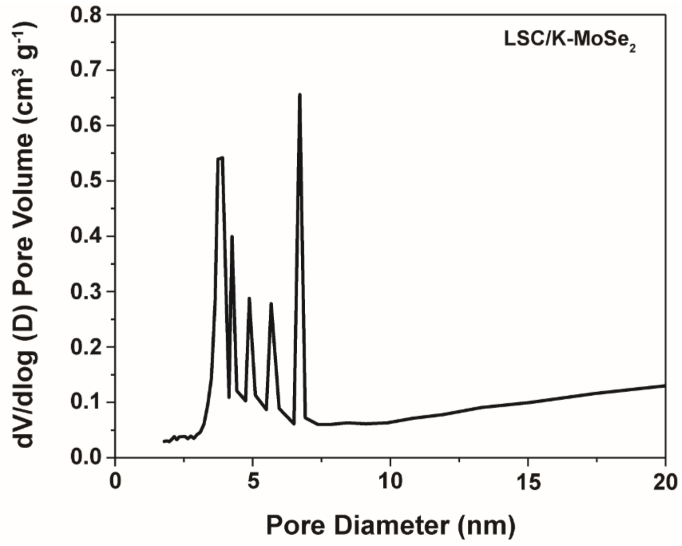

Supplementary Figure 13 Pore size distribution of LSC/K-$MoSe_2$ obtained from Barret–Joyner–Halenda (BJH) calculation.

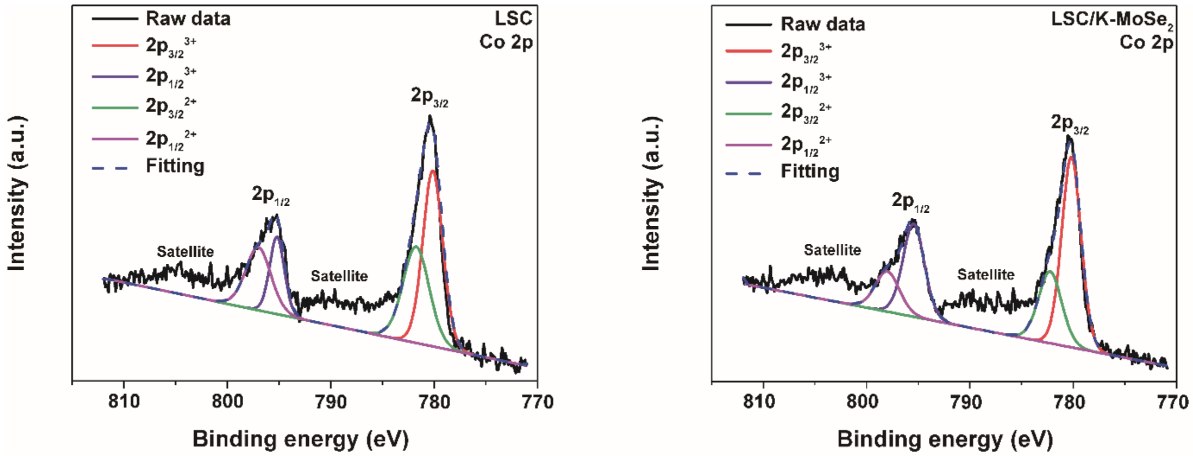

Supplementary Figure 14 High resolution XPS spectra of Co 2p peaks for LSC and LSC/K-$MoSe_2$.

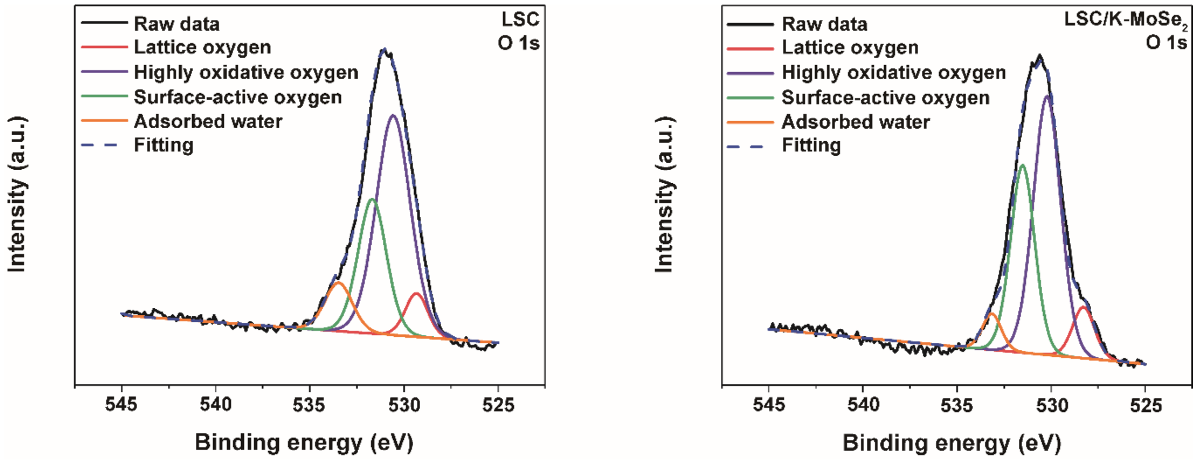

Supplementary Figure 15 High resolution XPS spectra of O 1s peaks for LSC and LSC/K-$MoSe_2$.

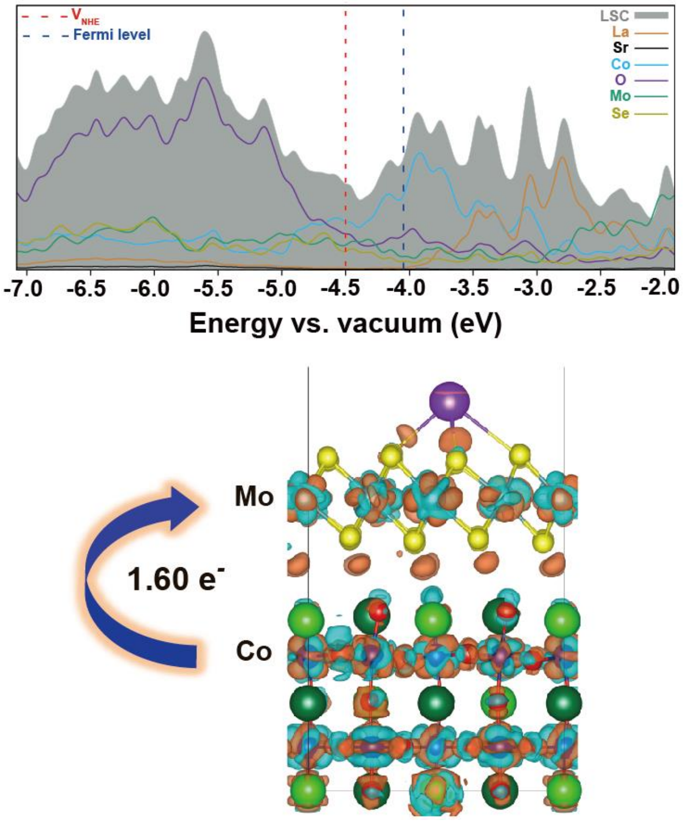

Supplementary Figure 16 Density of states for LSC/K-$MoSe_2$ heterostructure (top) and illustration of electron transfer in LSC/K-$MoSe_2$ (bottom) with charge density difference plot where cyan and brown regions represent electron charge depletion and accumulation, respectively. See **Supplementary Note 2** for detailed discussion on charge transfer in LSC/K-$MoSe_2$.

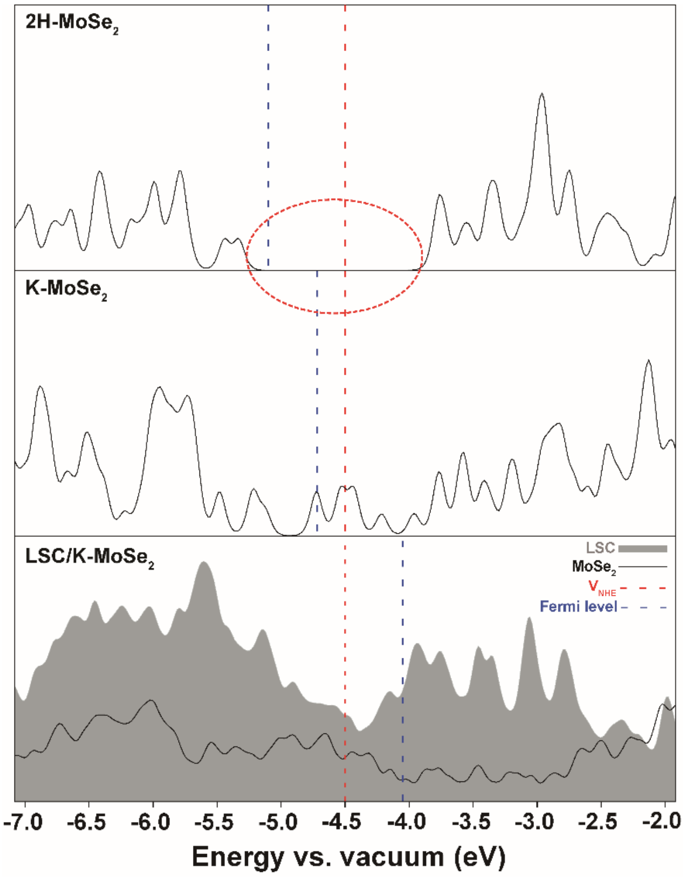

Supplementary Figure 17 Density of states for $MoSe_2$ (2H), K-$MoSe_2$ (1T), and LSC/K-$MoSe_2$ (1T).

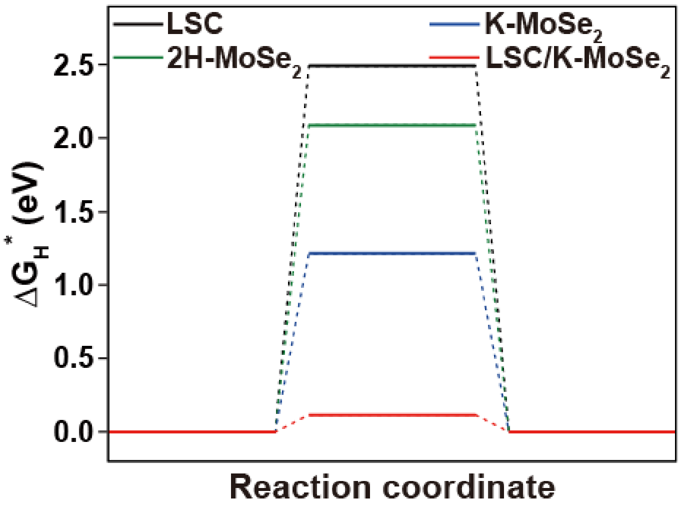

Supplementary Figure 18 Free energy diagrams with the smallest ΔG_{H^*} in the LSC/K-$MoSe_2$ for HER for LSC, 2H-$MoSe_2$, K-$MoSe_2$, and LSC/K-$MoSe_2$.

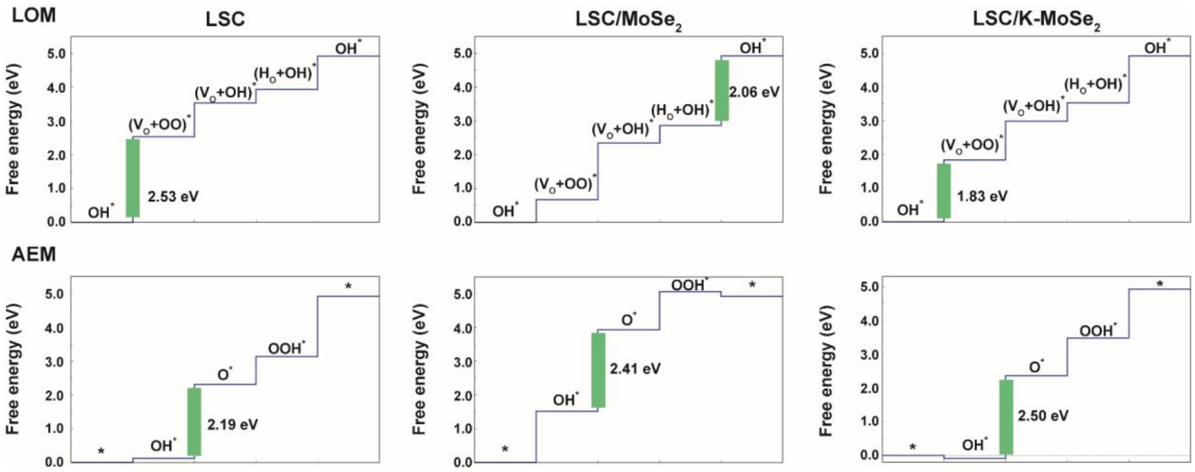

Supplementary Figure 19 Free energy diagrams for OER for LSC (left), LSC/$MoSe_2$ (middle), and LSC/K-$MoSe_2$ (right). The upper and lower rows show the lattice-oxygen participation mechanism (LOM) and adsorbate evolution mechanism (AEM), respectively. All calculations were performed at pH = 0, T = 298 K, and zero applied potential (0 V vs RHE). * indicates that the adsorbates are bound to the LSC (001) surfaces. The green bars indicate the free energy barriers for the rate-determining steps.

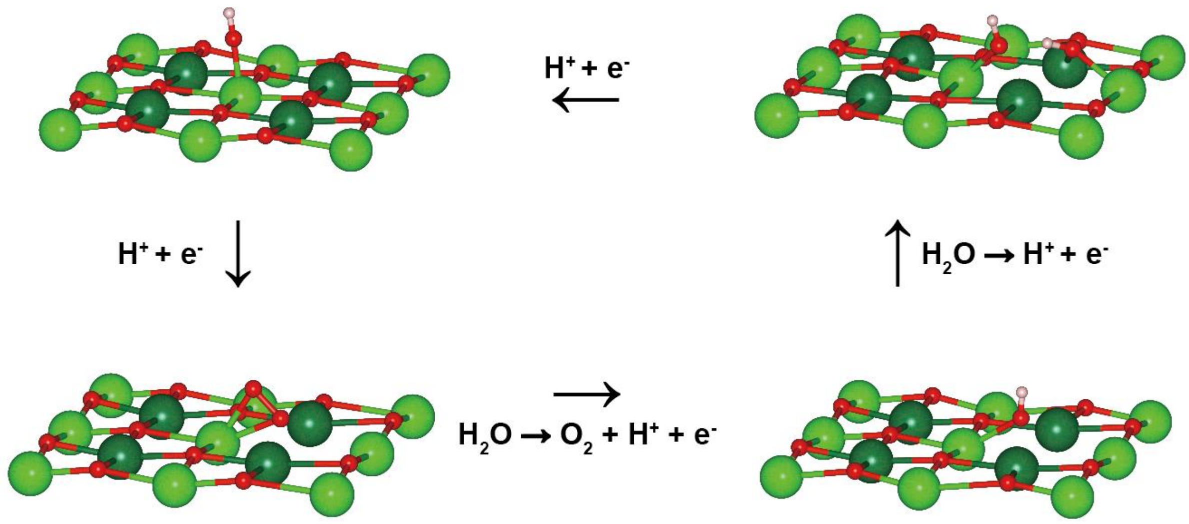

Supplementary Figure 20 Oxygen evolution reaction via the lattice-oxygen participation mechanism on the LSC surface. Red, pink, bright green, and dark green spheres represent oxygen, hydrogen, lanthanum, and strontium atoms, respectively. The top-left, bottom-left, bottom-right, and top-right structures indicate OH*, (V_O+OO)*, (V_O+OH)*, and (H_O+OH)* adsorbed on the LSC (001) surface, respectively.

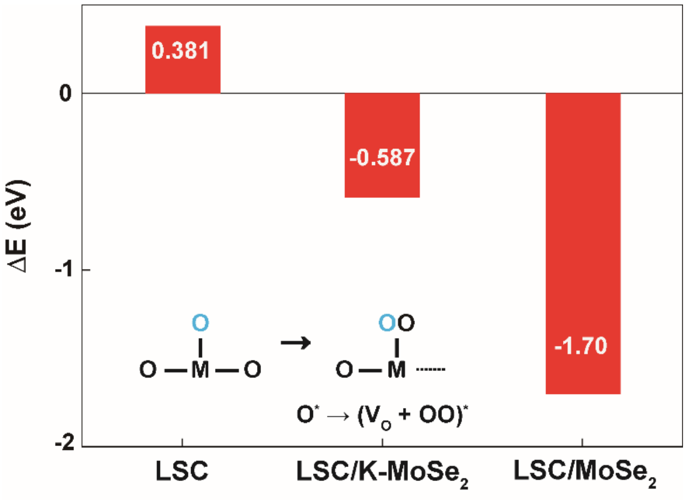

Supplementary Figure 21 Energy differences, ΔE = E((Vo+OO)*) − E(O*), for LSC, LSC/K-$MoSe_2$, and LSC/$MoSe_2$, where E((Vo+OO)*) and E(O*) indicate the total energies of (Vo+OO)* and O* structures, respectively.

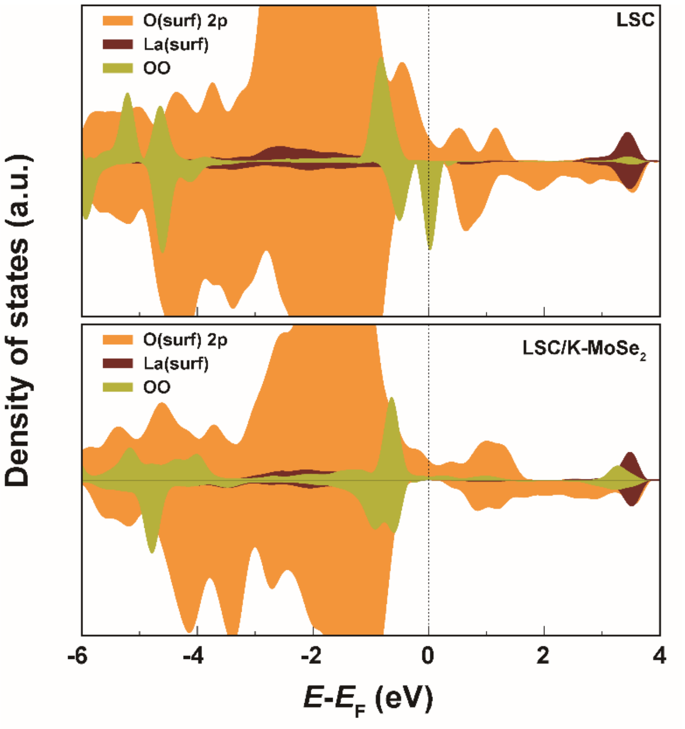

Supplementary Figure 22 Density of states of O 2*p* (surface), La (surface), and adsorbate (OO) states for bare LSC and LSC/K-$MoSe_2$. The dotted lines indicate the Fermi levels.

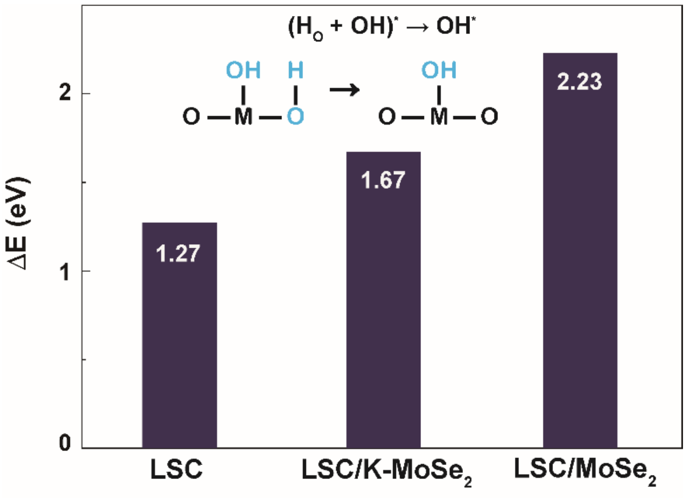

Supplementary Figure 23 Energy differences, ΔE = E(OH*) − E((H$_O$+OH)*), for LSC, LSC/K-MoSe$_2$, and LSC/MoSe$_2$, where E(OH*) and E((H$_O$+OH)*) indicate the total energies of OH* and (H$_O$+OH)* structures, respectively.

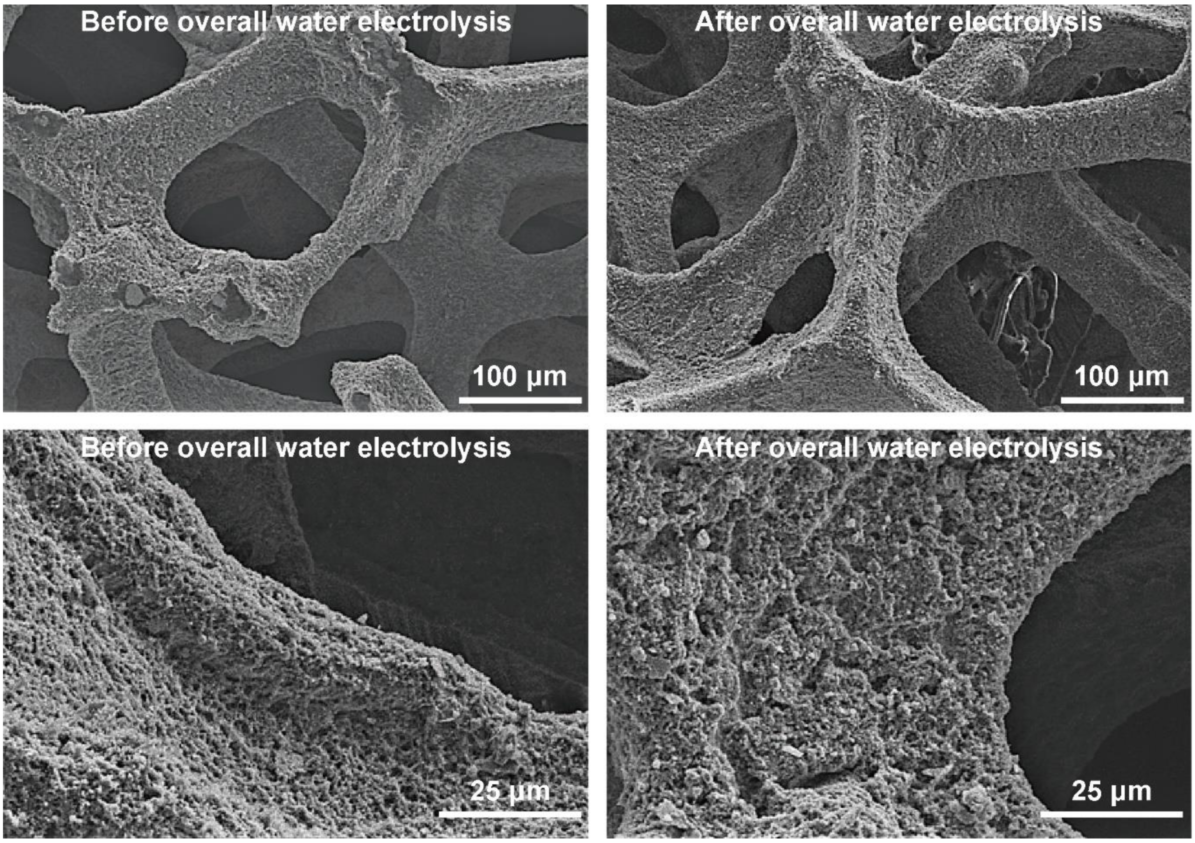

Supplementary Figure 24 SEM images of LSC/K-$MoSe_2$ on Ni foam before and after 2,500 h of overall water electrolysis, illustrating excellent physical integrity of the heterostructure.

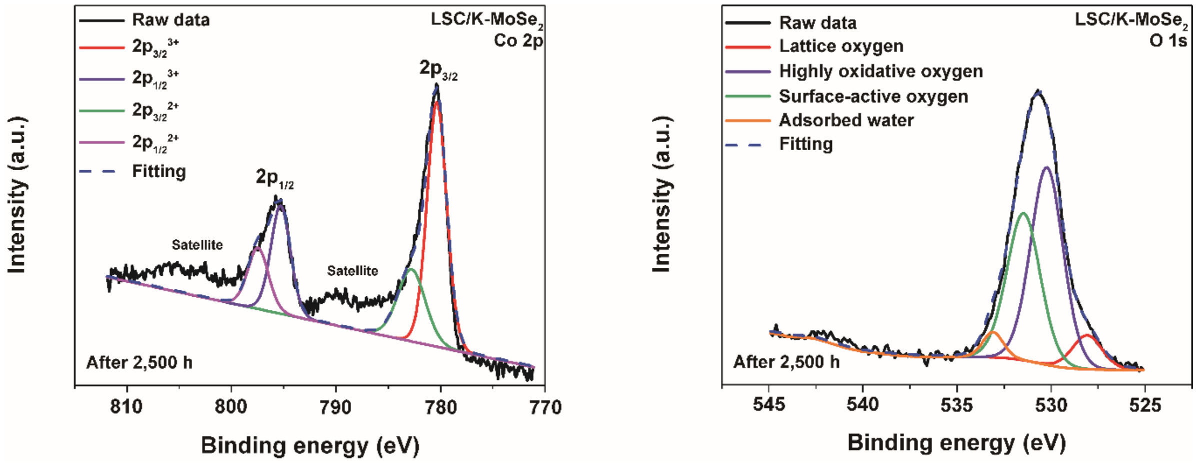

Supplementary Figure 25 XPS core-level spectra of Co 2p and O 1s for LSC/K-$MoSe_2$ after 2,500 h of overall water electrolysis, verifying the excellent chemical durability of LSC/K-$MoSe_2$.

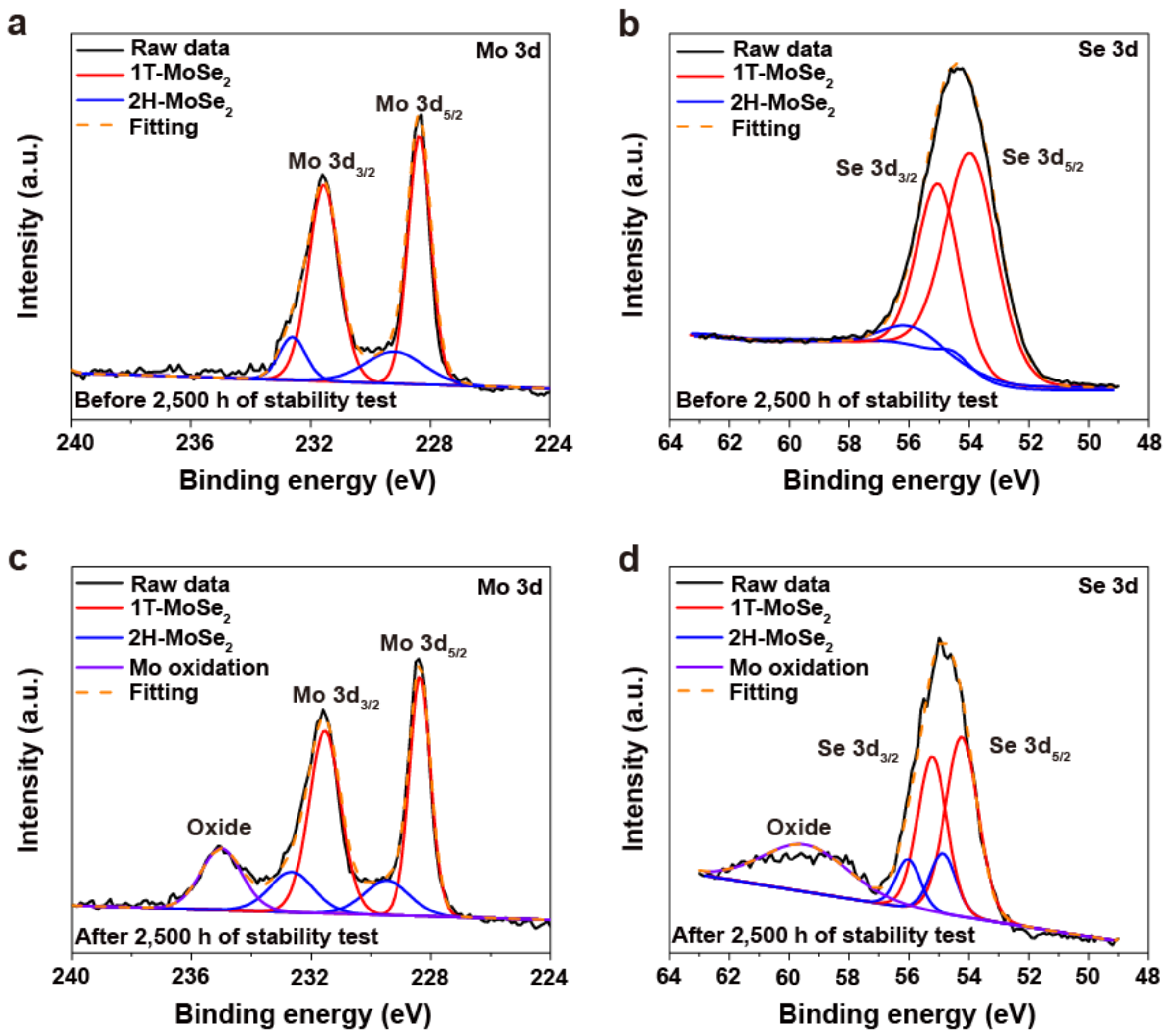

Supplementary Figure 26 High resolution XPS spectra of **a, c** Mo 3d and **b, d** Se 3d for LSC/K-$MoSe_2$ in OER condition before and after 2,500 h of chronopotentiometric stability test.

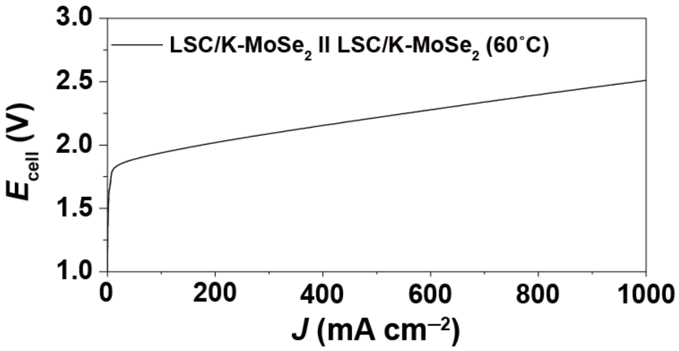

Supplementary Figure 27 Polarization I-V curve of LSC/K-$MoSe_2$ || LSC/K-$MoSe_2$ in 1 M KOH at 60℃.

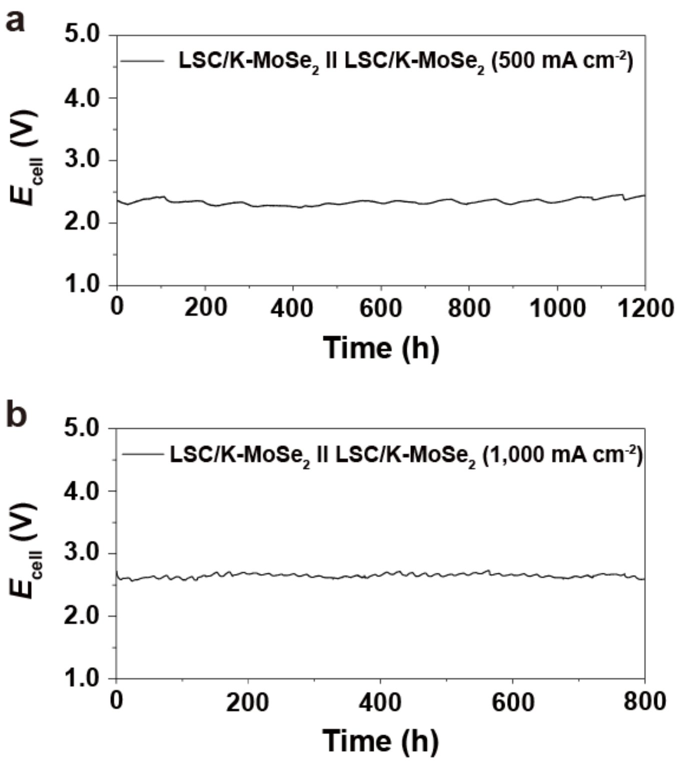

Supplementary Figure 28 Chronopotentiometric stability profiles of LSC/K-$MoSe_2$ || LSC/K-$MoSe_2$ measured at **a** 500 mA cm^{-2} and **b** 1,000 mA cm^{-2} in 1 M KOH at 60°C.

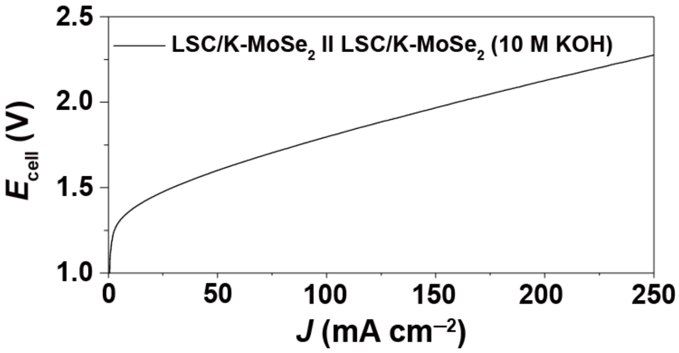

Supplementary Figure 29 Polarization I-V curve of LSC/K-$MoSe_2$ || LSC/K-$MoSe_2$ in 10 M KOH at room temperature.

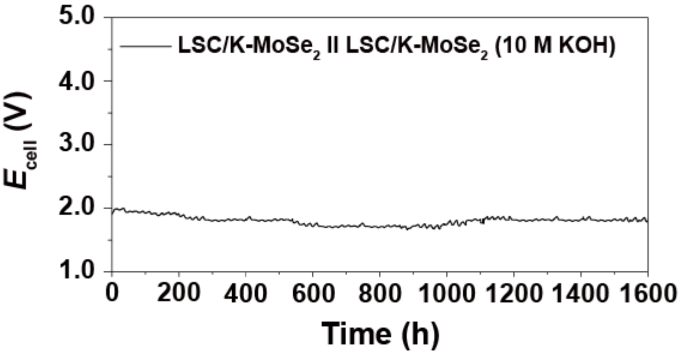

Supplementary Figure 30 Chronopotentiometric stability profiles of LSC/K-$MoSe_2$ || LSC/K-$MoSe_2$ measured at 100 mA cm^{-2} in 10 M KOH at room temperature.

Supplementary Table 1 HER and OER overpotential at 10 mA cm^{-2} of LSC/K-$MoSe_2$ with various weight ratios obtained from the LSV curve in Supplementary Figure 4.

**Overpotential at 10 mA cm^{-2} (V)**	**5:4**	**6:3**	**7:2**	**8:1**
**HER**	-0.128	-0.195	-0.321	-0.165
**OER**	1.46	1.58	1.65	1.52

Supplementary Table 2 Charge transfer resistance of HER and OER for LSC/K-$MoSe_2$ with various weight ratios obtained from Nyquist plots in Supplementary Figure 7.

R_{ct} (Ω cm^2)	5:4	6:3	7:2	8:1
**HER**	1.30	2.73	4.05	2.30
**OER**	1.52	3.83	3.88	2.31

Supplementary Table 3 Quantitative analysis of 1T- and 2H-phase $MoSe_2$ in LSC/K-$MoSe_2$ obtained from the XPS result in Supplementary Figure 8.

**$MoSe_2$ in LSC/K-$MoSe_2$ (atom %)**	
**1T**	Ca. 62.9
**2H**	Ca. 17.9
**Oxide**	Ca. 19.2

Supplementary Table 4 Summary of sample designations tested for the BET surface area analysis prepared with different components and combinations of LSC and K-$MoSe_2$ without and with the ball-milling process.

Abbreviation	Components	BET surface area (m^2 g^{-1})
NB-LSC	Non-ball-milled LSC	32.54
B-LSC	Ball-milled LSC	43.62
NB-K-$MoSe_2$	Non-ball-milled K-$MoSe_2$	104.51
B-K-$MoSe_2$	Ball-milled K-$MoSe_2$	112.62
NB-LSC+NB-K-$MoSe_2$	Non-ball-milled LSC and Non-ball-milled K-$MoSe_2$ via manual mixing	143.47
B-LSC+B-K-$MoSe_2$	Ball-milled LSC and ball-milled K-$MoSe_2$ via manual mixing	162.84
**B(NB-LSC+NB-K-$MoSe_2$)**	**Non-ball-milled LSC and Non-ball-milled K-$MoSe_2$ mixed via ball milling**	**203.94**
B(B-LSC+B-K-$MoSe_2$)	Ball-milled LSC and ball-milled K-$MoSe_2$ mixed via ball milling	221.89

Supplementary Table 5 Quantification of the Co^{3+}/Co^{2+} ratio in LSC and LSC/K-$MoSe_2$ obtained from the XPS analysis shown in Supplementary Figure 14.

	**LSC (atom %)**	**LSC/K-$MoSe_2$ (atom %)**
**Co $2p_{3/2}$, Co^{3+}**	59.2	68.3
**Co $2p_{3/2}$, Co^{2+}**	40.8	31.7
**Co^{3+}/Co^{2+}**	ca. 1.5	ca. 2.2

Supplementary Table 6 Quantification of lattice oxygen (LO), highly oxidative oxygen (OO), surface-active oxygen (SO), and adsorbed water (AW) of LSC and LSC/K-$MoSe_2$ obtained from the XPS analysis in Supplementary Figure 15.

	**LSC (atom %)**	**LSC/K-$MoSe_2$ (atom %)**
**LO**	9.8	9.8
**OO**	49.2	48.6
**SO**	28.8	35.2
**AW**	12.2	6.4
**SO/LO**	ca. 2.9	ca. 3.6

Supplementary Table 7 Quantification of the Co^{3+}/Co^{2+} ratio in LSC/K-$MoSe_2$ obtained from the XPS analysis in Supplementary Figure 19.

	**LSC/K-$MoSe_2$ (atom %)**
**Co $2p_{3/2}$, Co^{3+}**	70.6
**Co $2p_{3/2}$, Co^{2+}**	29.4
**Co^{3+}/Co^{2+}**	ca. 2.4

Supplementary Table 8 Quantification of lattice oxygen (LO), highly oxidative oxygen (OO), surface-active oxygen (SO), and adsorbed water (AW) of LSC/K-$MoSe_2$ obtained from the XPS analysis in Supplementary Figure 19.

	**LSC/K-$MoSe_2$ (atom %)**
**LO**	9.9
**OO**	48.5
**SO**	35.3
**AW**	6.3
**SO/LO**	ca. 3.6

Supplementary Table 9 Quantitative analysis of 1T- and 2H-phase $MoSe_2$ in LSC/K-$MoSe_2$ obtained from the XPS result in Supplementary Figure 25.

$MoSe_2$ in LSC/K-$MoSe_2$ (atom %)	
**1T**	Ca. 62.7
**2H**	Ca. 18.1
**Oxide**	Ca. 19.2

Supplementary Table 10 Comparison of overall water electrolysis chronopotentiometric stability with current density and cell voltage of various reported electrocatalysts in 1 M KOH electrolytes.

Catalyst	Current density (mA cm^{-2})	Cell Voltage (V)	Stability (h)	Electrolyte	Ref.
**This work**	**100**	**1.95**	**2,500**	**1 M KOH**	-
LSC&$MoSe_2$	100	2.30	1,000	1 M KOH	3
NiSe	20	1.75	12	1 M KOH	4
NiS	10	1.61	12	1 M KOH	5
FeCoNi	10	1.68	10	1 M KOH	6
CuFe	10	1.64	100	1 M KOH	7
FeS/NiS	10	1.62	10	1 M KOH	8
Co@GDY-Co	100	1.70	10	1 M KOH	9
FeP/Ni_2P	100	1.61	40	1 M KOH	10
NiFeV-LDH/NF	30	1.59	15	1 M KOH	11
NiS	13	1.88	35	1 M KOH	12
NiFe LDH@NiCoP	10	1.57	100	1 M KOH	13
CoMnCH	10	1.68	13	1 M KOH	14
MoO_2	10	1.53	24	1 M KOH	15
Cu@NiFe LDH	100	1.69	24	1 M KOH	16
Cu@NiFe LDH	10	1.54	48	1 M KOH	16
Cr-doped FeNi-P/NCN	10	1.50	20	1 M KOH	17
$(Co_{1-x}Ni_x)(S_{1-y}P_y)$	20	1.55	100	1 M KOH	18
$(Co_{1-x}Ni_x)(S_{1-y}P_y)$	10	1.52	100	1 M KOH	18
$NiCo_2S_4$/NF	10	1.61	70	1 M KOH	19
Cp@Ni-P	10	1.63	100	1 M KOH	20
Cp@Ni-P	20	1.73	100	1 M KOH	20

$Co(OH)_2$@NCNT@NF	10	1.72	600	1 M KOH	21
$NiCo_2S_4$NW/NF	10	1.63	50	1 M KOH	22
$Co_5Mo_{1.0}O$ NSs@NF	10	1.68	30	1 M KOH	23

Supplementary Note 1

Energy efficiency calculation

The energy efficiency of overall water electrolysis for the bifunctional LSC/K-$MoSe_2$ catalyst was calculated as follows. The LSC/K-$MoSe_2$ catalyst exhibits a cell voltage of 1.95 V at 100 mA cm^{-2} during water electrolysis. For a current density of 100 mA cm^{-2} (0.1 C s^{-1}), the amount of transferred electrons can be obtained as 1.036×10^{-6} mol s^{-1} cm^{-2} using the Faraday constant (F = 96,500 C mol^{-1} e^{-}). Then, the hydrogen generation rate was calculated as 5.181×10^{-7} mol H_2 s^{-1} cm^{-2} (equivalent to 3.731×10^{-3} g H_2 h^{-1} cm^{-2}). As the multiplication constant for generating 1 kg of H_2 can be determined as 2.681×10^{5} h cm^{2}, the power density of LSC/K-$MoSe_2$ catalyst is 0.195 W cm^{-2}. Therefore, the bifunctional LSC/K-$MoSe_2$ catalyst requires 52.28 kWh kg^{-1} of energy for producing 1 kg of H_2. Because the theoretical minimum energy required for generating 1 kg of H_2 is 39.4 kWh kg^{-1}, the energy efficiency of LSC/K-$MoSe_2$ catalyst is 75.36%, which is better than those of the noble-metal-based catalysts (less than 70%)[24].

Supplementary Note 2

HER simulations

The density of states (DOS) of catalysts can provide valuable information such as hydrogen-catalyst charge transfers, bond strength, and electrical conductivity. As shown in **Supplementary Figure 12**, the larger number of states for Co near the Fermi level than those for La and Sr reveals that the charge transfer from LSC to $MoSe_2$ mainly occurs in the CoO_2 layer. The DOS for LSC/K-$MoSe_2$ and K-$MoSe_2$ (**Supplementary Figure 13**) clearly show the states near the normal hydrogen electrode (NHE) energy while no state is observed in 2H-$MoSe_2$, indicating that the charge-transfer-induced 1T-$MoSe_2$ can provide strong interactions between a hydrogen *s* orbital and the 1T-$MoSe_2$ states. Furthermore, the large number of states near the Fermi level in LSC/K-$MoSe_2$ can improve the electrical conductivity of the catalyst, enhancing the overall catalytic activity in LSC/K-$MoSe_2$ heterostructure with increased charge transfer kinetics.

The smallest ΔG_{H^*} in the LSC/K-$MoSe_2$ and the order of ΔG_{H^*} values (LSC/K-$MoSe_2$ < K-$MoSe_2$ < 2H-$MoSe_2$ < LSC) was also confirmed with a stricter force convergence criterion (5×10^{-3} eV/Å) where ΔG_{H^*} values for LSC, 2H-$MoSe_2$, K-$MoSe_2$, and LSC/K-$MoSe_2$ were 2.49, 2.09, 1.22, and 0.11 eV, respectively (**Supplementary Figure 18**).

Supplementary Note 3

OER simulations

The comparison between LOM and AEM for OER free energies are shown in **Supplementary Figure 19**. The LOM proceeds with elementary reactions below:

$$OH^{*} \rightarrow (V_O\text{+}OO)^{*} + H^{+} + e^{-}$$

$$(V_O\text{+}OO)^{*} + H_2O_{(l)} \rightarrow (V_O\text{+}OH)^{*} + O_{2(g)} + H^{+} + e^{-}$$

$$(V_O\text{+}OH)^{*} + H_2O_{(l)} \rightarrow (H_O\text{+}OH)^{*} + H^{+} + e^{-}$$

$$(H_O\text{+}OH)^{*} \rightarrow OH^{*} + H^{+} + e^{-}$$

, where OH, V_O, OO, and H_O indicate OH adsorbates, oxygen vacancies, OO adsorbates, and hydrogen atoms adsorbed on lattice oxygen, respectively. * represents that the adsorbates are bound to the LSC (001) surface. The examples of detailed atomic structures for LOM on the LSC (001) surface are illustrated in **Supplementary Figure 20**. The AEM proceeds with elementary reactions below:

$$^{*} + H_2O_{(l)} \rightarrow OH^{*} + H^{+} + e^{-}$$

$$OH^{*} \rightarrow O^{*} + H^{+} + e^{-}$$

$$O^{*} + H_2O_{(l)} \rightarrow OOH^{*} + H^{+} + e^{-}$$

$$OOH^{*} \rightarrow ^{*} + O_{2(g)} + H^{+} + e^{-}$$

, where OOH indicates OOH adsorbates.

As shown in **Supplementary Figure 19**, while the AEM shows a larger free energy barrier in LSC/K-$MoSe_2$ than in LSC and LSC/$MoSe_2$, the LOM explains that the experimentally measured OER catalytic performance of LSC/K-$MoSe_2$ outperformed LSC and LSC/$MoSe_2$ with reduced free energy barriers, indicating that LOM is the dominant OER path in LSC/K-$MoSe_2$.

The LOM simulation results for LSC, LSC/K-$MoSe_2$, and LSC/$MoSe_2$ show that the rate-determining steps differ from each other; OH* to $(V_O+OO)^*$ for LSC and LSC/K-$MoSe_2$, and $(H_O+OH)^*$ to OH* for LSC/$MoSe_2$. The free energy barriers of these two reactions can be understood using the structural energy differences and density of states of the absorbates shown in **Supplementary Figure 21-23**.

The OH* to $(V_O+OO)^*$ reaction includes the detachment of hydrogen atom from the OH adsorbate ($OH^* \rightarrow O^* + H^+ + e^-$), and the participation of the lattice oxygen, forming OO adsorbates ($O^* \rightarrow (V_O+OO)^* + H^+ + e^-$); the latter is illustrated in **Supplementary Figure 21**. The energy difference between O* and $(V_O+OO)^*$ can be used to evaluate how easily the lattice oxygen can participate[25] and the decreasing order of the energy differences for LSC > LSC/K-$MoSe_2$ > LSC/$MoSe_2$, as shown in **Supplementary Figure 21**, indicate that $(V_O+OO)^*$ structures are energetically favorable in the order of LSC < LSC/K-$MoSe_2$ < LSC/$MoSe_2$, leading to the free energy barriers in the order of LSC > LSC/K-$MoSe_2$ > LSC/$MoSe_2$. The large free energy barriers in OH* to $(V_O+OO)^*$ reaction were also confirmed from the density of states shown in **Supplementary Figure 22**, wherein the state for OO adsorbate in LSC is half-filled, leading to the weak interaction between OO adsorbate and LSC with insufficient charge transfer. In contrast, the $(H_O+OH)^*$ to OH* reaction includes a simple detachment of a hydrogen atom from the lattice oxygen in LSC. The hydrogen desorption energies shown in **Supplementary Figure 23**, in the order of LSC < LSC/K-$MoSe_2$ < LSC/$MoSe_2$, are directly related to the free energy barriers in the $(H_O+OH)^*$ to OH* reaction. Although the trend of OER performance and its origins are well-explained with the detailed analysis on the free energy barriers of elementary reactions, there exists a discrepancy in the overpotentials between experiments and computations in an absolute scale. We speculate that the discrepancy is due to the simplified model structures. For example, the model structure used for simulating LSC/K-$MoSe_2$ only qualitatively represents its charge transfer aspect, not replicating all the

possible LSC/K-$MoSe_2$ heterostructures found in the experiment, which is inevitable in the finite-size structure considered in computations. In fact, such discrepancies in an absolute value between computations and experiments are rather commonly observed in previous OER reports[26-30] as well because the finite-size model structure should be used to obtain the meaningful simulation results within a feasible computation time. Furthermore, another possible origin of the discrepancy between experiment and computation in an absolute scale may lie in the difficulty of describing an exact solvation effect especially for the complicated systems. The solvation corrected free energy barriers in the rate determining step for OER in LSC/K-$MoSe_2$ (2.13 eV) obtained using VASPsol code showed an increased discrepancy from the experimentally measured value (1.46 eV @ 10 mA cm^{-2}) than the non-corrected result, due to the lack of information on the inhomogeneous permittivity near the interfaces. The simulation of exact solvation effect is known to be extremely challenging even in the “implicit + explicit” solvation model since the computed interface properties are sensitive to the simulation parameters and the structure of water molecules[31-33]. In addition, the assumption in the detailed OER process should be one of the reasons for the discrepancy between experiment and simulation. Although we evaluated the free energy barriers based on the LOM elementary reactions as described above, there can be alternative elementary reaction paths which are more favorable, and each of the elementary reaction can be divided into unknown fundamental reactions that may affect the simulation results. While clarifying the most suitable elementary reactions pathway is certainly worthwhile and considered as a successful approach to achieve an agreed result with experiments[34], it might be beyond the scope of this study.

Supplementary Note 4

Structures for the intermediates in HER in a POSCAR style.

```
HER_LSC/K-MoSe2_bare
1.0
        11.1999998093         0.0000000000         0.0000000000
         0.0000000000        16.5699996948         0.0000000000
         0.0000000000         0.0000000000        35.0000000000
    K    Mo    Se    La    Sr    Co     O
    1    20    40    18    18    24    84
Direct
     0.505343301         0.405558298         0.485911233
     0.257947854         0.506541619         0.366333035
     0.732206715         0.913893452         0.365910585
     0.245274310         0.112422520         0.366511890
     0.753281385         0.292278824         0.364014980
     0.261745244         0.894295934         0.363785880
     0.751092702         0.508157570         0.364593097
     0.261116288         0.292056175         0.364355768
     0.476945306         0.393190748         0.364156696
     0.250471771         0.709193731         0.365390015
     0.752655962         0.689280298         0.362922532
     0.754901184         0.098091348         0.363871220
     0.487919586         0.989157695         0.363665962
     0.980324336         0.398431352         0.364259829
     0.480542915         0.816078293         0.365158163
     0.981912119         0.604664924         0.364874513
     0.474899462         0.211371945         0.364281000
     0.975659336         0.009264656         0.365229034
     0.985501511         0.195685398         0.363453729
     0.493352975         0.606576359         0.364293671
     0.991277030         0.785660398         0.363217027
     0.317984250         0.397115662         0.415801403
     0.821629095         0.001659350         0.418555314
     0.325873266         0.803869477         0.418472399
     0.825223596         0.600350943         0.418499647
     0.821001203         0.399724137         0.415153149
     0.321025875         0.000466791         0.414200865
     0.326870792         0.201924842         0.419941820
     0.825069135         0.195186819         0.416876166
     0.822128242         0.801112282         0.410869871
     0.331439552         0.607168017         0.416596385
     0.074151782         0.099988452         0.415791811
     0.578749317         0.299194114         0.413797161
     0.571540136         0.903018342         0.418085126
     0.076424209         0.500890134         0.412189674
     0.078345826         0.698378708         0.416129712
```

0.579550872	0.708065725	0.411176627
0.076725277	0.297081440	0.411897550
0.571576537	0.101352121	0.408689008
0.572976522	0.500128979	0.411337226
0.074498319	0.898511094	0.408806855
0.162251626	0.603917466	0.317819541
0.661179775	0.802651514	0.318898555
0.159431560	0.002683018	0.320099068
0.660015058	0.399931620	0.318599510
0.654291394	0.198187440	0.315750313
0.160189955	0.202959956	0.313863209
0.656151016	0.004359703	0.312595803
0.164048983	0.401746507	0.319351741
0.658193710	0.600524814	0.311899294
0.159725401	0.803286971	0.313323021
0.908504144	0.899643877	0.317961938
0.414075475	0.502093767	0.314821924
0.409364878	0.100686873	0.315922056
0.912926638	0.299923729	0.312108558
0.913320199	0.503289284	0.312894876
0.412234714	0.902164639	0.309075791
0.408935298	0.301642415	0.309554182
0.413506464	0.709445474	0.314185006
0.909575239	0.698537270	0.309761620
0.909624030	0.100406886	0.309887913
0.482641509	0.989738188	0.212849917
0.995257854	0.981697280	0.213647979
0.486970680	0.658723294	0.212849944
0.491125379	0.317600834	0.213225419
0.987690108	0.662814770	0.213022940
0.987016235	0.321136220	0.213099984
0.244313908	0.163817797	0.110063628
0.744313866	0.163817797	0.110063628
0.744313866	0.836182174	0.110063628
0.244313908	0.836182174	0.110063628
0.745519583	0.500000000	0.109964078
0.245519604	0.500000000	0.109964078
0.500000000	0.666666705	0.000000000
0.000000000	0.333333353	0.000000000
0.500000000	0.333333353	0.000000000
0.000000000	0.666666705	0.000000000
0.500000000	0.000000000	0.000000000
0.000000000	0.000000000	0.000000000
0.250223007	0.157136681	0.217704323
0.749840238	0.157324222	0.217531041
0.745284144	0.827183908	0.217565196
0.253616001	0.826303040	0.217355306
0.747560616	0.492934349	0.218047810
0.250168255	0.491738487	0.217648983

0.499390585	-0.000000000	0.109726483
0.999390585	-0.000000000	0.109726483
0.499973817	0.666908260	0.109208918
0.999973859	0.333091740	0.109208918
0.999973859	0.666908260	0.109208918
0.499973817	0.333091740	0.109208918
0.749999979	0.833333295	0.000000000
0.250000000	0.166666676	0.000000000
0.749999979	0.166666676	0.000000000
0.250000000	0.500000000	0.000000000
0.749999979	0.500000000	0.000000000
0.250000000	0.833333295	0.000000000
0.749145844	0.997565109	0.166795077
0.248972799	0.996788471	0.166203131
0.499299518	0.497073308	0.166690050
0.999103035	0.498338466	0.167496681
0.500871888	0.163503738	0.165980148
0.498598610	0.831140765	0.165293271
0.998544965	0.830316242	0.165426050
0.999989271	0.162566121	0.166515664
0.249117511	0.664524880	0.165616608
0.748574065	0.331285802	0.166000203
0.249398185	0.329660958	0.166064780
0.748706388	0.664820191	0.166392149
0.749999979	0.333333353	0.055084293
0.500000000	0.166666676	0.055084293
0.250000000	-0.000000000	0.055084293
-0.000000000	0.166666676	0.055084293
0.250000000	0.333333353	0.055084293
0.000000000	0.500000000	0.055084293
0.250000000	0.666666705	0.055084293
0.749999979	0.666666705	0.055084293
0.000000000	0.833333295	0.055084293
0.500000000	0.833333295	0.055084293
0.749999979	-0.000000000	0.055084293
0.500000000	0.500000000	0.055084293
0.270259555	0.995869560	0.220831667
0.777738022	0.996802400	0.219529057
0.989267332	0.175593531	0.220271887
0.488693850	0.838592605	0.219602271
0.490393775	0.171424300	0.220247732
0.985757725	0.833727657	0.220340429
0.490697077	0.509405807	0.220652049
0.989702701	0.509178123	0.220419312
0.276553039	0.329470971	0.219588716
0.771380932	0.331633717	0.219869396
0.770463190	0.668178483	0.219677026
0.272525256	0.666705094	0.219504997
0.638411865	0.902374712	0.167501095

0.106661258	0.065343793	0.171655914
0.601730953	0.069125180	0.170952170
0.137936582	0.900159563	0.168870081
0.148192806	0.567681180	0.167205933
0.646821016	0.567646705	0.167178753
0.101709921	0.399166090	0.171295765
0.601599354	0.399262321	0.170582771
0.603119912	0.735925160	0.169811126
0.643128806	0.233232742	0.167585959
0.148811268	0.232354694	0.166782420
0.100842131	0.735736036	0.169539765
0.363001532	0.093790187	0.162900993
0.389191643	0.924454892	0.161666965
0.855397597	0.096526282	0.161109298
0.896288001	0.923792788	0.161747306
0.852706702	0.432176201	0.162364592
0.355234408	0.429734777	0.162222794
0.895453366	0.596531238	0.162460136
0.396215539	0.594230046	0.161882305
0.893778220	0.261748986	0.161612756
0.397874422	0.256065501	0.161455413
0.358951055	0.763554147	0.161667333
0.857148866	0.764920888	0.161589568
0.742256739	0.334457359	0.111706379
0.742256739	0.665542670	0.111706379
0.242256718	0.665542670	0.111706379
0.242256718	0.334457359	0.111706379
0.520249486	0.500000000	0.111441694
0.020249490	0.500000000	0.111441694
0.741052725	-0.000000000	0.111310850
0.241052683	-0.000000000	0.111310850
0.520620227	0.834036263	0.111073126
0.020620227	0.165963723	0.111073126
0.020620227	0.834036263	0.111073126
0.520620227	0.165963723	0.111073126
0.374999989	0.583333295	0.055134330
0.374999989	0.916666705	0.055134330
0.625000011	0.583333295	0.055134330
0.875000032	0.583333295	0.055134330
0.374999989	0.416666647	0.055134330
0.875000032	0.416666647	0.055134330
0.625000011	0.916666705	0.055134330
0.374999989	0.250000000	0.055134330
0.875000032	0.916666705	0.055134330
0.125000000	0.750000000	0.055134330
0.625000011	0.750000000	0.055134330
0.625000011	0.250000000	0.055134330
0.125000000	0.250000000	0.055134330
0.125000000	0.583333295	0.055134330

0.875000032	0.083333338	0.055134330
0.625000011	0.083333338	0.055134330
0.374999989	0.083333338	0.055134330
0.374999989	0.750000000	0.055134330
0.875000032	0.250000000	0.055134330
0.125000000	0.083333338	0.055134330
0.125000000	0.416666647	0.055134330
0.875000032	0.750000000	0.055134330
0.625000011	0.416666647	0.055134330
0.125000000	0.916666705	0.055134330
0.500000000	0.500000000	0.000000000
0.250000000	0.666666705	0.000000000
0.000000000	0.833333295	0.000000000
0.749999979	0.666666705	0.000000000
0.500000000	0.833333295	0.000000000
0.000000000	0.500000000	0.000000000
0.250000000	0.000000000	0.000000000
0.749999979	0.333333353	0.000000000
0.250000000	0.333333353	0.000000000
0.500000000	0.166666676	0.000000000
0.000000000	0.166666676	0.000000000
0.749999979	0.000000000	0.000000000

HER_LSC/K-MoSe2_H
1.0

11.1999998093	0.0000000000	0.0000000000
0.0000000000	16.5699996948	0.0000000000
0.0000000000	0.0000000000	35.0000000000

H	K	Mo	Se	La	Sr	Co	O
1	1	20	40	18	18	24	84

Direct

0.084431822	0.892098800	0.441589791
0.474487594	0.410917004	0.485184097
0.249869423	0.507581164	0.364129966
0.734268354	0.915823880	0.364916365
0.231182962	0.116692328	0.365423203
0.738132864	0.318987978	0.364848954
0.275318053	0.893617829	0.362154743
0.749874213	0.502118112	0.363447326
0.239485528	0.282203516	0.363946860
0.495770114	0.402920384	0.363765853
0.254694079	0.706623588	0.363758387
0.758284173	0.689250715	0.361827578
0.753278234	0.089284321	0.362277031
0.503306142	0.993416942	0.362797601
0.976396220	0.411936175	0.364945630
0.485981030	0.816643303	0.363649477
0.981784224	0.601226977	0.362858636
0.471679321	0.206374637	0.365895544

0.980209555	0.022149288	0.365172114
0.000091411	0.189313363	0.362101228
0.487996859	0.596595181	0.362628256
0.994919385	0.781240575	0.360669354
0.316605637	0.395489926	0.411281013
0.826383077	0.002218028	0.417549896
0.329890576	0.802660492	0.417120661
0.823765667	0.600359116	0.416563797
0.821624583	0.406238388	0.418225888
0.325781219	0.003749980	0.409977858
0.319040870	0.200024429	0.419863483
0.822478973	0.204247157	0.409113230
0.824460231	0.800420710	0.408743613
0.329911587	0.603544975	0.415427344
0.073608683	0.102704330	0.418190602
0.572668664	0.302373930	0.415601403
0.577649910	0.900277147	0.417414556
0.076989799	0.503998008	0.413739232
0.079644734	0.696649605	0.414519855
0.577994595	0.703447397	0.407758413
0.068105498	0.301477810	0.410634095
0.577898206	0.101534410	0.410664613
0.577574297	0.504741782	0.414326722
0.077714639	0.900686761	0.399278559
0.161164672	0.603980661	0.314009203
0.668613624	0.802790277	0.318131883
0.162440179	0.000738561	0.319933346
0.658139044	0.405282441	0.310740444
0.657408164	0.204093746	0.320669883
0.158893277	0.201175054	0.309268025
0.662162613	0.003861039	0.309638105
0.158685534	0.399236278	0.319351387
0.659175867	0.600017128	0.312228503
0.168138979	0.802271886	0.312267603
0.915856377	0.903382948	0.319200625
0.408349470	0.500234937	0.311541803
0.406568866	0.100288957	0.316862842
0.911033510	0.301987539	0.317459706
0.911681497	0.502598806	0.310362216
0.420023380	0.904002232	0.308037840
0.406065803	0.303111891	0.313181060
0.422510640	0.706498580	0.313089234
0.911753704	0.696840454	0.307363783
0.909070047	0.104126252	0.309204374
0.487953262	0.986837452	0.213226441
0.992009486	0.984218273	0.214200619
0.490683070	0.651721932	0.213738469
0.485389709	0.325588705	0.213572380
0.989344818	0.654479933	0.212919303

0.987514700	0.325783814	0.213161469
0.244313908	0.163817797	0.110063628
0.744313866	0.163817797	0.110063628
0.744313866	0.836182174	0.110063628
0.244313908	0.836182174	0.110063628
0.745519583	0.500000000	0.109964078
0.245519604	0.500000000	0.109964078
0.500000000	0.666666705	0.000000000
0.000000000	0.333333353	0.000000000
0.500000000	0.333333353	0.000000000
0.000000000	0.666666705	0.000000000
0.500000000	0.000000000	0.000000000
0.000000000	0.000000000	0.000000000
0.250590171	0.161534504	0.217421491
0.749735760	0.159335240	0.218054145
0.751980215	0.824417677	0.217572457
0.247862599	0.822661812	0.217279707
0.750043064	0.491593680	0.217746666
0.246641065	0.492348389	0.217893791
0.499390585	-0.000000000	0.109726483
0.999390585	-0.000000000	0.109726483
0.499973817	0.666908260	0.109208918
0.999973859	0.333091740	0.109208918
0.999973859	0.666908260	0.109208918
0.499973817	0.333091740	0.109208918
0.749999979	0.833333295	0.000000000
0.250000000	0.166666676	0.000000000
0.749999979	0.166666676	0.000000000
0.250000000	0.500000000	0.000000000
0.749999979	0.500000000	0.000000000
0.250000000	0.833333295	0.000000000
0.749752790	0.996762112	0.166944190
0.247632184	0.997972363	0.167350633
0.499240637	0.497238258	0.165317685
0.999673111	0.497827211	0.165702929
0.499714621	0.164666795	0.166785921
0.498069533	0.828841241	0.166179589
0.998909150	0.830195091	0.165218830
0.998623558	0.164135036	0.166817352
0.248599270	0.664662723	0.165506853
0.748465500	0.330122284	0.165723596
0.248546966	0.331320392	0.165637180
0.748283961	0.663129765	0.165809522
0.749999979	0.333333353	0.055084293
0.500000000	0.166666676	0.055084293
0.250000000	-0.000000000	0.055084293
-0.000000000	0.166666676	0.055084293
0.250000000	0.333333353	0.055084293
0.000000000	0.500000000	0.055084293

0.250000000	0.666666705	0.055084293
0.749999979	0.666666705	0.055084293
0.000000000	0.833333295	0.055084293
0.500000000	0.833333295	0.055084293
0.749999979	-0.000000000	0.055084293
0.500000000	0.500000000	0.055084293
0.269860396	0.998145486	0.220253577
0.779577251	0.996562513	0.220492090
0.989740508	0.176470486	0.220384884
0.487029688	0.840807984	0.219936153
0.489004263	0.175773331	0.220489843
0.988714116	0.838206933	0.219851685
0.488907022	0.504536946	0.219487572
0.989398803	0.504383679	0.219463689
0.270909693	0.333320345	0.219599751
0.774040320	0.333499540	0.219309126
0.774405867	0.662897763	0.219233567
0.272854358	0.663605220	0.218697698
0.647974323	0.900703739	0.167667512
0.101407970	0.065898191	0.170714310
0.602296771	0.064814898	0.171599279
0.141026380	0.899921519	0.168031284
0.140245666	0.566536655	0.167564283
0.642523180	0.564546486	0.167813396
0.101196523	0.402589965	0.170222691
0.599765788	0.401540924	0.170353004
0.600101405	0.730156667	0.171278259
0.649138317	0.232489213	0.166902733
0.146675219	0.234053955	0.166591849
0.098360076	0.734746736	0.170130471
0.353100412	0.097372121	0.162056214
0.391169325	0.928679203	0.161755085
0.854708908	0.096214855	0.162316023
0.897896986	0.924037221	0.161882155
0.861232721	0.431840861	0.161807523
0.358130427	0.432599282	0.162197930
0.896977627	0.590863351	0.161535835
0.397911504	0.591020532	0.161834349
0.896168281	0.261244551	0.161692933
0.395830493	0.262638602	0.162070533
0.350429038	0.764618383	0.160655471
0.858030674	0.763843702	0.161511162
0.742256739	0.334457359	0.111706379
0.742256739	0.665542670	0.111706379
0.242256718	0.665542670	0.111706379
0.242256718	0.334457359	0.111706379
0.520249486	0.500000000	0.111441694
0.020249490	0.500000000	0.111441694
0.741052725	-0.000000000	0.111310850

0.241052683	-0.000000000	0.111310850
0.520620227	0.834036263	0.111073126
0.020620227	0.165963723	0.111073126
0.020620227	0.834036263	0.111073126
0.520620227	0.165963723	0.111073126
0.374999989	0.583333295	0.055134330
0.374999989	0.916666705	0.055134330
0.625000011	0.583333295	0.055134330
0.875000032	0.583333295	0.055134330
0.374999989	0.416666647	0.055134330
0.875000032	0.416666647	0.055134330
0.625000011	0.916666705	0.055134330
0.374999989	0.250000000	0.055134330
0.875000032	0.916666705	0.055134330
0.125000000	0.750000000	0.055134330
0.625000011	0.750000000	0.055134330
0.625000011	0.250000000	0.055134330
0.125000000	0.250000000	0.055134330
0.125000000	0.583333295	0.055134330
0.875000032	0.083333338	0.055134330
0.625000011	0.083333338	0.055134330
0.374999989	0.083333338	0.055134330
0.374999989	0.750000000	0.055134330
0.875000032	0.250000000	0.055134330
0.125000000	0.083333338	0.055134330
0.125000000	0.416666647	0.055134330
0.875000032	0.750000000	0.055134330
0.625000011	0.416666647	0.055134330
0.125000000	0.916666705	0.055134330
0.500000000	0.500000000	0.000000000
0.250000000	0.666666705	0.000000000
0.000000000	0.833333295	0.000000000
0.749999979	0.666666705	0.000000000
0.500000000	0.833333295	0.000000000
0.000000000	0.500000000	0.000000000
0.250000000	0.000000000	0.000000000
0.749999979	0.333333353	0.000000000
0.250000000	0.333333353	0.000000000
0.500000000	0.166666676	0.000000000
0.000000000	0.166666676	0.000000000
0.749999979	0.000000000	0.000000000

HER_K-MoSe2_bare
1.0

11.1999998093	0.0000000000	0.0000000000
0.0000000000	16.5699996948	0.0000000000
0.0000000000	0.0000000000	35.0000000000

K	Mo	Se
1	20	40

Direct

0.506745500	0.417782021	0.485709981
0.261285629	0.508441140	0.366105679
0.731391153	0.916539280	0.366481100
0.234787532	0.113814636	0.365944781
0.748643292	0.287417271	0.364051410
0.250963062	0.889525490	0.363247626
0.762608916	0.509914932	0.365184266
0.258393824	0.290842528	0.363911302
0.474301457	0.392559809	0.363732856
0.248570617	0.706924769	0.365237127
0.759912146	0.688453070	0.364942278
0.752740600	0.095360146	0.363541194
0.502173952	0.984713469	0.362270982
0.975044131	0.393856392	0.363174193
0.481399434	0.808433877	0.365567180
0.975975070	0.609663627	0.365353339
0.474503857	0.212471073	0.364107568
0.980753575	0.011715923	0.365781212
0.986833249	0.196244135	0.363702774
0.487191983	0.615736639	0.364937701
0.002344705	0.789351239	0.363092559
0.316477487	0.397647837	0.415355791
0.823084045	0.001913662	0.418571554
0.324519069	0.802623082	0.418753488
0.827601140	0.600031114	0.420510973
0.819535780	0.398148646	0.413798795
0.325006784	0.000656232	0.409404755
0.324380105	0.200991456	0.419158064
0.824042147	0.193159873	0.416797420
0.826177781	0.803695433	0.410428238
0.327333026	0.605965765	0.417568942
0.072840294	0.102021866	0.417151451
0.576217355	0.300300364	0.414311790
0.571186382	0.901731141	0.417913055
0.073927589	0.499874301	0.409270069
0.077445711	0.697896518	0.416373198
0.581561252	0.707450873	0.413806098
0.074892099	0.298916759	0.411985425
0.569003575	0.100403627	0.407821846
0.573858160	0.502440129	0.408030401
0.074502923	0.900221780	0.410373007
0.160398869	0.602880799	0.317514338
0.665295604	0.800486149	0.321056911
0.159329817	0.002403710	0.319094603
0.661903078	0.401194274	0.320532445
0.652930918	0.196190638	0.315012496
0.158012363	0.203998134	0.312465232
0.661757004	0.001789931	0.311182840

0.162156184	0.402367086	0.320307050
0.659993728	0.600131373	0.314657947
0.162171405	0.799960449	0.311244202
0.909634844	0.901181094	0.319571250
0.416657549	0.504574414	0.315508951
0.408559789	0.098402623	0.318157441
0.908230644	0.297032346	0.311596734
0.917374337	0.504146555	0.313562693
0.410288366	0.900888834	0.310433578
0.407531779	0.301856344	0.308904839
0.411143395	0.709378365	0.312219947
0.910806416	0.700447208	0.310495922
0.910368237	0.099959847	0.310450745

HER_K-MoSe2_H
1.0

11.1999998093	0.0000000000	0.0000000000
0.0000000000	16.5699996948	0.0000000000
0.0000000000	0.0000000000	35.0000000000

H	K	Mo	Se
1	1	20	40

Direct

0.078424254	0.890655051	0.442079544
0.516519257	0.396438622	0.488108226
0.262089844	0.493297862	0.362435913
0.736919143	0.914698521	0.365413775
0.231983325	0.112540535	0.366957910
0.761735708	0.291607539	0.364339420
0.276430509	0.893859845	0.363312476
0.760317373	0.510800578	0.362856347
0.249250514	0.306704918	0.364315905
0.483762247	0.401662967	0.363596289
0.248087734	0.715245736	0.364014053
0.761685470	0.684779726	0.361291613
0.763100654	0.102347436	0.365138871
0.489818207	0.988244885	0.363449996
0.996628846	0.397595895	0.362148857
0.483265953	0.815979300	0.364137677
0.974999513	0.604938249	0.361885316
0.478454964	0.206903532	0.364736966
0.980416979	0.020637637	0.365553447
0.989040494	0.191616354	0.365180288
0.507742218	0.591326145	0.361218970
0.989317911	0.780220829	0.360943440
0.327685353	0.401662824	0.417845672
0.826231085	0.003852507	0.418485069
0.331618493	0.803193790	0.418730027
0.827674539	0.600515433	0.417553575
0.829856665	0.401821357	0.411827578

0.323982755	0.001544386	0.413328389
0.323647330	0.205947742	0.417563656
0.830302678	0.199330655	0.418526758
0.821285006	0.798615060	0.409271594
0.332185483	0.603108368	0.409247398
0.075834900	0.101261099	0.419042397
0.583874210	0.300837173	0.411891065
0.576138251	0.902407633	0.417620059
0.081030169	0.503958928	0.408496421
0.077523633	0.696507561	0.413723809
0.579246037	0.703798363	0.407340240
0.074937723	0.299590778	0.413419669
0.575012745	0.099478650	0.409658541
0.585752140	0.498796310	0.412965775
0.075713126	0.898892046	0.399745042
0.167512045	0.603119994	0.318411146
0.668908710	0.799838031	0.318968391
0.163710088	0.000867622	0.320278576
0.667949628	0.399311875	0.318653951
0.656963130	0.199433073	0.316668483
0.160089819	0.206684955	0.314250892
0.660054227	0.007973570	0.314221682
0.163124387	0.402277043	0.311802265
0.668939363	0.597508740	0.309275000
0.164460149	0.805812798	0.312782151
0.915814654	0.903123494	0.319551713
0.416857352	0.498808396	0.309299605
0.405945870	0.099698349	0.317676272
0.916686585	0.296257637	0.312604686
0.917582102	0.500790393	0.311029979
0.419612619	0.900626041	0.307529068
0.409475785	0.303543375	0.311051232
0.421678857	0.703256490	0.315926552
0.912227135	0.696005227	0.306182044
0.912922211	0.103394212	0.309924807

HER_2H-MoSe2_bare
1.0

11.1999998093	0.0000000000	0.0000000000
0.0000000000	16.5699996948	0.0000000000
0.0000000000	0.0000000000	35.0000000000

Mo Se
20 40
Direct

0.001638127	0.999969036	0.365591403
0.501661607	0.999926791	0.365611948
0.251494586	0.100288784	0.365581294
0.751491628	0.100336410	0.365547916
0.001335168	0.199926126	0.365522412

0.501304746	0.199929838	0.365516554
0.251487753	0.300261717	0.365474837
0.751488988	0.300207443	0.365488597
0.001112397	0.399999994	0.365470695
0.501130445	0.399999994	0.365459578
0.251487753	0.499738301	0.365474837
0.751488988	0.499792574	0.365488597
0.001335168	0.600073877	0.365522412
0.501304746	0.600070193	0.365516554
0.251494586	0.699711204	0.365581294
0.751491628	0.699663607	0.365547916
0.001638127	0.800030953	0.365591403
0.501661607	0.800073255	0.365611948
0.251504804	0.899999965	0.365650259
0.751473491	0.899999965	0.365579905
0.168959916	0.000062255	0.316814477
0.668885123	0.000071882	0.316784613
0.419147958	0.099706565	0.316813660
0.919194578	0.099780710	0.316777829
0.169161561	0.200072156	0.316759409
0.669109407	0.200136919	0.316720663
0.418952966	0.299866405	0.316638293
0.918952413	0.299805541	0.316651317
0.169157144	0.399999994	0.316671944
0.669248924	0.399999994	0.316708864
0.418952966	0.500133583	0.316638293
0.918952413	0.500194418	0.316651317
0.169161561	0.599927861	0.316759409
0.669109407	0.599863113	0.316720663
0.419147958	0.700293423	0.316813660
0.919194578	0.700219293	0.316777829
0.168959916	0.799937715	0.316814477
0.668885123	0.799928103	0.316784613
0.419248350	0.899999965	0.316897311
0.919277854	0.899999965	0.316868891
0.168893095	0.000192343	0.414421981
0.669015657	0.000131802	0.414373289
0.419185467	0.099874379	0.414339474
0.919161199	0.099917538	0.414332390
0.169129417	0.200273380	0.414288957
0.669090632	0.200256790	0.414310128
0.418901536	0.299976909	0.414315987
0.918912477	0.299898549	0.414329202
0.169170427	0.399999994	0.414266096
0.669136868	0.399999994	0.414247704
0.418901536	0.500023079	0.414315987
0.918912477	0.500101468	0.414329202
0.169129417	0.599726594	0.414288957
0.669090632	0.599743170	0.414310128

0.419185467	0.700125652	0.414339474
0.919161199	0.700082487	0.414332390
0.168893095	0.799807642	0.414421981
0.669015657	0.799868247	0.414373289
0.419363898	0.899999965	0.414378248
0.919247966	0.899999965	0.414305823

HER_2H-MoSe2_H
1.0

11.1999998093	0.0000000000	0.0000000000
0.0000000000	16.5699996948	0.0000000000
0.0000000000	0.0000000000	35.0000000000

H Mo Se
1 20 40
Direct

0.288759134	0.399999994	0.446465710
0.001560121	0.999635566	0.365711403
0.501649899	0.999666646	0.365582521
0.251899068	0.099694788	0.365877669
0.751509253	0.100071208	0.365306881
0.002133182	0.199560297	0.365440287
0.502022982	0.199235475	0.365411050
0.250634768	0.301235449	0.367027201
0.751178448	0.298713017	0.364481081
0.994852628	0.399999994	0.361842891
0.501436131	0.399999994	0.365562875
0.250634768	0.498764597	0.367027201
0.751178448	0.501286971	0.364481081
0.002133182	0.600439749	0.365440287
0.502022982	0.600764528	0.365411050
0.251899068	0.700305222	0.365877669
0.751509253	0.699928817	0.365306881
0.001560121	0.800364480	0.365711403
0.501649899	0.800333400	0.365582521
0.251957055	0.899999965	0.365732683
0.751659883	0.899999965	0.365572221
0.169106363	0.000053403	0.316935566
0.668784008	0.999644084	0.316741916
0.418334781	0.099339527	0.316790690
0.919544798	0.099413693	0.316684178
0.170469447	0.200745469	0.317636899
0.667704398	0.198859243	0.315975244
0.416612590	0.300215500	0.317268971
0.916688884	0.296470242	0.314615631
0.169608532	0.399999994	0.317125211
0.667971129	0.399999994	0.316447449
0.416612590	0.499784459	0.317268971
0.916688884	0.503529746	0.314615631
0.170469447	0.599254534	0.317636899

0.667704398	0.601140760	0.315975244
0.418334781	0.700660447	0.316790690
0.919544798	0.700586317	0.316684178
0.169106363	0.799946578	0.316935566
0.668784008	0.800355904	0.316741916
0.418908944	0.899999965	0.316745785
0.918958458	0.899999965	0.316823687
0.168904622	0.999783366	0.414643833
0.669133845	0.000096712	0.414235306
0.419544202	0.099463499	0.414502335
0.918631484	0.100202057	0.414309638
0.167927979	0.199024035	0.414635113
0.670233593	0.200272660	0.414159448
0.421508984	0.299513828	0.414538193
0.919374328	0.300977203	0.413145256
0.176624925	0.399999994	0.420765850
0.670987847	0.399999994	0.413207763
0.421508984	0.500486219	0.414538193
0.919374328	0.499022786	0.413145256
0.167927979	0.600975982	0.414635113
0.670233593	0.599727342	0.414159448
0.419544202	0.700536475	0.414502335
0.918631484	0.699797938	0.414309638
0.168904622	0.800216565	0.414643833
0.669133845	0.799903297	0.414235306
0.419392891	0.899999965	0.414533888
0.918492605	0.899999965	0.414563370

HER_LSC_bare
1.0

11.1999998093	0.0000000000	0.0000000000
0.0000000000	16.5699996948	0.0000000000
0.0000000000	0.0000000000	35.0000000000

La	Sr	Co	O
18	18	24	84

Direct

0.000000000	0.000000000	0.000000000
0.500000000	0.000000000	0.000000000
0.000000000	0.333333353	0.000000000
0.500000000	0.333333353	0.000000000
0.000000000	0.666666705	0.000000000
0.500000000	0.666666705	0.000000000
0.249864804	0.165285302	0.107215697
0.750135196	0.165285302	0.107215697
0.245824162	0.500000000	0.106625257
0.754175880	0.500000000	0.106625257
0.249864804	0.834714713	0.107215697
0.750135196	0.834714713	0.107215697
-0.000000000	-0.000000000	0.205242961

0.500000000	-0.000000000	0.205064147
0.000000000	0.332768429	0.205651460
0.500000000	0.335208615	0.205607741
0.000000000	0.667231543	0.205651460
0.500000000	0.664791357	0.205607741
0.250000000	0.166666676	0.000000000
0.749999979	0.166666676	0.000000000
0.250000000	0.500000000	0.000000000
0.749999979	0.500000000	0.000000000
0.250000000	0.833333295	0.000000000
0.749999979	0.833333295	0.000000000
-0.000000000	-0.000000000	0.107296671
0.500000000	-0.000000000	0.107187884
-0.000000000	0.333059135	0.107253068
0.500000000	0.333265956	0.106984745
0.000000000	0.666940894	0.107253068
0.500000000	0.666734044	0.106984745
0.250538443	0.165820528	0.210618005
0.749461578	0.165820528	0.210618005
0.249869657	0.500000000	0.210943386
0.750130343	0.500000000	0.210943386
0.250538443	0.834179458	0.210618005
0.749461578	0.834179458	0.210618005
0.250000000	-0.000000000	0.055084293
0.749999979	-0.000000000	0.055084293
-0.000000000	0.166666676	0.055084293
0.500000000	0.166666676	0.055084293
0.250000000	0.333333353	0.055084293
0.749999979	0.333333353	0.055084293
0.000000000	0.500000000	0.055084293
0.500000000	0.500000000	0.055084293
0.250000000	0.666666705	0.055084293
0.749999979	0.666666705	0.055084293
0.000000000	0.833333295	0.055084293
0.500000000	0.833333295	0.055084293
0.249905505	0.000000000	0.162779358
0.750094495	0.000000000	0.162779358
0.000000000	0.166255638	0.163111496
0.500000000	0.166125939	0.163219125
0.250146095	0.333180719	0.163902542
0.749853947	0.333180719	0.163902542
0.000000000	0.500000000	0.164065702
0.500000000	0.500000000	0.162882096
0.250146095	0.666819281	0.163902542
0.749853947	0.666819281	0.163902542
0.000000000	0.833744348	0.163111496
0.500000000	0.833874075	0.163219125
0.250000000	0.000000000	0.000000000
0.749999979	0.000000000	0.000000000

0.000000000	0.166666676	0.000000000
0.500000000	0.166666676	0.000000000
0.250000000	0.333333353	0.000000000
0.749999979	0.333333353	0.000000000
0.000000000	0.500000000	0.000000000
0.500000000	0.500000000	0.000000000
0.250000000	0.666666705	0.000000000
0.749999979	0.666666705	0.000000000
0.000000000	0.833333295	0.000000000
0.500000000	0.833333295	0.000000000
0.125000000	0.083333338	0.055134330
0.374999989	0.083333338	0.055134330
0.625000011	0.083333338	0.055134330
0.875000032	0.083333338	0.055134330
0.125000000	0.250000000	0.055134330
0.374999989	0.250000000	0.055134330
0.625000011	0.250000000	0.055134330
0.875000032	0.250000000	0.055134330
0.125000000	0.416666647	0.055134330
0.374999989	0.416666647	0.055134330
0.625000011	0.416666647	0.055134330
0.875000032	0.416666647	0.055134330
0.125000000	0.583333295	0.055134330
0.374999989	0.583333295	0.055134330
0.625000011	0.583333295	0.055134330
0.875000032	0.583333295	0.055134330
0.125000000	0.750000000	0.055134330
0.374999989	0.750000000	0.055134330
0.625000011	0.750000000	0.055134330
0.875000032	0.750000000	0.055134330
0.125000000	0.916666705	0.055134330
0.374999989	0.916666705	0.055134330
0.625000011	0.916666705	0.055134330
0.875000032	0.916666705	0.055134330
0.248212882	-0.000000000	0.108096695
0.751787096	-0.000000000	0.108096695
-0.000000000	0.165516929	0.110247401
0.500000000	0.166003450	0.110634783
0.250275820	0.335345076	0.110407203
0.749724180	0.335345076	0.110407203
0.000000000	0.500000000	0.111009598
0.500000000	0.500000000	0.109937859
0.250275820	0.664654895	0.110407203
0.749724180	0.664654895	0.110407203
0.000000000	0.834483114	0.110247401
0.500000000	0.833996550	0.110634783
0.125244347	0.081903589	0.162875393
0.373643302	0.082133533	0.161800316
0.626356655	0.082133533	0.161800316

0.874755653	0.081903589	0.162875393
0.124986035	0.250799084	0.163169765
0.375759906	0.251158337	0.163854476
0.624240094	0.251158337	0.163854476
0.875013911	0.250799084	0.163169765
0.124371852	0.415402468	0.163725471
0.375547458	0.415487907	0.163736970
0.624452542	0.415487907	0.163736970
0.875628180	0.415402468	0.163725471
0.124371852	0.584597532	0.163725471
0.375547458	0.584512122	0.163736970
0.624452542	0.584512122	0.163736970
0.875628180	0.584597532	0.163725471
0.124986035	0.749200859	0.163169765
0.375759906	0.748841663	0.163854476
0.624240094	0.748841663	0.163854476
0.875013911	0.749200859	0.163169765
0.125244347	0.918096411	0.162875393
0.373643302	0.917866481	0.161800316
0.626356655	0.917866481	0.161800316
0.874755653	0.918096411	0.162875393
0.251892660	-0.000000000	0.216935022
0.748107361	-0.000000000	0.216935022
-0.000000000	0.165259316	0.217623574
0.500000000	0.160933810	0.217496777
0.249469519	0.332981207	0.217951107
0.750530460	0.332981207	0.217951107
0.000000000	0.500000000	0.218345751
0.500000000	0.500000000	0.217926693
0.249469519	0.667018822	0.217951107
0.750530460	0.667018822	0.217951107
0.000000000	0.834740670	0.217623574
0.500000000	0.839066219	0.217496777

HER_LSC_H
1.0

11.1999998093	0.0000000000	0.0000000000
0.0000000000	16.5699996948	0.0000000000
0.0000000000	0.0000000000	35.0000000000

H	La	Sr	Co	O
1	18	18	24	84

Direct

-0.000000000	-0.000000000	0.295378739
0.000000000	0.000000000	0.000000000
0.500000000	0.000000000	0.000000000
0.000000000	0.333333353	0.000000000
0.500000000	0.333333353	0.000000000
0.000000000	0.666666705	0.000000000
0.500000000	0.666666705	0.000000000

0.249864804	0.165285302	0.107215697
0.750135196	0.165285302	0.107215697
0.245824162	0.500000000	0.106625257
0.754175880	0.500000000	0.106625257
0.249864804	0.834714713	0.107215697
0.750135196	0.834714713	0.107215697
-0.000000000	-0.000000000	0.204943453
0.500000000	-0.000000000	0.205215331
-0.000000000	0.332434096	0.205710357
0.500000000	0.335331004	0.205604839
-0.000000000	0.667565875	0.205710357
0.500000000	0.664668996	0.205604839
0.250000000	0.166666676	0.000000000
0.749999979	0.166666676	0.000000000
0.250000000	0.500000000	0.000000000
0.749999979	0.500000000	0.000000000
0.250000000	0.833333295	0.000000000
0.749999979	0.833333295	0.000000000
-0.000000000	-0.000000000	0.107296671
0.500000000	-0.000000000	0.107187884
-0.000000000	0.333059135	0.107253068
0.500000000	0.333265956	0.106984745
0.000000000	0.666940894	0.107253068
0.500000000	0.666734044	0.106984745
0.250898604	0.165941708	0.210639913
0.749101396	0.165941708	0.210639913
0.249815758	0.500000000	0.210955375
0.750184242	0.500000000	0.210955375
0.250898604	0.834058306	0.210639913
0.749101396	0.834058306	0.210639913
0.250000000	-0.000000000	0.055084293
0.749999979	-0.000000000	0.055084293
-0.000000000	0.166666676	0.055084293
0.500000000	0.166666676	0.055084293
0.250000000	0.333333353	0.055084293
0.749999979	0.333333353	0.055084293
0.000000000	0.500000000	0.055084293
0.500000000	0.500000000	0.055084293
0.250000000	0.666666705	0.055084293
0.749999979	0.666666705	0.055084293
0.000000000	0.833333295	0.055084293
0.500000000	0.833333295	0.055084293
0.250430793	-0.000000000	0.162764740
0.749569207	-0.000000000	0.162764740
-0.000000000	0.166397826	0.163070570
0.500000000	0.166138688	0.163205242
0.250045810	0.333198819	0.163893686
0.749954168	0.333198819	0.163893686
0.000000000	0.500000000	0.164093563

0.500000000	0.500000000	0.162888718
0.250045810	0.666801152	0.163893686
0.749954168	0.666801152	0.163893686
-0.000000000	0.833602189	0.163070570
0.500000000	0.833861298	0.163205242
0.250000000	0.000000000	0.000000000
0.749999979	0.000000000	0.000000000
0.000000000	0.166666676	0.000000000
0.500000000	0.166666676	0.000000000
0.250000000	0.333333353	0.000000000
0.749999979	0.333333353	0.000000000
0.000000000	0.500000000	0.000000000
0.500000000	0.500000000	0.000000000
0.250000000	0.666666705	0.000000000
0.749999979	0.666666705	0.000000000
0.000000000	0.833333295	0.000000000
0.500000000	0.833333295	0.000000000
0.125000000	0.083333338	0.055134330
0.374999989	0.083333338	0.055134330
0.625000011	0.083333338	0.055134330
0.875000032	0.083333338	0.055134330
0.125000000	0.250000000	0.055134330
0.374999989	0.250000000	0.055134330
0.625000011	0.250000000	0.055134330
0.875000032	0.250000000	0.055134330
0.125000000	0.416666647	0.055134330
0.374999989	0.416666647	0.055134330
0.625000011	0.416666647	0.055134330
0.875000032	0.416666647	0.055134330
0.125000000	0.583333295	0.055134330
0.374999989	0.583333295	0.055134330
0.625000011	0.583333295	0.055134330
0.875000032	0.583333295	0.055134330
0.125000000	0.750000000	0.055134330
0.374999989	0.750000000	0.055134330
0.625000011	0.750000000	0.055134330
0.875000032	0.750000000	0.055134330
0.125000000	0.916666705	0.055134330
0.374999989	0.916666705	0.055134330
0.625000011	0.916666705	0.055134330
0.875000032	0.916666705	0.055134330
0.248212882	-0.000000000	0.108096695
0.751787096	-0.000000000	0.108096695
-0.000000000	0.165516929	0.110247401
0.500000000	0.166003450	0.110634783
0.250275820	0.335345076	0.110407203
0.749724180	0.335345076	0.110407203
0.000000000	0.500000000	0.111009598
0.500000000	0.500000000	0.109937859

0.250275820	0.664654895	0.110407203
0.749724180	0.664654895	0.110407203
0.000000000	0.834483114	0.110247401
0.500000000	0.833996550	0.110634783
0.125418467	0.081966669	0.162951892
0.373767450	0.082213742	0.161726202
0.626232507	0.082213742	0.161726202
0.874581522	0.081966669	0.162951892
0.125045214	0.250723342	0.163041210
0.375878264	0.251295604	0.163914013
0.624121736	0.251295604	0.163914013
0.874954732	0.250723342	0.163041210
0.124341986	0.415340798	0.163747488
0.375599910	0.415598411	0.163770539
0.624400090	0.415598411	0.163770539
0.875658067	0.415340798	0.163747488
0.124341986	0.584659173	0.163747488
0.375599910	0.584401560	0.163770539
0.624400090	0.584401560	0.163770539
0.875658067	0.584659173	0.163747488
0.125045214	0.749276715	0.163041210
0.375878264	0.748704453	0.163914013
0.624121736	0.748704453	0.163914013
0.874954732	0.749276715	0.163041210
0.125418467	0.918033389	0.162951892
0.373767450	0.917786251	0.161726202
0.626232507	0.917786251	0.161726202
0.874581522	0.918033389	0.162951892
0.253678735	0.000000000	0.216922351
0.746321265	0.000000000	0.216922351
0.000000000	0.166136055	0.217558738
0.500000000	0.160877349	0.217507226
0.249062398	0.332887652	0.217960181
0.750937645	0.332887652	0.217960181
-0.000000000	0.500000000	0.218339661
0.500000000	0.500000000	0.217944036
0.249062398	0.667112348	0.217960181
0.750937645	0.667112348	0.217960181
0.000000000	0.833863888	0.217558738
0.500000000	0.839122679	0.217507226

Structures for the intermediates in OER in a POSCAR style.

OER_LSC/K-MoSe2_bare
1.0

11.1999998093	0.0000000000	0.0000000000
0.0000000000	16.5699996948	0.0000000000
0.0000000000	0.0000000000	35.0000000000

K Mo Se La Sr Co O

1	8	16	18	18	24	84

Direct

0.493211244	0.199680240	0.486435372
0.996218000	-0.000000000	0.365928841
0.496218000	-0.000000000	0.365928841
0.259126046	0.093587013	0.365618951
0.759126046	0.093587013	0.365618951
0.991637298	0.188748712	0.364897455
0.491637298	0.188748712	0.364897455
0.242296930	0.311316569	0.364960235
0.742296930	0.311316569	0.364960235
0.334845042	-0.000000000	0.419420569
0.834845063	-0.000000000	0.419420569
0.085303173	0.098975791	0.416193526
0.585303189	0.098975791	0.416193526
0.327459877	0.202735005	0.413834626
0.827459877	0.202735005	0.413834626
0.077551035	0.297099167	0.413750567
0.577551009	0.297099167	0.413750567
0.166621552	-0.000000000	0.314193453
0.666621552	-0.000000000	0.314193453
0.418432107	0.098376047	0.312279483
0.918432149	0.098376047	0.312279483
0.168383241	0.199207950	0.317593602
0.668383252	0.199207950	0.317593602
0.418405710	0.300765633	0.317174230
0.918405753	0.300765633	0.317174230
0.000000000	0.000000000	0.000000000
0.500000000	0.000000000	0.000000000
0.000000000	0.333333353	0.000000000
0.500000000	0.333333353	0.000000000
0.000000000	0.666666705	0.000000000
0.500000000	0.666666705	0.000000000
0.249917852	0.166346660	0.107885224
0.749917895	0.166346660	0.107885224
0.249908911	0.500000000	0.106344155
0.749908954	0.500000000	0.106344155
0.249917852	0.833653354	0.107885224
0.749917895	0.833653354	0.107885224
0.988429546	0.007852866	0.212044607
0.489068125	0.008371841	0.211948272
0.979953851	0.338931916	0.212762560
0.485225158	0.341734924	0.212503624
0.982840589	0.674722807	0.209651893
0.481847831	0.674485108	0.209832954
0.250000000	0.166666676	0.000000000
0.749999979	0.166666676	0.000000000
0.250000000	0.500000000	0.000000000
0.749999979	0.500000000	0.000000000

0.250000000	0.833333295	0.000000000
0.749999979	0.833333295	0.000000000
0.999390074	0.000000000	0.107119492
0.499390074	0.000000000	0.107119492
0.999982970	0.332969955	0.107268674
0.499982970	0.332969955	0.107268674
0.999982970	0.667030045	0.107268674
0.499982970	0.667030045	0.107268674
0.242570047	0.170446870	0.217207786
0.741948753	0.170538395	0.217308739
0.242576497	0.505016661	0.213640458
0.742133954	0.504856027	0.213666003
0.245005705	0.836212102	0.214758532
0.743943551	0.836750580	0.214820671
0.250000000	-0.000000000	0.055084293
0.749999979	-0.000000000	0.055084293
-0.000000000	0.166666676	0.055084293
0.500000000	0.166666676	0.055084293
0.250000000	0.333333353	0.055084293
0.749999979	0.333333353	0.055084293
0.000000000	0.500000000	0.055084293
0.500000000	0.500000000	0.055084293
0.250000000	0.666666705	0.055084293
0.749999979	0.666666705	0.055084293
0.000000000	0.833333295	0.055084293
0.500000000	0.833333295	0.055084293
0.247688298	0.002711946	0.164928545
0.747674205	0.002619649	0.164789554
0.997718079	0.167592681	0.164731162
0.497265424	0.167827546	0.164689636
0.245224902	0.334830253	0.165947614
0.744631729	0.334746770	0.164582184
0.998827321	0.499735768	0.164907755
0.498627858	0.500701702	0.164411572
0.246810853	0.667858597	0.165261773
0.747032349	0.668042885	0.165267576
0.996726172	0.834788382	0.165345124
0.496916822	0.835009103	0.165253026
0.250000000	0.000000000	0.000000000
0.749999979	0.000000000	0.000000000
0.000000000	0.166666676	0.000000000
0.500000000	0.166666676	0.000000000
0.250000000	0.333333353	0.000000000
0.749999979	0.333333353	0.000000000
0.000000000	0.500000000	0.000000000
0.500000000	0.500000000	0.000000000
0.250000000	0.666666705	0.000000000
0.749999979	0.666666705	0.000000000
0.000000000	0.833333295	0.000000000

0.500000000	0.833333295	0.000000000
0.125000000	0.083333338	0.055134330
0.374999989	0.083333338	0.055134330
0.625000011	0.083333338	0.055134330
0.875000032	0.083333338	0.055134330
0.125000000	0.250000000	0.055134330
0.374999989	0.250000000	0.055134330
0.625000011	0.250000000	0.055134330
0.875000032	0.250000000	0.055134330
0.125000000	0.416666647	0.055134330
0.374999989	0.416666647	0.055134330
0.625000011	0.416666647	0.055134330
0.875000032	0.416666647	0.055134330
0.125000000	0.583333295	0.055134330
0.374999989	0.583333295	0.055134330
0.625000011	0.583333295	0.055134330
0.875000032	0.583333295	0.055134330
0.125000000	0.750000000	0.055134330
0.374999989	0.750000000	0.055134330
0.625000011	0.750000000	0.055134330
0.875000032	0.750000000	0.055134330
0.125000000	0.916666705	0.055134330
0.374999989	0.916666705	0.055134330
0.625000011	0.916666705	0.055134330
0.875000032	0.916666705	0.055134330
0.249987057	-0.000000000	0.110441978
0.749987036	-0.000000000	0.110441978
0.000314729	0.167628149	0.110136305
0.500314713	0.167628149	0.110136305
0.250063713	0.329844902	0.110218402
0.750063756	0.329844902	0.110218402
0.999881898	0.500000000	0.109866755
0.499881940	0.500000000	0.109866755
0.250063713	0.670155127	0.110218402
0.750063756	0.670155127	0.110218402
0.000314729	0.832371851	0.110136305
0.500314713	0.832371851	0.110136305
0.142295395	0.100432634	0.163656521
0.397545787	0.073425233	0.161648083
0.642375787	0.100612434	0.163741316
0.897829463	0.073317017	0.161725208
0.096448449	0.263917286	0.168642017
0.355918924	0.234770175	0.164509310
0.596037986	0.264212079	0.168477127
0.856401169	0.235022968	0.165006283
0.142933654	0.432730420	0.160892555
0.392759151	0.407299545	0.162161200
0.642869952	0.432738592	0.161339283
0.894300357	0.406869011	0.162004907

0.100874264	0.594973877	0.167694201
0.360413711	0.572870791	0.163480881
0.602600329	0.596199783	0.167967919
0.861151063	0.571376740	0.163208934
0.134347275	0.763428161	0.164498547
0.388958376	0.743561749	0.161725535
0.634721528	0.764502871	0.164205565
0.888382467	0.742661254	0.161453669
0.101012302	0.930244046	0.168230670
0.357661296	0.902490281	0.162521185
0.600615026	0.930817920	0.168172373
0.857046091	0.902507087	0.162479251
0.268568018	0.002598879	0.217848751
0.768471969	0.002390050	0.217758805
0.995241250	0.156589700	0.218804414
0.494941567	0.156364015	0.218840354
0.260513067	0.338233581	0.218636036
0.759363698	0.338457640	0.218202441
0.997990813	0.487353196	0.219395924
0.497516785	0.487681198	0.219926507
0.265296958	0.669165136	0.220699487
0.766266597	0.668929164	0.220384571
0.990508727	0.826822410	0.219788047
0.491344980	0.826241112	0.219670255

OER_LSC/K-MoSe2_OH
1.0

11.1999998093	0.0000000000	0.0000000000
0.0000000000	16.5699996948	0.0000000000
0.0000000000	0.0000000000	35.0000000000

K	H	O	Mo	Se	La	Sr	Co
1	1	85	8	16	18	18	24

Direct

0.495003896	0.199926083	0.486514555
0.524739206	0.667093815	0.304968316
0.517857850	0.661853844	0.277495820
0.250000000	0.000000000	0.000000000
0.749999979	0.000000000	0.000000000
0.000000000	0.166666676	0.000000000
0.500000000	0.166666676	0.000000000
0.250000000	0.333333353	0.000000000
0.749999979	0.333333353	0.000000000
0.000000000	0.500000000	0.000000000
0.500000000	0.500000000	0.000000000
0.250000000	0.666666705	0.000000000
0.749999979	0.666666705	0.000000000
0.000000000	0.833333295	0.000000000
0.500000000	0.833333295	0.000000000
0.125000000	0.083333338	0.055134330

0.374999989	0.083333338	0.055134330
0.625000011	0.083333338	0.055134330
0.875000032	0.083333338	0.055134330
0.125000000	0.250000000	0.055134330
0.374999989	0.250000000	0.055134330
0.625000011	0.250000000	0.055134330
0.875000032	0.250000000	0.055134330
0.125000000	0.416666647	0.055134330
0.374999989	0.416666647	0.055134330
0.625000011	0.416666647	0.055134330
0.875000032	0.416666647	0.055134330
0.125000000	0.583333295	0.055134330
0.374999989	0.583333295	0.055134330
0.625000011	0.583333295	0.055134330
0.875000032	0.583333295	0.055134330
0.125000000	0.750000000	0.055134330
0.374999989	0.750000000	0.055134330
0.625000011	0.750000000	0.055134330
0.875000032	0.750000000	0.055134330
0.125000000	0.916666705	0.055134330
0.374999989	0.916666705	0.055134330
0.625000011	0.916666705	0.055134330
0.875000032	0.916666705	0.055134330
0.249987057	-0.000000000	0.110441978
0.749987036	-0.000000000	0.110441978
0.000314729	0.167628149	0.110136305
0.500314713	0.167628149	0.110136305
0.250063713	0.329844902	0.110218402
0.750063756	0.329844902	0.110218402
0.999881898	0.500000000	0.109866755
0.499881940	0.500000000	0.109866755
0.250063713	0.670155127	0.110218402
0.750063756	0.670155127	0.110218402
0.000314729	0.832371851	0.110136305
0.500314713	0.832371851	0.110136305
0.140024586	0.098608753	0.163980920
0.398778113	0.073343585	0.161849703
0.642992269	0.101712821	0.164155252
0.896797025	0.072542329	0.161560958
0.097119033	0.263779644	0.168380737
0.356066297	0.233754443	0.165250165
0.595775086	0.264590958	0.168566922
0.855953538	0.234897125	0.164145252
0.140260248	0.432265784	0.162364987
0.390712072	0.403929253	0.161475945
0.647942903	0.433519316	0.161127349
0.896620851	0.407122595	0.162049729
0.104334739	0.595822227	0.165997451
0.354427765	0.572331277	0.162669972

0.609848680	0.599683141	0.168136106
0.857287064	0.572323623	0.161938831
0.134718133	0.763400534	0.163809504
0.382626191	0.740868324	0.163319520
0.646606567	0.763100101	0.164004476
0.892843194	0.738593721	0.161313656
0.098314617	0.929256242	0.168148872
0.350497456	0.902766599	0.161730180
0.601234659	0.933233473	0.167295361
0.858030164	0.900562616	0.162529142
0.267937060	0.003448634	0.217831979
0.769430497	0.000937077	0.217968995
0.993676032	0.158580804	0.218923255
0.495440500	0.157809016	0.218899182
0.257339780	0.337570671	0.218642058
0.764362399	0.337501548	0.218128722
0.996154223	0.494677438	0.219619451
0.495213151	0.487985315	0.217484515
0.252622753	0.668135260	0.218733665
0.773429628	0.669230000	0.218290806
0.991701586	0.826450955	0.219273554
0.493020211	0.829052753	0.218311732
0.996218000	-0.000000000	0.365928841
0.496218000	-0.000000000	0.365928841
0.259126046	0.093587013	0.365618951
0.759126046	0.093587013	0.365618951
0.991637298	0.188748712	0.364897455
0.491637298	0.188748712	0.364897455
0.242296930	0.311316569	0.364960235
0.742296930	0.311316569	0.364960235
0.334845042	-0.000000000	0.419420569
0.834845063	-0.000000000	0.419420569
0.085303173	0.098975791	0.416193526
0.585303189	0.098975791	0.416193526
0.327459877	0.202735005	0.413834626
0.827459877	0.202735005	0.413834626
0.077551035	0.297099167	0.413750567
0.577551009	0.297099167	0.413750567
0.166621552	-0.000000000	0.314193453
0.666621552	-0.000000000	0.314193453
0.418432107	0.098376047	0.312279483
0.918432149	0.098376047	0.312279483
0.168383241	0.199207950	0.317593602
0.668383252	0.199207950	0.317593602
0.418405710	0.300765633	0.317174230
0.918405753	0.300765633	0.317174230
0.000000000	0.000000000	0.000000000
0.500000000	0.000000000	0.000000000
0.000000000	0.333333353	0.000000000

0.500000000	0.333333353	0.000000000
0.000000000	0.666666705	0.000000000
0.500000000	0.666666705	0.000000000
0.249917852	0.166346660	0.107885224
0.749917895	0.166346660	0.107885224
0.249908911	0.500000000	0.106344155
0.749908954	0.500000000	0.106344155
0.249917852	0.833653354	0.107885224
0.749917895	0.833653354	0.107885224
0.988474164	0.007825494	0.211863109
0.490577442	0.009300497	0.211912196
0.985753808	0.341670377	0.212312085
0.476470044	0.336612680	0.212025234
0.991530435	0.670878009	0.209662533
0.485692586	0.672357614	0.215844985
0.250000000	0.166666676	0.000000000
0.749999979	0.166666676	0.000000000
0.250000000	0.500000000	0.000000000
0.749999979	0.500000000	0.000000000
0.250000000	0.833333295	0.000000000
0.749999979	0.833333295	0.000000000
0.999390074	0.000000000	0.107119492
0.499390074	0.000000000	0.107119492
0.999982970	0.332969955	0.107268674
0.499982970	0.332969955	0.107268674
0.999982970	0.667030045	0.107268674
0.499982970	0.667030045	0.107268674
0.240035849	0.169734060	0.217322690
0.743267975	0.172803919	0.217343957
0.241400071	0.505695053	0.214109162
0.744431883	0.506954168	0.214088876
0.243557504	0.834987577	0.214373561
0.748270848	0.834200523	0.215873010
0.250000000	-0.000000000	0.055084293
0.749999979	-0.000000000	0.055084293
-0.000000000	0.166666676	0.055084293
0.500000000	0.166666676	0.055084293
0.250000000	0.333333353	0.055084293
0.749999979	0.333333353	0.055084293
0.000000000	0.500000000	0.055084293
0.500000000	0.500000000	0.055084293
0.250000000	0.666666705	0.055084293
0.749999979	0.666666705	0.055084293
0.000000000	0.833333295	0.055084293
0.500000000	0.833333295	0.055084293
0.247674993	0.001393461	0.164993150
0.747742495	0.002731465	0.165041801
0.996641023	0.167231053	0.164829268
0.498146040	0.167708322	0.164606857

0.243441594	0.334172062	0.164585604
0.746161184	0.335465796	0.164981501
0.998922689	0.500490708	0.164014639
0.499190952	0.501071718	0.164649923
0.248473121	0.667363918	0.164364951
0.749294941	0.669139064	0.165077972
0.995798979	0.833983601	0.165333707
0.495655630	0.834647604	0.165321486

OER_LSC/K-MoSe2_Vo_OO
1.0

11.1999998093	0.0000000000	0.0000000000
0.0000000000	16.5699996948	0.0000000000
0.0000000000	0.0000000000	35.0000000000

K	O	Mo	Se	La	Sr	Co
1	85	8	16	18	18	24

Direct

0.494715793	0.199776240	0.486482729
0.234564760	0.650571882	0.221670914
0.312119813	0.698935200	0.246956798
0.250000000	0.000000000	0.000000000
0.749999979	0.000000000	0.000000000
0.000000000	0.166666676	0.000000000
0.500000000	0.166666676	0.000000000
0.250000000	0.333333353	0.000000000
0.749999979	0.333333353	0.000000000
0.000000000	0.500000000	0.000000000
0.500000000	0.500000000	0.000000000
0.250000000	0.666666705	0.000000000
0.749999979	0.666666705	0.000000000
0.000000000	0.833333295	0.000000000
0.500000000	0.833333295	0.000000000
0.125000000	0.083333338	0.055134330
0.374999989	0.083333338	0.055134330
0.625000011	0.083333338	0.055134330
0.875000032	0.083333338	0.055134330
0.125000000	0.250000000	0.055134330
0.374999989	0.250000000	0.055134330
0.625000011	0.250000000	0.055134330
0.875000032	0.250000000	0.055134330
0.125000000	0.416666647	0.055134330
0.374999989	0.416666647	0.055134330
0.625000011	0.416666647	0.055134330
0.875000032	0.416666647	0.055134330
0.125000000	0.583333295	0.055134330
0.374999989	0.583333295	0.055134330
0.625000011	0.583333295	0.055134330
0.875000032	0.583333295	0.055134330
0.125000000	0.750000000	0.055134330

0.374999989	0.750000000	0.055134330
0.625000011	0.750000000	0.055134330
0.875000032	0.750000000	0.055134330
0.125000000	0.916666705	0.055134330
0.374999989	0.916666705	0.055134330
0.625000011	0.916666705	0.055134330
0.875000032	0.916666705	0.055134330
0.249987057	-0.000000000	0.110441978
0.749987036	-0.000000000	0.110441978
0.000314729	0.167628149	0.110136305
0.500314713	0.167628149	0.110136305
0.250063713	0.329844902	0.110218402
0.750063756	0.329844902	0.110218402
0.999881898	0.500000000	0.109866755
0.499881940	0.500000000	0.109866755
0.250063713	0.670155127	0.110218402
0.750063756	0.670155127	0.110218402
0.000314729	0.832371851	0.110136305
0.500314713	0.832371851	0.110136305
0.098452048	0.068543256	0.167989077
0.358255427	0.098387429	0.163430200
0.599901984	0.070475850	0.167652593
0.857662743	0.100040265	0.163132409
0.141087741	0.230462827	0.164244434
0.395982613	0.259656773	0.162656689
0.641186291	0.233176037	0.164252009
0.896996275	0.260137437	0.162560926
0.098678865	0.399076852	0.168972628
0.355686637	0.431392973	0.162112427
0.596692189	0.401363800	0.167854650
0.847302094	0.432837499	0.161322771
0.145897985	0.570663815	0.162329129
0.394692428	0.592395043	0.162943527
0.638021071	0.567304429	0.162524823
0.891928347	0.598878992	0.162016542
0.099340509	0.739144170	0.167731857
0.357166663	0.759295648	0.167390074
0.600378992	0.739400575	0.167513207
0.856111745	0.766477962	0.165084267
0.140953929	0.900789668	0.163811779
0.393892874	0.924869340	0.162003040
0.640803714	0.902569419	0.162465927
0.897680621	0.927761443	0.161929430
0.267378458	0.997226805	0.218630641
0.766665012	0.997783470	0.217567989
0.994390436	0.172571961	0.219367095
0.495214598	0.174502820	0.218972329
0.264770691	0.330245652	0.218645886
0.766131380	0.331726696	0.219146919

0.991695114	0.512554659	0.219275134
0.494347308	0.505013783	0.219494411
0.752728679	0.664083266	0.219515923
0.993659428	0.842405690	0.220547417
0.501617117	0.849823170	0.220004858
0.996218000	-0.000000000	0.365928841
0.496218000	-0.000000000	0.365928841
0.259126046	0.093587013	0.365618951
0.759126046	0.093587013	0.365618951
0.991637298	0.188748712	0.364897455
0.491637298	0.188748712	0.364897455
0.242296930	0.311316569	0.364960235
0.742296930	0.311316569	0.364960235
0.334845042	-0.000000000	0.419420569
0.834845063	-0.000000000	0.419420569
0.085303173	0.098975791	0.416193526
0.585303189	0.098975791	0.416193526
0.327459877	0.202735005	0.413834626
0.827459877	0.202735005	0.413834626
0.077551035	0.297099167	0.413750567
0.577551009	0.297099167	0.413750567
0.166621552	-0.000000000	0.314193453
0.666621552	-0.000000000	0.314193453
0.418432107	0.098376047	0.312279483
0.918432149	0.098376047	0.312279483
0.168383241	0.199207950	0.317593602
0.668383252	0.199207950	0.317593602
0.418405710	0.300765633	0.317174230
0.918405753	0.300765633	0.317174230
0.000000000	0.000000000	0.000000000
0.500000000	0.000000000	0.000000000
0.000000000	0.333333353	0.000000000
0.500000000	0.333333353	0.000000000
0.000000000	0.666666705	0.000000000
0.500000000	0.666666705	0.000000000
0.249917852	0.166346660	0.107885224
0.749917895	0.166346660	0.107885224
0.249908911	0.500000000	0.106344155
0.749908954	0.500000000	0.106344155
0.249917852	0.833653354	0.107885224
0.749917895	0.833653354	0.107885224
0.988669242	0.992257339	0.212276159
0.489999618	0.993623792	0.211827619
0.987949473	0.320167755	0.213070938
0.485374297	0.325704073	0.212592016
0.983146446	0.665499274	0.210254424
0.491133511	0.659894409	0.211284992
0.250000000	0.166666676	0.000000000
0.749999979	0.166666676	0.000000000

0.250000000	0.500000000	0.000000000
0.749999979	0.500000000	0.000000000
0.250000000	0.833333295	0.000000000
0.749999979	0.833333295	0.000000000
0.999390074	0.000000000	0.107119492
0.499390074	0.000000000	0.107119492
0.999982970	0.332969955	0.107268674
0.499982970	0.332969955	0.107268674
0.999982970	0.667030045	0.107268674
0.499982970	0.667030045	0.107268674
0.247193771	0.161475453	0.217136369
0.741820603	0.161373194	0.216936643
0.242691811	0.492755010	0.214099339
0.743774955	0.497089998	0.214076356
0.250293979	0.833973241	0.217598575
0.741294124	0.833544519	0.215633106
0.250000000	-0.000000000	0.055084293
0.749999979	-0.000000000	0.055084293
-0.000000000	0.166666676	0.055084293
0.500000000	0.166666676	0.055084293
0.250000000	0.333333353	0.055084293
0.749999979	0.333333353	0.055084293
0.000000000	0.500000000	0.055084293
0.500000000	0.500000000	0.055084293
0.250000000	0.666666705	0.055084293
0.749999979	0.666666705	0.055084293
0.000000000	0.833333295	0.055084293
0.500000000	0.833333295	0.055084293
0.246554532	0.998268652	0.165736771
0.748116813	0.999275737	0.164337104
0.998341458	0.165191819	0.165203844
0.498105466	0.165995550	0.164781557
0.246436962	0.330446747	0.164849567
0.745962956	0.332285088	0.166132832
0.995745165	0.498990440	0.165628869
0.497197262	0.498717863	0.165058313
0.247271278	0.665408914	0.164621476
0.745641261	0.667325644	0.164847701
0.998525211	0.833827514	0.165057850
0.498914174	0.833250762	0.164810494

OER_LSC/K-MoSe2_Vo_OH
1.0

11.1999998093	0.0000000000	0.0000000000
0.0000000000	16.5699996948	0.0000000000
0.0000000000	0.0000000000	35.0000000000

K	H	O	Mo	Se	La	Sr	Co
1	1	84	8	16	18	18	24

Direct

0.498061529	0.200901383	0.486634936
0.268680841	0.662839691	0.265573420
0.286256527	0.657257962	0.238424546
0.250000000	0.000000000	0.000000000
0.749999979	0.000000000	0.000000000
0.000000000	0.166666676	0.000000000
0.500000000	0.166666676	0.000000000
0.250000000	0.333333353	0.000000000
0.749999979	0.333333353	0.000000000
0.000000000	0.500000000	0.000000000
0.500000000	0.500000000	0.000000000
0.250000000	0.666666705	0.000000000
0.749999979	0.666666705	0.000000000
0.000000000	0.833333295	0.000000000
0.500000000	0.833333295	0.000000000
0.125000000	0.083333338	0.055134330
0.374999989	0.083333338	0.055134330
0.625000011	0.083333338	0.055134330
0.875000032	0.083333338	0.055134330
0.125000000	0.250000000	0.055134330
0.374999989	0.250000000	0.055134330
0.625000011	0.250000000	0.055134330
0.875000032	0.250000000	0.055134330
0.125000000	0.416666647	0.055134330
0.374999989	0.416666647	0.055134330
0.625000011	0.416666647	0.055134330
0.875000032	0.416666647	0.055134330
0.125000000	0.583333295	0.055134330
0.374999989	0.583333295	0.055134330
0.625000011	0.583333295	0.055134330
0.875000032	0.583333295	0.055134330
0.125000000	0.750000000	0.055134330
0.374999989	0.750000000	0.055134330
0.625000011	0.750000000	0.055134330
0.875000032	0.750000000	0.055134330
0.125000000	0.916666705	0.055134330
0.374999989	0.916666705	0.055134330
0.625000011	0.916666705	0.055134330
0.875000032	0.916666705	0.055134330
0.249987057	-0.000000000	0.110441978
0.749987036	-0.000000000	0.110441978
0.000314729	0.167628149	0.110136305
0.500314713	0.167628149	0.110136305
0.250063713	0.329844902	0.110218402
0.750063756	0.329844902	0.110218402
0.999881898	0.500000000	0.109866755
0.499881940	0.500000000	0.109866755
0.250063713	0.670155127	0.110218402
0.750063756	0.670155127	0.110218402

0.000314729	0.832371851	0.110136305
0.500314713	0.832371851	0.110136305
0.099802104	0.071628770	0.168053722
0.359098407	0.097360610	0.164139107
0.598863288	0.069926415	0.167484651
0.857368382	0.098386529	0.163404683
0.141815514	0.233418513	0.164040865
0.395927479	0.259494153	0.162735503
0.641300093	0.233333994	0.163757610
0.895738447	0.260235970	0.162584850
0.098640037	0.403399294	0.168073109
0.355109536	0.431456974	0.161205728
0.598118784	0.402583059	0.166810158
0.855362260	0.430124218	0.161861174
0.134424793	0.571632281	0.166540582
0.387891367	0.592876484	0.165528720
0.632274971	0.570226402	0.163765144
0.888104198	0.590543522	0.161068153
0.104586686	0.739235624	0.173011480
0.360490814	0.760609785	0.167409924
0.604756059	0.741127951	0.167268957
0.864285331	0.760180430	0.166496740
0.139511932	0.903218113	0.163343866
0.392517880	0.924162171	0.162327426
0.640949618	0.901752493	0.162181323
0.893726790	0.924365338	0.161545563
0.267562083	0.997700822	0.219683824
0.766139044	0.996625478	0.217627866
0.994224991	0.176043447	0.218595028
0.496545826	0.173955911	0.218665504
0.266213826	0.334074421	0.218152482
0.761317453	0.332268742	0.217982415
0.984464986	0.505080949	0.219592244
0.495047918	0.501727434	0.219124753
0.754056756	0.661367107	0.221493108
0.995291999	0.848017347	0.221010535
0.499831702	0.845311376	0.220134803
0.996218000	-0.000000000	0.365928841
0.496218000	-0.000000000	0.365928841
0.259126046	0.093587013	0.365618951
0.759126046	0.093587013	0.365618951
0.991637298	0.188748712	0.364897455
0.491637298	0.188748712	0.364897455
0.242296930	0.311316569	0.364960235
0.742296930	0.311316569	0.364960235
0.334845042	-0.000000000	0.419420569
0.834845063	-0.000000000	0.419420569
0.085303173	0.098975791	0.416193526
0.585303189	0.098975791	0.416193526

0.327459877	0.202735005	0.413834626
0.827459877	0.202735005	0.413834626
0.077551035	0.297099167	0.413750567
0.577551009	0.297099167	0.413750567
0.166621552	-0.000000000	0.314193453
0.666621552	-0.000000000	0.314193453
0.418432107	0.098376047	0.312279483
0.918432149	0.098376047	0.312279483
0.168383241	0.199207950	0.317593602
0.668383252	0.199207950	0.317593602
0.418405710	0.300765633	0.317174230
0.918405753	0.300765633	0.317174230
0.000000000	0.000000000	0.000000000
0.500000000	0.000000000	0.000000000
0.000000000	0.333333353	0.000000000
0.500000000	0.333333353	0.000000000
0.000000000	0.666666705	0.000000000
0.500000000	0.666666705	0.000000000
0.249917852	0.166346660	0.107885224
0.749917895	0.166346660	0.107885224
0.249908911	0.500000000	0.106344155
0.749908954	0.500000000	0.106344155
0.249917852	0.833653354	0.107885224
0.749917895	0.833653354	0.107885224
0.988967861	0.993396453	0.211437021
0.482346722	0.996971839	0.211946079
0.984212432	0.327975568	0.212036010
0.487577242	0.325132041	0.212118503
0.973654321	0.657778138	0.210236291
0.502547971	0.658812561	0.210210541
0.250000000	0.166666676	0.000000000
0.749999979	0.166666676	0.000000000
0.250000000	0.500000000	0.000000000
0.749999979	0.500000000	0.000000000
0.250000000	0.833333295	0.000000000
0.749999979	0.833333295	0.000000000
0.999390074	0.000000000	0.107119492
0.499390074	0.000000000	0.107119492
0.999982970	0.332969955	0.107268674
0.499982970	0.332969955	0.107268674
0.999982970	0.667030045	0.107268674
0.499982970	0.667030045	0.107268674
0.242133847	0.160939767	0.216722516
0.744990123	0.163000066	0.216293403
0.251963696	0.496500527	0.214288752
0.741695178	0.495276003	0.213563320
0.248472056	0.837929349	0.215693133
0.744480333	0.833874766	0.215241010
0.250000000	-0.000000000	0.055084293

0.749999979	-0.000000000	0.055084293
-0.000000000	0.166666676	0.055084293
0.500000000	0.166666676	0.055084293
0.250000000	0.333333353	0.055084293
0.749999979	0.333333353	0.055084293
0.000000000	0.500000000	0.055084293
0.500000000	0.500000000	0.055084293
0.250000000	0.666666705	0.055084293
0.749999979	0.666666705	0.055084293
0.000000000	0.833333295	0.055084293
0.500000000	0.833333295	0.055084293
0.248398253	0.999728919	0.164705440
0.746609837	0.999415709	0.164933355
0.998196959	0.166291221	0.164757456
0.498074131	0.165753247	0.164636571
0.245937582	0.331288535	0.164996638
0.745804408	0.331501889	0.164417594
0.995776159	0.499018527	0.164854431
0.495651373	0.498215587	0.164500059
0.244458641	0.666905901	0.164690726
0.746001784	0.666273955	0.164808396
0.000301424	0.833098013	0.166402926
0.499249748	0.833785672	0.165834481

OER_LSC/K-MoSe2-Ho_OH
1.0

11.1999998093	0.0000000000	0.0000000000
0.0000000000	16.5699996948	0.0000000000
0.0000000000	0.0000000000	35.0000000000

K	H	O	Mo	Se	La	Sr	Co
1	2	85	8	16	18	18	24

Direct

0.494762966	0.199721219	0.486495645
0.278802902	0.664833026	0.247171892
0.403243693	0.672381672	0.296237537
0.408150220	0.672271801	0.268570655
0.250000000	0.000000000	0.000000000
0.749999979	0.000000000	0.000000000
0.000000000	0.166666676	0.000000000
0.500000000	0.166666676	0.000000000
0.250000000	0.333333353	0.000000000
0.749999979	0.333333353	0.000000000
0.000000000	0.500000000	0.000000000
0.500000000	0.500000000	0.000000000
0.250000000	0.666666705	0.000000000
0.749999979	0.666666705	0.000000000
0.000000000	0.833333295	0.000000000
0.500000000	0.833333295	0.000000000
0.125000000	0.083333338	0.055134330

0.374999989	0.083333338	0.055134330
0.625000011	0.083333338	0.055134330
0.875000032	0.083333338	0.055134330
0.125000000	0.250000000	0.055134330
0.374999989	0.250000000	0.055134330
0.625000011	0.250000000	0.055134330
0.875000032	0.250000000	0.055134330
0.125000000	0.416666647	0.055134330
0.374999989	0.416666647	0.055134330
0.625000011	0.416666647	0.055134330
0.875000032	0.416666647	0.055134330
0.125000000	0.583333295	0.055134330
0.374999989	0.583333295	0.055134330
0.625000011	0.583333295	0.055134330
0.875000032	0.583333295	0.055134330
0.125000000	0.750000000	0.055134330
0.374999989	0.750000000	0.055134330
0.625000011	0.750000000	0.055134330
0.875000032	0.750000000	0.055134330
0.125000000	0.916666705	0.055134330
0.374999989	0.916666705	0.055134330
0.625000011	0.916666705	0.055134330
0.875000032	0.916666705	0.055134330
0.249987057	-0.000000000	0.110441978
0.749987036	-0.000000000	0.110441978
0.000314729	0.167628149	0.110136305
0.500314713	0.167628149	0.110136305
0.250063713	0.329844902	0.110218402
0.750063756	0.329844902	0.110218402
0.999881898	0.500000000	0.109866755
0.499881940	0.500000000	0.109866755
0.250063713	0.670155127	0.110218402
0.750063756	0.670155127	0.110218402
0.000314729	0.832371851	0.110136305
0.500314713	0.832371851	0.110136305
0.145027938	0.099720047	0.163512925
0.402930215	0.070087121	0.167406137
0.644861939	0.096802427	0.164558397
0.902853283	0.070860399	0.166962596
0.101844489	0.261143428	0.162618514
0.358968681	0.234473555	0.164616966
0.603873570	0.259158353	0.162757805
0.859071968	0.233189245	0.164402458
0.145432026	0.432869759	0.161464637
0.399756949	0.403703209	0.167621408
0.643293272	0.428473878	0.160641234
0.901175327	0.402614656	0.167169585
0.108178984	0.592090581	0.162497507
0.368693264	0.571452942	0.167154993

0.614784498	0.588294013	0.160948140
0.866071597	0.569741623	0.162794263
0.136864762	0.764034667	0.166847188
0.397463746	0.740742798	0.172197955
0.639723295	0.764318640	0.165669005
0.897237163	0.740658308	0.166821752
0.103530290	0.927597413	0.162448175
0.356140207	0.899787648	0.161346558
0.605987065	0.927502103	0.161679173
0.857897501	0.902220294	0.160705526
0.239130284	0.997467497	0.217738179
0.743208966	0.997469223	0.218367904
0.005972174	0.172081051	0.218869972
0.505963402	0.172908754	0.218906893
0.230256174	0.333861557	0.218195575
0.735288530	0.332574730	0.217999254
0.005130122	0.503722898	0.218821498
0.518028489	0.498522352	0.218661581
0.211648263	0.660099014	0.227875137
0.748659726	0.661119509	0.219072887
0.996009554	0.849442391	0.219761072
0.504165215	0.855108207	0.219384452
0.996218000	-0.000000000	0.365928841
0.496218000	-0.000000000	0.365928841
0.259126046	0.093587013	0.365618951
0.759126046	0.093587013	0.365618951
0.991637298	0.188748712	0.364897455
0.491637298	0.188748712	0.364897455
0.242296930	0.311316569	0.364960235
0.742296930	0.311316569	0.364960235
0.334845042	-0.000000000	0.419420569
0.834845063	-0.000000000	0.419420569
0.085303173	0.098975791	0.416193526
0.585303189	0.098975791	0.416193526
0.327459877	0.202735005	0.413834626
0.827459877	0.202735005	0.413834626
0.077551035	0.297099167	0.413750567
0.577551009	0.297099167	0.413750567
0.166621552	-0.000000000	0.314193453
0.666621552	-0.000000000	0.314193453
0.418432107	0.098376047	0.312279483
0.918432149	0.098376047	0.312279483
0.168383241	0.199207950	0.317593602
0.668383252	0.199207950	0.317593602
0.418405710	0.300765633	0.317174230
0.918405753	0.300765633	0.317174230
0.000000000	0.000000000	0.000000000
0.500000000	0.000000000	0.000000000
0.000000000	0.333333353	0.000000000

0.500000000	0.333333353	0.000000000
0.000000000	0.666666705	0.000000000
0.500000000	0.666666705	0.000000000
0.249917852	0.166346660	0.107885224
0.749917895	0.166346660	0.107885224
0.249908911	0.500000000	0.106344155
0.749908954	0.500000000	0.106344155
0.249917852	0.833653354	0.107885224
0.749917895	0.833653354	0.107885224
0.012974846	0.997047581	0.212098449
0.522084586	0.999820546	0.212853541
0.008839174	0.325443381	0.212178271
0.512629799	0.328409038	0.211771897
0.001595744	0.657302222	0.209660666
0.517446322	0.657227056	0.212281213
0.250000000	0.166666676	0.000000000
0.749999979	0.166666676	0.000000000
0.250000000	0.500000000	0.000000000
0.749999979	0.500000000	0.000000000
0.250000000	0.833333295	0.000000000
0.749999979	0.833333295	0.000000000
0.999390074	0.000000000	0.107119492
0.499390074	0.000000000	0.107119492
0.999982970	0.332969955	0.107268674
0.499982970	0.332969955	0.107268674
0.999982970	0.667030045	0.107268674
0.499982970	0.667030045	0.107268674
0.256068775	0.163334802	0.216899095
0.756286821	0.162811619	0.217077732
0.251375803	0.496414599	0.214568179
0.756116011	0.493541317	0.212795871
0.259731480	0.834466136	0.216478021
0.755204231	0.837778212	0.214812279
0.250000000	-0.000000000	0.055084293
0.749999979	-0.000000000	0.055084293
-0.000000000	0.166666676	0.055084293
0.500000000	0.166666676	0.055084293
0.250000000	0.333333353	0.055084293
0.749999979	0.333333353	0.055084293
0.000000000	0.500000000	0.055084293
0.500000000	0.500000000	0.055084293
0.250000000	0.666666705	0.055084293
0.749999979	0.666666705	0.055084293
0.000000000	0.833333295	0.055084293
0.500000000	0.833333295	0.055084293
0.254544705	0.999397867	0.164984662
0.754386966	0.999897899	0.164331450
0.002437824	0.166243321	0.164636176
0.502233344	0.165994227	0.164763900

0.251700350	0.331939791	0.164847333
0.753099931	0.330564790	0.164481572
0.004765246	0.498580999	0.164107473
0.505044384	0.497992622	0.164685631
0.252543607	0.667207543	0.166152409
0.753379732	0.666359308	0.165599319
0.000499611	0.834903203	0.165673283
0.500664464	0.835252327	0.166086769

OER_LSC/MoSe2_bare
1.0

11.1999998093	0.0000000000	0.0000000000
0.0000000000	16.5699996948	0.0000000000
0.0000000000	0.0000000000	35.0000000000

Mo	Se	La	Sr	Co	O
8	16	18	18	24	84

Direct

0.996218000	-0.000000000	0.365928841
0.496218000	-0.000000000	0.365928841
0.259126046	0.093587013	0.365618951
0.759126046	0.093587013	0.365618951
0.991637298	0.188748712	0.364897455
0.491637298	0.188748712	0.364897455
0.242296930	0.311316569	0.364960235
0.742296930	0.311316569	0.364960235
0.334845042	-0.000000000	0.419420569
0.834845063	-0.000000000	0.419420569
0.085303173	0.098975791	0.416193526
0.585303189	0.098975791	0.416193526
0.327459877	0.202735005	0.413834626
0.827459877	0.202735005	0.413834626
0.077551035	0.297099167	0.413750567
0.577551009	0.297099167	0.413750567
0.166621552	-0.000000000	0.314193453
0.666621552	-0.000000000	0.314193453
0.418432107	0.098376047	0.312279483
0.918432149	0.098376047	0.312279483
0.168383241	0.199207950	0.317593602
0.668383252	0.199207950	0.317593602
0.418405710	0.300765633	0.317174230
0.918405753	0.300765633	0.317174230
0.000000000	0.000000000	0.000000000
0.500000000	0.000000000	0.000000000
0.000000000	0.333333353	0.000000000
0.500000000	0.333333353	0.000000000
0.000000000	0.666666705	0.000000000
0.500000000	0.666666705	0.000000000
0.249917852	0.166346660	0.107885224
0.749917895	0.166346660	0.107885224

0.249908911	0.500000000	0.106344155
0.749908954	0.500000000	0.106344155
0.249917852	0.833653354	0.107885224
0.749917895	0.833653354	0.107885224
0.988801394	0.009313737	0.212395627
0.488801351	0.009313737	0.212395627
0.979886668	0.335834517	0.213063608
0.479886668	0.335834517	0.213063608
0.984607356	0.677515398	0.210469818
0.484607356	0.677515398	0.210469818
0.250000000	0.166666676	0.000000000
0.749999979	0.166666676	0.000000000
0.250000000	0.500000000	0.000000000
0.749999979	0.500000000	0.000000000
0.250000000	0.833333295	0.000000000
0.749999979	0.833333295	0.000000000
0.999390074	0.000000000	0.107119492
0.499390074	0.000000000	0.107119492
0.999982970	0.332969955	0.107268674
0.499982970	0.332969955	0.107268674
0.999982970	0.667030045	0.107268674
0.499982970	0.667030045	0.107268674
0.240204253	0.171369379	0.217509515
0.740204211	0.171369379	0.217509515
0.242813532	0.500742738	0.214207336
0.742813532	0.500742738	0.214207336
0.243907681	0.839982080	0.215488189
0.743907703	0.839982080	0.215488189
0.250000000	-0.000000000	0.055084293
0.749999979	-0.000000000	0.055084293
-0.000000000	0.166666676	0.055084293
0.500000000	0.166666676	0.055084293
0.250000000	0.333333353	0.055084293
0.749999979	0.333333353	0.055084293
0.000000000	0.500000000	0.055084293
0.500000000	0.500000000	0.055084293
0.250000000	0.666666705	0.055084293
0.749999979	0.666666705	0.055084293
0.000000000	0.833333295	0.055084293
0.500000000	0.833333295	0.055084293
0.247895828	0.003018569	0.164762987
0.747895850	0.003018569	0.164762987
0.997040033	0.167342910	0.164440264
0.497039991	0.167342910	0.164440264
0.245313265	0.334268609	0.164734432
0.745313265	0.334268609	0.164734432
0.997305359	0.500092547	0.165418448
0.497305359	0.500092547	0.165418448
0.244843730	0.668465391	0.165356840

0.744843751	0.668465391	0.165356840
0.997233663	0.835482487	0.165156542
0.497233663	0.835482487	0.165156542
0.250000000	0.000000000	0.000000000
0.749999979	0.000000000	0.000000000
0.000000000	0.166666676	0.000000000
0.500000000	0.166666676	0.000000000
0.250000000	0.333333353	0.000000000
0.749999979	0.333333353	0.000000000
0.000000000	0.500000000	0.000000000
0.500000000	0.500000000	0.000000000
0.250000000	0.666666705	0.000000000
0.749999979	0.666666705	0.000000000
0.000000000	0.833333295	0.000000000
0.500000000	0.833333295	0.000000000
0.125000000	0.083333338	0.055134330
0.374999989	0.083333338	0.055134330
0.625000011	0.083333338	0.055134330
0.875000032	0.083333338	0.055134330
0.125000000	0.250000000	0.055134330
0.374999989	0.250000000	0.055134330
0.625000011	0.250000000	0.055134330
0.875000032	0.250000000	0.055134330
0.125000000	0.416666647	0.055134330
0.374999989	0.416666647	0.055134330
0.625000011	0.416666647	0.055134330
0.875000032	0.416666647	0.055134330
0.125000000	0.583333295	0.055134330
0.374999989	0.583333295	0.055134330
0.625000011	0.583333295	0.055134330
0.875000032	0.583333295	0.055134330
0.125000000	0.750000000	0.055134330
0.374999989	0.750000000	0.055134330
0.625000011	0.750000000	0.055134330
0.875000032	0.750000000	0.055134330
0.125000000	0.916666705	0.055134330
0.374999989	0.916666705	0.055134330
0.625000011	0.916666705	0.055134330
0.875000032	0.916666705	0.055134330
0.249987057	-0.000000000	0.110441978
0.749987036	-0.000000000	0.110441978
0.000314729	0.167628149	0.110136305
0.500314713	0.167628149	0.110136305
0.250063713	0.329844902	0.110218402
0.750063756	0.329844902	0.110218402
0.999881898	0.500000000	0.109866755
0.499881940	0.500000000	0.109866755
0.250063713	0.670155127	0.110218402
0.750063756	0.670155127	0.110218402

0.000314729	0.832371851	0.110136305
0.500314713	0.832371851	0.110136305
0.142107811	0.100141143	0.164001315
0.398139194	0.072638078	0.161667606
0.642107864	0.100141143	0.164001315
0.898139237	0.072638078	0.161667606
0.096050524	0.263261541	0.168514633
0.354805382	0.233097863	0.164828995
0.596050503	0.263261541	0.168514633
0.854805382	0.233097863	0.164828995
0.148136927	0.433214624	0.161151614
0.394700091	0.402038279	0.162553992
0.648136916	0.433214624	0.161151614
0.894700134	0.402038279	0.162553992
0.097813842	0.601295351	0.168320956
0.351085392	0.570295870	0.163424015
0.597813863	0.601295351	0.168320956
0.851085371	0.570295870	0.163424015
0.138092799	0.768493800	0.164086846
0.393952862	0.742106028	0.161535113
0.638092810	0.768493800	0.164086846
0.893952862	0.742106028	0.161535113
0.098966000	0.931395535	0.168318422
0.357815715	0.901199915	0.162547316
0.598966021	0.931395535	0.168318422
0.857815757	0.901199915	0.162547316
0.269468367	0.001646377	0.217819718
0.769468389	0.001646377	0.217819718
0.994029658	0.157636655	0.218823460
0.494029616	0.157636655	0.218823460
0.259496212	0.336216908	0.218980994
0.759496191	0.336216908	0.218980994
0.997411711	0.486740991	0.220005240
0.497411711	0.486740991	0.220005240
0.264383218	0.673576499	0.219738688
0.764383261	0.673576499	0.219738688
0.992229938	0.827303449	0.219788170
0.492229938	0.827303449	0.219788170

OER_LSC/MoSe2_OH
1.0

11.1999998093	0.0000000000	0.0000000000
0.0000000000	16.5699996948	0.0000000000
0.0000000000	0.0000000000	35.0000000000

H	O	Mo	Se	La	Sr	Co
1	85	8	16	18	18	24

Direct

0.521702988	0.663106973	0.304130309
0.516239711	0.662093960	0.276508713

0.250000000	0.000000000	0.000000000
0.749999979	0.000000000	0.000000000
0.000000000	0.166666676	0.000000000
0.500000000	0.166666676	0.000000000
0.250000000	0.333333353	0.000000000
0.749999979	0.333333353	0.000000000
0.000000000	0.500000000	0.000000000
0.500000000	0.500000000	0.000000000
0.250000000	0.666666705	0.000000000
0.749999979	0.666666705	0.000000000
0.000000000	0.833333295	0.000000000
0.500000000	0.833333295	0.000000000
0.125000000	0.083333338	0.055134330
0.374999989	0.083333338	0.055134330
0.625000011	0.083333338	0.055134330
0.875000032	0.083333338	0.055134330
0.125000000	0.250000000	0.055134330
0.374999989	0.250000000	0.055134330
0.625000011	0.250000000	0.055134330
0.875000032	0.250000000	0.055134330
0.125000000	0.416666647	0.055134330
0.374999989	0.416666647	0.055134330
0.625000011	0.416666647	0.055134330
0.875000032	0.416666647	0.055134330
0.125000000	0.583333295	0.055134330
0.374999989	0.583333295	0.055134330
0.625000011	0.583333295	0.055134330
0.875000032	0.583333295	0.055134330
0.125000000	0.750000000	0.055134330
0.374999989	0.750000000	0.055134330
0.625000011	0.750000000	0.055134330
0.875000032	0.750000000	0.055134330
0.125000000	0.916666705	0.055134330
0.374999989	0.916666705	0.055134330
0.625000011	0.916666705	0.055134330
0.875000032	0.916666705	0.055134330
0.249987057	-0.000000000	0.110441978
0.749987036	-0.000000000	0.110441978
0.000314729	0.167628149	0.110136305
0.500314713	0.167628149	0.110136305
0.250063713	0.329844902	0.110218402
0.750063756	0.329844902	0.110218402
0.999881898	0.500000000	0.109866755
0.499881940	0.500000000	0.109866755
0.250063713	0.670155127	0.110218402
0.750063756	0.670155127	0.110218402
0.000314729	0.832371851	0.110136305
0.500314713	0.832371851	0.110136305
0.141568633	0.099869926	0.163581944

0.397142902	0.073450262	0.161888899
0.641214135	0.099404981	0.163791983
0.897016455	0.072788906	0.161829117
0.095974123	0.263724824	0.168752711
0.355258462	0.234091466	0.164990112
0.596671285	0.263491902	0.168373694
0.855916668	0.233737421	0.165036474
0.148989795	0.434237219	0.161019461
0.394893508	0.403466143	0.161663560
0.649011785	0.434219406	0.160959026
0.895414623	0.403756562	0.162258284
0.103776361	0.598968086	0.166684791
0.356410599	0.572410242	0.163388048
0.604079802	0.597208882	0.168931212
0.855350679	0.572886734	0.162102604
0.135025140	0.766535976	0.163746371
0.385362257	0.745722624	0.161795398
0.639683189	0.765556805	0.164630154
0.892820629	0.742985573	0.161067186
0.100377151	0.930710063	0.167667007
0.359381784	0.905256743	0.161638996
0.601367492	0.929883756	0.166529478
0.858860116	0.902602742	0.162846933
0.267773190	0.001687817	0.218031447
0.765958782	0.000911101	0.217647226
0.994679349	0.156204000	0.218878174
0.494341476	0.157400582	0.218789346
0.259635602	0.337190525	0.218735600
0.760902690	0.336392765	0.218768651
0.998776828	0.489519827	0.219017696
0.496799699	0.483382412	0.218342113
0.252388805	0.671322961	0.219103541
0.774883897	0.670320596	0.218703706
0.992329648	0.828920781	0.219657271
0.489848009	0.835695495	0.218783351
0.996218000	-0.000000000	0.365928841
0.496218000	-0.000000000	0.365928841
0.259126046	0.093587013	0.365618951
0.759126046	0.093587013	0.365618951
0.991637298	0.188748712	0.364897455
0.491637298	0.188748712	0.364897455
0.242296930	0.311316569	0.364960235
0.742296930	0.311316569	0.364960235
0.334845042	-0.000000000	0.419420569
0.834845063	-0.000000000	0.419420569
0.085303173	0.098975791	0.416193526
0.585303189	0.098975791	0.416193526
0.327459877	0.202735005	0.413834626
0.827459877	0.202735005	0.413834626

0.077551035	0.297099167	0.413750567
0.577551009	0.297099167	0.413750567
0.166621552	-0.000000000	0.314193453
0.666621552	-0.000000000	0.314193453
0.418432107	0.098376047	0.312279483
0.918432149	0.098376047	0.312279483
0.168383241	0.199207950	0.317593602
0.668383252	0.199207950	0.317593602
0.418405710	0.300765633	0.317174230
0.918405753	0.300765633	0.317174230
0.000000000	0.000000000	0.000000000
0.500000000	0.000000000	0.000000000
0.000000000	0.333333353	0.000000000
0.500000000	0.333333353	0.000000000
0.000000000	0.666666705	0.000000000
0.500000000	0.666666705	0.000000000
0.249917852	0.166346660	0.107885224
0.749917895	0.166346660	0.107885224
0.249908911	0.500000000	0.106344155
0.749908954	0.500000000	0.106344155
0.249917852	0.833653354	0.107885224
0.749917895	0.833653354	0.107885224
0.986705354	0.006166148	0.211898899
0.490567650	0.004966224	0.211458751
0.979147826	0.336801516	0.212724331
0.478974291	0.335342112	0.212605899
0.991879889	0.678190395	0.209906687
0.484449957	0.677359714	0.214970602
0.250000000	0.166666676	0.000000000
0.749999979	0.166666676	0.000000000
0.250000000	0.500000000	0.000000000
0.749999979	0.500000000	0.000000000
0.250000000	0.833333295	0.000000000
0.749999979	0.833333295	0.000000000
0.999390074	0.000000000	0.107119492
0.499390074	0.000000000	0.107119492
0.999982970	0.332969955	0.107268674
0.499982970	0.332969955	0.107268674
0.999982970	0.667030045	0.107268674
0.499982970	0.667030045	0.107268674
0.240076933	0.170629475	0.217267159
0.740021735	0.170394668	0.217136928
0.244016013	0.503945115	0.214396068
0.739993721	0.508663760	0.214622974
0.247277515	0.836921631	0.214444201
0.746309004	0.835252557	0.215558747
0.250000000	-0.000000000	0.055084293
0.749999979	-0.000000000	0.055084293
-0.000000000	0.166666676	0.055084293

0.500000000	0.166666676	0.055084293
0.250000000	0.333333353	0.055084293
0.749999979	0.333333353	0.055084293
0.000000000	0.500000000	0.055084293
0.500000000	0.500000000	0.055084293
0.250000000	0.666666705	0.055084293
0.749999979	0.666666705	0.055084293
0.000000000	0.833333295	0.055084293
0.500000000	0.833333295	0.055084293
0.247448985	0.003188526	0.165659128
0.747641678	0.002303782	0.164558234
0.996759125	0.167281860	0.164777633
0.496953053	0.167186722	0.164763383
0.245205828	0.334998513	0.164809023
0.745016094	0.335429882	0.164976297
0.998790111	0.500914307	0.165167713
0.498896420	0.500663025	0.165391541
0.247789434	0.668547924	0.165140016
0.748912960	0.668323002	0.165184471
0.997523427	0.835612905	0.164922428
0.496922399	0.835524847	0.165056161

OER_LSC/MoSe2_Vo_OO
1.0

11.1999998093	0.0000000000	0.0000000000
0.0000000000	16.5699996948	0.0000000000
0.0000000000	0.0000000000	35.0000000000

O	Mo	Se	La	Sr	Co
85	8	16	18	18	24

Direct

0.242329180	0.648113797	0.223567309
0.310668461	0.704029962	0.248316356
0.250000000	0.000000000	0.000000000
0.749999979	0.000000000	0.000000000
0.000000000	0.166666676	0.000000000
0.500000000	0.166666676	0.000000000
0.250000000	0.333333353	0.000000000
0.749999979	0.333333353	0.000000000
0.000000000	0.500000000	0.000000000
0.500000000	0.500000000	0.000000000
0.250000000	0.666666705	0.000000000
0.749999979	0.666666705	0.000000000
0.000000000	0.833333295	0.000000000
0.500000000	0.833333295	0.000000000
0.125000000	0.083333338	0.055134330
0.374999989	0.083333338	0.055134330
0.625000011	0.083333338	0.055134330
0.875000032	0.083333338	0.055134330
0.125000000	0.250000000	0.055134330

0.374999989	0.250000000	0.055134330
0.625000011	0.250000000	0.055134330
0.875000032	0.250000000	0.055134330
0.125000000	0.416666647	0.055134330
0.374999989	0.416666647	0.055134330
0.625000011	0.416666647	0.055134330
0.875000032	0.416666647	0.055134330
0.125000000	0.583333295	0.055134330
0.374999989	0.583333295	0.055134330
0.625000011	0.583333295	0.055134330
0.875000032	0.583333295	0.055134330
0.125000000	0.750000000	0.055134330
0.374999989	0.750000000	0.055134330
0.625000011	0.750000000	0.055134330
0.875000032	0.750000000	0.055134330
0.125000000	0.916666705	0.055134330
0.374999989	0.916666705	0.055134330
0.625000011	0.916666705	0.055134330
0.875000032	0.916666705	0.055134330
0.249987057	-0.000000000	0.110441978
0.749987036	-0.000000000	0.110441978
0.000314729	0.167628149	0.110136305
0.500314713	0.167628149	0.110136305
0.250063713	0.329844902	0.110218402
0.750063756	0.329844902	0.110218402
0.999881898	0.500000000	0.109866755
0.499881940	0.500000000	0.109866755
0.250063713	0.670155127	0.110218402
0.750063756	0.670155127	0.110218402
0.000314729	0.832371851	0.110136305
0.500314713	0.832371851	0.110136305
0.098558155	0.070063481	0.167621885
0.357511390	0.099415218	0.163584764
0.599095576	0.070139049	0.167469624
0.857708724	0.098827553	0.163822842
0.141725702	0.232761617	0.163961288
0.397152141	0.260207510	0.162515545
0.641539576	0.232662451	0.163818741
0.896257433	0.259687679	0.162418188
0.098458200	0.402832844	0.168415478
0.355827878	0.432148086	0.161462239
0.597813310	0.401741414	0.167610332
0.855412327	0.430247097	0.162200424
0.134833266	0.570526836	0.164493711
0.390638035	0.592171272	0.161962863
0.637718365	0.566412568	0.162106160
0.891097970	0.591750435	0.161419896
0.101125838	0.739577669	0.168982247
0.360210970	0.761387171	0.167749282

0.599826883	0.738215359	0.168733760
0.859926187	0.763776191	0.164981515
0.141640276	0.900912143	0.163261059
0.396144354	0.926647767	0.162437480
0.644372287	0.900884690	0.161798682
0.897391539	0.926809840	0.161484337
0.265899986	0.998546639	0.218028055
0.765556451	0.996711349	0.217696190
0.995066013	0.175416768	0.218557426
0.495953781	0.174795987	0.218522549
0.266072606	0.332330613	0.218032156
0.763116577	0.331285025	0.218223218
0.990151695	0.507734719	0.219562476
0.492255270	0.505472145	0.219031511
0.759369233	0.661020400	0.218943923
0.994263053	0.845746083	0.220081438
0.503204686	0.851277164	0.219919068
0.996218000	-0.000000000	0.365928841
0.496218000	-0.000000000	0.365928841
0.259126046	0.093587013	0.365618951
0.759126046	0.093587013	0.365618951
0.991637298	0.188748712	0.364897455
0.491637298	0.188748712	0.364897455
0.242296930	0.311316569	0.364960235
0.742296930	0.311316569	0.364960235
0.334845042	-0.000000000	0.419420569
0.834845063	-0.000000000	0.419420569
0.085303173	0.098975791	0.416193526
0.585303189	0.098975791	0.416193526
0.327459877	0.202735005	0.413834626
0.827459877	0.202735005	0.413834626
0.077551035	0.297099167	0.413750567
0.577551009	0.297099167	0.413750567
0.166621552	-0.000000000	0.314193453
0.666621552	-0.000000000	0.314193453
0.418432107	0.098376047	0.312279483
0.918432149	0.098376047	0.312279483
0.168383241	0.199207950	0.317593602
0.668383252	0.199207950	0.317593602
0.418405710	0.300765633	0.317174230
0.918405753	0.300765633	0.317174230
0.000000000	0.000000000	0.000000000
0.500000000	0.000000000	0.000000000
0.000000000	0.333333353	0.000000000
0.500000000	0.333333353	0.000000000
0.000000000	0.666666705	0.000000000
0.500000000	0.666666705	0.000000000
0.249917852	0.166346660	0.107885224
0.749917895	0.166346660	0.107885224

0.249908911	0.500000000	0.106344155
0.749908954	0.500000000	0.106344155
0.249917852	0.833653354	0.107885224
0.749917895	0.833653354	0.107885224
0.986516152	0.994604172	0.211803763
0.489112403	0.996054654	0.212275246
0.983750326	0.325300330	0.212403515
0.487494392	0.325388704	0.212450368
0.986972298	0.657664065	0.209939057
0.481852216	0.660501606	0.211549963
0.250000000	0.166666676	0.000000000
0.749999979	0.166666676	0.000000000
0.250000000	0.500000000	0.000000000
0.749999979	0.500000000	0.000000000
0.250000000	0.833333295	0.000000000
0.749999979	0.833333295	0.000000000
0.999390074	0.000000000	0.107119492
0.499390074	0.000000000	0.107119492
0.999982970	0.332969955	0.107268674
0.499982970	0.332969955	0.107268674
0.999982970	0.667030045	0.107268674
0.499982970	0.667030045	0.107268674
0.243523189	0.162942598	0.216942828
0.743525126	0.163028196	0.216814245
0.241038016	0.491361909	0.214205687
0.743972927	0.496371951	0.213663428
0.250215641	0.837847392	0.217948151
0.743117090	0.832309750	0.215304157
0.250000000	-0.000000000	0.055084293
0.749999979	-0.000000000	0.055084293
-0.000000000	0.166666676	0.055084293
0.500000000	0.166666676	0.055084293
0.250000000	0.333333353	0.055084293
0.749999979	0.333333353	0.055084293
0.000000000	0.500000000	0.055084293
0.500000000	0.500000000	0.055084293
0.250000000	0.666666705	0.055084293
0.749999979	0.666666705	0.055084293
0.000000000	0.833333295	0.055084293
0.500000000	0.833333295	0.055084293
0.247612621	0.999455421	0.164797361
0.747303975	0.998974037	0.164548697
0.998000520	0.166138443	0.164438343
0.498105594	0.166225451	0.164367008
0.246334144	0.330617999	0.164642470
0.745391944	0.331212564	0.164602539
0.995725495	0.498426696	0.164409092
0.495887152	0.499143592	0.164905821
0.245461426	0.666884490	0.164863913

0.744333535	0.665698930	0.165271923
0.999921407	0.833764376	0.165886102
0.499291854	0.833876147	0.165295301

OER_LSC/MoSe2_Vo_OH
LSC
1.0

11.1999998093	0.0000000000	0.0000000000
0.0000000000	16.5699996948	0.0000000000
0.0000000000	0.0000000000	35.0000000000

H	O	Mo	Se	La	Sr	Co
1	84	8	16	18	18	24

Direct

0.273383711	0.671832316	0.267754800
0.285138003	0.672513529	0.240139389
0.250000000	0.000000000	0.000000000
0.749999979	0.000000000	0.000000000
0.000000000	0.166666676	0.000000000
0.500000000	0.166666676	0.000000000
0.250000000	0.333333353	0.000000000
0.749999979	0.333333353	0.000000000
0.000000000	0.500000000	0.000000000
0.500000000	0.500000000	0.000000000
0.250000000	0.666666705	0.000000000
0.749999979	0.666666705	0.000000000
0.000000000	0.833333295	0.000000000
0.500000000	0.833333295	0.000000000
0.125000000	0.083333338	0.055134330
0.374999989	0.083333338	0.055134330
0.625000011	0.083333338	0.055134330
0.875000032	0.083333338	0.055134330
0.125000000	0.250000000	0.055134330
0.374999989	0.250000000	0.055134330
0.625000011	0.250000000	0.055134330
0.875000032	0.250000000	0.055134330
0.125000000	0.416666647	0.055134330
0.374999989	0.416666647	0.055134330
0.625000011	0.416666647	0.055134330
0.875000032	0.416666647	0.055134330
0.125000000	0.583333295	0.055134330
0.374999989	0.583333295	0.055134330
0.625000011	0.583333295	0.055134330
0.875000032	0.583333295	0.055134330
0.125000000	0.750000000	0.055134330
0.374999989	0.750000000	0.055134330
0.625000011	0.750000000	0.055134330
0.875000032	0.750000000	0.055134330
0.125000000	0.916666705	0.055134330
0.374999989	0.916666705	0.055134330

0.625000011	0.916666705	0.055134330
0.875000032	0.916666705	0.055134330
0.249987057	-0.000000000	0.110441978
0.749987036	-0.000000000	0.110441978
0.000314729	0.167628149	0.110136305
0.500314713	0.167628149	0.110136305
0.250063713	0.329844902	0.110218402
0.750063756	0.329844902	0.110218402
0.999881898	0.500000000	0.109866755
0.499881940	0.500000000	0.109866755
0.250063713	0.670155127	0.110218402
0.750063756	0.670155127	0.110218402
0.000314729	0.832371851	0.110136305
0.500314713	0.832371851	0.110136305
0.139322038	0.098146535	0.164143835
0.397569374	0.073528154	0.162069838
0.641486400	0.100839334	0.163796806
0.895654149	0.074074251	0.161569868
0.098041968	0.262459090	0.168076570
0.357592240	0.234383986	0.165190588
0.596787982	0.263437254	0.168386337
0.857679348	0.235396265	0.164657130
0.142661814	0.434029161	0.161405359
0.391665917	0.406392490	0.162696798
0.648696774	0.433103688	0.160090515
0.896274463	0.407296927	0.162529741
0.103733073	0.595631377	0.171592208
0.359436961	0.577065750	0.169114017
0.603398563	0.597573201	0.167137037
0.863413485	0.573306765	0.165434524
0.134946227	0.760529957	0.168138327
0.388118759	0.742145395	0.166060025
0.635385056	0.765875656	0.164835916
0.889826347	0.743566353	0.161850861
0.101058868	0.927966393	0.168530246
0.357389456	0.902152092	0.162729468
0.600485088	0.931308800	0.167370088
0.859896896	0.904102204	0.162996605
0.269064078	0.001601614	0.217846067
0.766217977	0.003248156	0.217720195
0.994020377	0.157621403	0.218659810
0.495117528	0.158497984	0.218887125
0.257522170	0.337033114	0.218223994
0.761839420	0.337365720	0.217855522
0.998994470	0.484780866	0.220133196
0.505599601	0.482744969	0.219936030
0.756941876	0.672516119	0.219494615
0.987637486	0.826468797	0.220011234
0.496317029	0.831867675	0.220287064

0.996218000	-0.000000000	0.365928841
0.496218000	-0.000000000	0.365928841
0.259126046	0.093587013	0.365618951
0.759126046	0.093587013	0.365618951
0.991637298	0.188748712	0.364897455
0.491637298	0.188748712	0.364897455
0.242296930	0.311316569	0.364960235
0.742296930	0.311316569	0.364960235
0.334845042	-0.000000000	0.419420569
0.834845063	-0.000000000	0.419420569
0.085303173	0.098975791	0.416193526
0.585303189	0.098975791	0.416193526
0.327459877	0.202735005	0.413834626
0.827459877	0.202735005	0.413834626
0.077551035	0.297099167	0.413750567
0.577551009	0.297099167	0.413750567
0.166621552	-0.000000000	0.314193453
0.666621552	-0.000000000	0.314193453
0.418432107	0.098376047	0.312279483
0.918432149	0.098376047	0.312279483
0.168383241	0.199207950	0.317593602
0.668383252	0.199207950	0.317593602
0.418405710	0.300765633	0.317174230
0.918405753	0.300765633	0.317174230
0.000000000	0.000000000	0.000000000
0.500000000	0.000000000	0.000000000
0.000000000	0.333333353	0.000000000
0.500000000	0.333333353	0.000000000
0.000000000	0.666666705	0.000000000
0.500000000	0.666666705	0.000000000
0.249917852	0.166346660	0.107885224
0.749917895	0.166346660	0.107885224
0.249908911	0.500000000	0.106344155
0.749908954	0.500000000	0.106344155
0.249917852	0.833653354	0.107885224
0.749917895	0.833653354	0.107885224
0.987586992	0.008294918	0.211362948
0.490181838	0.008106343	0.211994171
0.984612890	0.336946869	0.212402303
0.477820771	0.334224696	0.212648705
0.979513713	0.676077117	0.210198089
0.500063777	0.678300784	0.210956819
0.250000000	0.166666676	0.000000000
0.749999979	0.166666676	0.000000000
0.250000000	0.500000000	0.000000000
0.749999979	0.500000000	0.000000000
0.250000000	0.833333295	0.000000000
0.749999979	0.833333295	0.000000000
0.999390074	0.000000000	0.107119492

0.499390074	0.000000000	0.107119492
0.999982970	0.332969955	0.107268674
0.499982970	0.332969955	0.107268674
0.999982970	0.667030045	0.107268674
0.499982970	0.667030045	0.107268674
0.240339811	0.169894406	0.216869504
0.742265680	0.171434098	0.216960226
0.242914391	0.498288739	0.214966556
0.747347061	0.497934607	0.213688919
0.248066170	0.839260177	0.215732847
0.743875942	0.841640564	0.215088858
0.250000000	-0.000000000	0.055084293
0.749999979	-0.000000000	0.055084293
-0.000000000	0.166666676	0.055084293
0.500000000	0.166666676	0.055084293
0.250000000	0.333333353	0.055084293
0.749999979	0.333333353	0.055084293
0.000000000	0.500000000	0.055084293
0.500000000	0.500000000	0.055084293
0.250000000	0.666666705	0.055084293
0.749999979	0.666666705	0.055084293
0.000000000	0.833333295	0.055084293
0.500000000	0.833333295	0.055084293
0.248598375	0.002008060	0.164678832
0.746903603	0.003460321	0.164377826
0.996906093	0.167455573	0.164383629
0.497629566	0.167548163	0.164548656
0.244596349	0.334157530	0.163383906
0.746030905	0.334672468	0.164284761
0.000782937	0.500026590	0.165059553
0.499285638	0.500233728	0.165876443
0.244759964	0.666384229	0.164430959
0.745879424	0.668510571	0.164403984
0.996621949	0.834970023	0.165209021
0.496066945	0.835811352	0.165072714

OER_LSC/MoSe2-Ho_OH
1.0

11.1999998093	0.0000000000	0.0000000000
0.0000000000	16.5699996948	0.0000000000
0.0000000000	0.0000000000	35.0000000000

H	O	Mo	Se	La	Sr	Co
2	85	8	16	18	18	24

Direct

0.274333469	0.665586757	0.246380915
0.396762497	0.668389592	0.295253699
0.403484326	0.666743655	0.267632021
0.250000000	0.000000000	0.000000000
0.749999979	0.000000000	0.000000000

0.000000000	0.166666676	0.000000000
0.500000000	0.166666676	0.000000000
0.250000000	0.333333353	0.000000000
0.749999979	0.333333353	0.000000000
0.000000000	0.500000000	0.000000000
0.500000000	0.500000000	0.000000000
0.250000000	0.666666705	0.000000000
0.749999979	0.666666705	0.000000000
0.000000000	0.833333295	0.000000000
0.500000000	0.833333295	0.000000000
0.125000000	0.083333338	0.055134330
0.374999989	0.083333338	0.055134330
0.625000011	0.083333338	0.055134330
0.875000032	0.083333338	0.055134330
0.125000000	0.250000000	0.055134330
0.374999989	0.250000000	0.055134330
0.625000011	0.250000000	0.055134330
0.875000032	0.250000000	0.055134330
0.125000000	0.416666647	0.055134330
0.374999989	0.416666647	0.055134330
0.625000011	0.416666647	0.055134330
0.875000032	0.416666647	0.055134330
0.125000000	0.583333295	0.055134330
0.374999989	0.583333295	0.055134330
0.625000011	0.583333295	0.055134330
0.875000032	0.583333295	0.055134330
0.125000000	0.750000000	0.055134330
0.374999989	0.750000000	0.055134330
0.625000011	0.750000000	0.055134330
0.875000032	0.750000000	0.055134330
0.125000000	0.916666705	0.055134330
0.374999989	0.916666705	0.055134330
0.625000011	0.916666705	0.055134330
0.875000032	0.916666705	0.055134330
0.249987057	-0.000000000	0.110441978
0.749987036	-0.000000000	0.110441978
0.000314729	0.167628149	0.110136305
0.500314713	0.167628149	0.110136305
0.250063713	0.329844902	0.110218402
0.750063756	0.329844902	0.110218402
0.999881898	0.500000000	0.109866755
0.499881940	0.500000000	0.109866755
0.250063713	0.670155127	0.110218402
0.750063756	0.670155127	0.110218402
0.000314729	0.832371851	0.110136305
0.500314713	0.832371851	0.110136305
0.144048197	0.099153483	0.164338521
0.402962828	0.074039704	0.164073808
0.646110699	0.100992328	0.164561422

0.901886751	0.072298975	0.164256859
0.100974996	0.260907772	0.163306672
0.356369046	0.234442619	0.164772633
0.599291377	0.260316287	0.163206237
0.857900651	0.234016401	0.164725331
0.145236959	0.432404029	0.160871179
0.399928738	0.402864068	0.167126901
0.642989927	0.429039982	0.159940202
0.900526914	0.402225244	0.167130743
0.108292903	0.591785313	0.163537666
0.368152522	0.569106108	0.167032787
0.613168999	0.587784773	0.161140292
0.865270084	0.569218339	0.163058662
0.137203934	0.766662826	0.165284688
0.392990672	0.736051204	0.172819628
0.645026320	0.763488190	0.165123762
0.898727620	0.738873953	0.166355228
0.100074710	0.930931417	0.165573147
0.350007357	0.900682156	0.160907473
0.606365384	0.934361019	0.164312458
0.858930705	0.900975683	0.161122118
0.267234960	0.000970994	0.217809405
0.769170365	0.999609897	0.218194444
0.002298047	0.170301243	0.218639483
0.502840289	0.170361359	0.218632208
0.229610890	0.335144183	0.218466377
0.731972894	0.333248777	0.217956638
0.005318887	0.503345860	0.218950244
0.517814935	0.495043483	0.218706744
0.204909639	0.663394284	0.227797331
0.749470264	0.660256540	0.219137791
0.993362597	0.845215145	0.219833306
0.500241654	0.854921731	0.220122024
0.996218000	-0.000000000	0.365928841
0.496218000	-0.000000000	0.365928841
0.259126046	0.093587013	0.365618951
0.759126046	0.093587013	0.365618951
0.991637298	0.188748712	0.364897455
0.491637298	0.188748712	0.364897455
0.242296930	0.311316569	0.364960235
0.742296930	0.311316569	0.364960235
0.334845042	-0.000000000	0.419420569
0.834845063	-0.000000000	0.419420569
0.085303173	0.098975791	0.416193526
0.585303189	0.098975791	0.416193526
0.327459877	0.202735005	0.413834626
0.827459877	0.202735005	0.413834626
0.077551035	0.297099167	0.413750567
0.577551009	0.297099167	0.413750567

0.166621552	-0.000000000	0.314193453
0.666621552	-0.000000000	0.314193453
0.418432107	0.098376047	0.312279483
0.918432149	0.098376047	0.312279483
0.168383241	0.199207950	0.317593602
0.668383252	0.199207950	0.317593602
0.418405710	0.300765633	0.317174230
0.918405753	0.300765633	0.317174230
0.000000000	0.000000000	0.000000000
0.500000000	0.000000000	0.000000000
0.000000000	0.333333353	0.000000000
0.500000000	0.333333353	0.000000000
0.000000000	0.666666705	0.000000000
0.500000000	0.666666705	0.000000000
0.249917852	0.166346660	0.107885224
0.749917895	0.166346660	0.107885224
0.249908911	0.500000000	0.106344155
0.749908954	0.500000000	0.106344155
0.249917852	0.833653354	0.107885224
0.749917895	0.833653354	0.107885224
0.996068988	0.999381061	0.212259483
0.500808409	0.000704838	0.211985779
0.008215337	0.326278062	0.212316036
0.509817013	0.331340219	0.212364319
0.994960427	0.657345675	0.210588346
0.518709643	0.655965524	0.212476635
0.250000000	0.166666676	0.000000000
0.749999979	0.166666676	0.000000000
0.250000000	0.500000000	0.000000000
0.749999979	0.500000000	0.000000000
0.250000000	0.833333295	0.000000000
0.749999979	0.833333295	0.000000000
0.999390074	0.000000000	0.107119492
0.499390074	0.000000000	0.107119492
0.999982970	0.332969955	0.107268674
0.499982970	0.332969955	0.107268674
0.999982970	0.667030045	0.107268674
0.499982970	0.667030045	0.107268674
0.252938786	0.164728723	0.217305224
0.751984132	0.165833190	0.217700454
0.246101367	0.497581282	0.214670427
0.755548064	0.494121464	0.212775612
0.253996619	0.833960579	0.216009508
0.754421366	0.836765371	0.214861652
0.250000000	-0.000000000	0.055084293
0.749999979	-0.000000000	0.055084293
-0.000000000	0.166666676	0.055084293
0.500000000	0.166666676	0.055084293
0.250000000	0.333333353	0.055084293

0.749999979	0.333333353	0.055084293
0.000000000	0.500000000	0.055084293
0.500000000	0.500000000	0.055084293
0.250000000	0.666666705	0.055084293
0.749999979	0.666666705	0.055084293
0.000000000	0.833333295	0.055084293
0.500000000	0.833333295	0.055084293
0.249623171	0.999577897	0.165061705
0.750962338	0.002084207	0.164848791
0.001352758	0.166383380	0.164513275
0.501062623	0.167266046	0.165140002
0.251386255	0.332171015	0.165037741
0.752201945	0.330962116	0.164392539
0.003611102	0.498398034	0.163813414
0.504143162	0.497230431	0.164965030
0.249109209	0.666837641	0.166466618
0.753084945	0.666492028	0.164497239
0.000122209	0.834745447	0.166023472
0.499600521	0.833637469	0.167013495

Supplementary References

1. Qian, Z. et al. Hollow nanocages of $Ni_xCo_{1-x}Se$ for efficient zinc-air batteries and overall water splitting. *Nano-Micro Lett.* **11**, 1-17 (2019).
2. Sancho, Hugo et al. $NiCo_2Se_4$ Nanowires as a High-Performance Bifunctional Oxygen Electrocatalyst. *J. Electrochem. Soc.* **167**, 056503 (2020).
3. Oh, N. K. et al. In–situ local phase-transitioned $MoSe_2$ in $La_{0.5}Sr_{0.5}CoO_{3-\delta}$ heterostructure and stable overall water electrolysis over 1000 hours. *Nat. Commun.* **10**, 1723 (2019).
4. Tong, C. et al. NiSe nanowire film supported on nickel foam: an efficient and stable 3D bifunctional electrode for full water splitting. *Angew. Chem. Int. Ed.* **127**, 9483–9487 (2015).
5. Xing, J. et al. Electro-synthesis of 3D porous hierarchical Ni–Fe phosphate film/Ni foam as a high–efficiency bifunctional electrocatalyst for overall water splitting. *J. Mater. Chem. A* **4**, 13866-13873 (2016).
6. Yang, Y. et al. Tuning electronic structures of nonprecious ternary alloys encapsulated in graphene layers for optimizing overall water splitting activity. *ACS Catal.* **7**, 469–479 (2017).
7. Inamdar, A. I. et al. A Robust Nonprecious CuFe Composite as a Highly Efficient Bifunctional Catalyst for Overall Electrochemical Water Splitting. *Small* **16**, 1905884 (2020).
8. Zhang, R. et al. Hydrolysis assisted in-situ growth of 3D hierarchical FeS/NiS/nickel foam electrode for overall water splitting. *Electrochim. Acta* **332**, 135534 (2020).
9. Shi, G. et al. Stabilization of cobalt clusters with graphdiyne enabling efficient overall water splitting. *Nano Energy* **74**, 104852 (2020).
10. Yu, F. et al. High–performance bifunctional porous non-noble metal phosphide catalyst for overall water splitting. *Nat. Commun.* **9**, 1-9 (2018).

11. Dinh, K. N. et al. Ultrathin porous NiFeV ternary layer hydroxide nanosheets as a highly efficient bifunctional electrocatalyst for overall water splitting. *Small* **14**, 1703257 (2018).
12. Zhu, W. et al. Nickel sulfide microsphere film on Ni foam as an efficient bifunctional electrocatalyst for overall water splitting. *Chem. Comm.* **52**, 1486-1489 (2016).
13. Zhang, H. et al. Bifunctional heterostructure assembly of NiFe LDH nanosheets on NiCoP nanowires for highly efficient and stable overall water splitting. *Adv. Funct. Mater.* **28**, 1706847 (2018).
14. Tang, T. et al. Electronic and morphological dual modulation of cobalt carbonate hydroxides by Mn doping toward highly efficient and stable bifunctional electrocatalysts for overall water splitting. *J. Am. Chem. Soc.* **139**, 8320-8328 (2017).
15. Jin, Y. et al. Porous MoO_2 nanosheets as non–noble bifunctional electrocatalysts for overall water splitting. *Adv. Mater.* **28**, 3785-3790 (2016).
16. Yu, L. et al. Cu nanowires shelled with NiFe layered double hydroxide nanosheets as bifunctional electrocatalysts for overall water splitting. *Energy Environ. Sci.* **10**, 1820–1827 (2017).
17. Wu, Y. et al. Cr-Doped FeNi–P Nanoparticles Encapsulated into N–Doped Carbon Nanotube as a Robust Bifunctional Catalyst for Efficient Overall Water Splitting. *Adv. Mater.* **31**, 1900178 (2019).
18. Song, H. J. et al. 3D Architectures of Quaternary Co–Ni–S–P/Graphene Hybrids as Highly Active and Stable Bifunctional Electrocatalysts for Overall Water Splitting. *Adv. Energy Mater.* **8**, 1802319 (2018).
19. Yu, J. et al. Reverse Microemulsion–Assisted Synthesis of $NiCo_2S_4$ Nanoflakes Supported on Nickel Foam for Electrochemical Overall Water Splitting. *Adv. Mater. Interfaces* **5**, 1701396 (2018).

20. Wang, X. et al. Bifunctional nickel phosphide nanocatalysts supported on carbon fiber paper for highly efficient and stable overall water splitting. *Adv. Funct. Mater.* **26**, 4067–4077 (2016).
21. Guo, P. et al. A highly stable bifunctional catalyst based on 3D $Co(OH)_2$@NCNTs@NF towards overall water–splitting. *Nano Energy* **47**, 96–104 (2018).
22. Sivanantham, A. et al. Hierarchical $NiCo_2S_4$ nanowire arrays supported on Ni foam: an efficient and durable bifunctional electrocatalyst for oxygen and hydrogen evolution reactions. *Adv. Funct. Mater.* **26**, 4661–4672 (2016).
23. Zhang, Y. et al. Cobalt–molybdenum nanosheet arrays as highly efficient and stable earth–abundant electrocatalysts for overall water splitting. *Nano Energy* **45**, 448–455 (2018).
24. Stolten, D. & Emonts, B. *Hydrogen Science and Engineering: Materials, Process, Systems, and Technology* (John Wiley & Sons, 2016)
25. Rong, X. et al. A Fundamental Relationship between Reaction Mechanism and Stability in Metal Oxide Catalysts for Oxygen Evolution. *ACS Catal.* **6**, 1153–1158 (2016).
26. Yao, Y. et al. Engineering the electronic structure of single atom Ru sites via compressive strain boosts acidic water oxidation electrocatalysis. *Nat. Catal.* **2**, 304–313 (2019).
27. Huang, Z. F. et al. Chemical and structural origin of lattice oxygen oxidation in Co–Zn oxyhydroxide oxygen evolution electrocatalysts. *Nat. Energy* **4**, 329–338 (2019).
28. Seo, M. H. et al. Design of Highly Active Perovskite Oxides for Oxygen Evolution Reaction by Combining Experimental and ab Initio Studies. *ACS Catal.* **5**, 4337–4344 (2015).
29. Retuerto, M. et al. Na-doped ruthenium perovskite electrocatalyst with improved oxygen evolution activity and durability in acidic media. *Nat. Comm.* **10**, 2041 (2019).
30. Miao, X. et al. Quadruple perovskite ruthenate as a highly efficient catalyst for acidic water oxidation. *Nat. Comm.* **10**, 3809 (2019).

31. Gauthier, J. A. et al. Challenges in Modeling Electrochemical Reaction Energetics with Polarizable Continuum Models. *ACS Catal.* **9**, 920–931 (2019).

32. Zhang, Q. et al. A. Solvation effects on DFT predictions of ORR activity on metal surfaces. *Catal. Today* **323**, 35–43 (2019).

33. Zhang, J. et al. Comparison of Implicit and Explicit Solvent Models for the Calculation of Solvation Free Energy in Organic Solvents. *J. Chem. Theory Comput.* **13**, 1034-1043 (2017).

34. Ping, Y. et al. The Reaction Mechanism with Free Energy Barriers at Constant Potentials for the Oxygen Evolution Reaction at the IrO_2 (110) Surface. *J. Am. Chem. Soc.* **139**, 149-155 (2017).
