# Supplementary material for: Highly efficient and robust noble-metal free bifunctional water electrolysis catalyst achieved via complementary charge transfer
Source: Nat Commun. 2021 Jul 29;12:4606. doi: 10.1038/s41467-021-24829-8 (PMC8322133; doi:10.1038/s41467-021-24829-8)
Supplement: Supplementary file 4 — Description of additional supplementary files [file 41467_2021_24829_MOESM4_ESM.docx]

**Description of Additional Supplementary File**

File Name: Supplementary Movie 1

Description: Overall water electrolysis test of LSC/K-MoSe_2_ || LSC/K-MoSe_2_
